# Palaeoenvironments and hominin evolutionary dynamics in southeast Asia

**DOI:** 10.1038/s41598-023-43011-2

**Published:** 2023-09-27

**Authors:** Anne-Marie Bacon, Nicolas Bourgon, Elise Dufour, Fabrice Demeter, Clément Zanolli, Kira E. Westaway, Renaud Joannes-Boyau, Philippe Duringer, Jean-Luc Ponche, Mike W. Morley, Eric Suzzoni, Sébastien Frangeul, Quentin Boesch, Pierre-Olivier Antoine, Souliphane Boualaphane, Phonephanh Sichanthongtip, Daovee Sihanam, Nguyen Thi Mai Huong, Nguyen Anh Tuan, Denis Fiorillo, Olivier Tombret, Elise Patole-Edoumba, Alexandra Zachwieja, Thonglith Luangkhoth, Viengkeo Souksavatdy, Tyler E. Dunn, Laura Shackelford, Jean-Jacques Hublin

**Affiliations:** 1 Université Paris Cité, CNRS, BABEL UMR 8045, 75012 Paris, France; 2 IsoTROPIC Research Group, Max Planck Institute for Geoanthropology, 07745 Jena, Germany; 3 Max Planck Institute for Evolutionary Anthropology, Department of Human Evolution, 04103 Leipzig, Germany; 4 UMR 7209 Archéozoologie, Archéobotanique, Sociétés, Pratiques, Environnements, MNHN, CNRS, Paris, France; 5 Lundbeck Foundation GeoGenetics Centre, Globe Institute, University of Copenhagen, Copenhagen, Denmark; 6 Eco-anthropologie (EA), MNHN, CNRS, Université Paris Cité, Musée de l’Homme, 75016 Paris, France; 7 Univ. Bordeaux, CNRS, MCC, PACEA, UMR 5199, 33600 Pessac, France; 8 ‘Traps’ Luminescence Dating Facility, School of Natural Sciences, Macquarie University, Sydney, Australia; 9 Geoarchaeology and Archaeometry Research Group (GARG), Southern Cross University, Lismore, NSW Australia; 10 Ecole et Observatoire des Sciences de la Terre, Institut de Physique du Globe de Strasbourg, UMR 7516 CNRS, Université de Strasbourg, Strasbourg, France; 11 Laboratoire Image, Ville Environnement, UMR 7362 UdS CNRS, Université de Strasbourg, Strasbourg, France; 12 Flinders Microarchaeology Laboratory, Archaeology, College of Humanities and Social Sciences, Flinders University, Sturt Road, Bedford Park, Adelaide, SA 5042 Australia; 13 Spitteurs Pan, Technical Cave Supervision and Exploration, La Chapelle en Vercors, France; 14 Institut des Sciences de l’Évolution de Montpellier, Univ Montpellier, CNRS, IRD, Montpellier, France; 15 Ministry of Information, Culture and Tourism, Vientiane, Lao PDR; 16 Institute of Archaeology, Hanoi, Vietnam; 17 Muséum d’histoire naturelle de La Rochelle, UMRU 24140 Dynamiques, interactions, interculturalité asiatiques (UBM, LRUniv), La Rochelle, France; 18 Department of Biomedical Sciences, University of Minnesota Medical School Duluth, Duluth, USA; 19 Anatomical Sciences Education Center, Oregon Health & Sciences University, Portland, OR USA; 20 Department of Anthropology, University of Illinois at Urbana-Champaign, Urbana, IL USA; 21 Carle Illinois College of Medicine, University of Illinois at Urbana-Champaign, Urbana, IL USA; 22 Chaire de Paléoanthropologie, CIRB (UMR 7241-U1050), Collège de France, Paris, France

**Keywords:** Evolution, Ecology

## Abstract

Secure environmental contexts are crucial for hominin interpretation and comparison. The discovery of a Denisovan individual and associated fauna at Tam Ngu Hao 2 (Cobra) Cave, Laos, dating back to 164–131 ka, allows for environmental comparisons between this (sub)tropical site and the Palearctic Denisovan sites of Denisova Cave (Russia) and Baishiya Karst Cave (China). Denisovans from northern latitudes foraged in a mix of forested and open landscapes, including tundra and steppe. Using stable isotope values from the Cobra Cave assemblage, we demonstrate that, despite the presence of nearby canopy forests, the Denisovan individual from Cobra Cave primarily consumed plants and/or animals from open forests and savannah. Using faunal evidence and proxy indicators of climates, results herein highlight a local expansion of rainforest at ~ 130 ka, raising questions about how Denisovans responded to this local climate change. Comparing the diet and habitat of the archaic hominin from Cobra Cave with those of early *Homo sapiens* from Tam Pà Ling Cave (46–43 ka), Laos, it appears that only our species was able to exploit rainforest resources.

## Introduction

**Figure 1:**
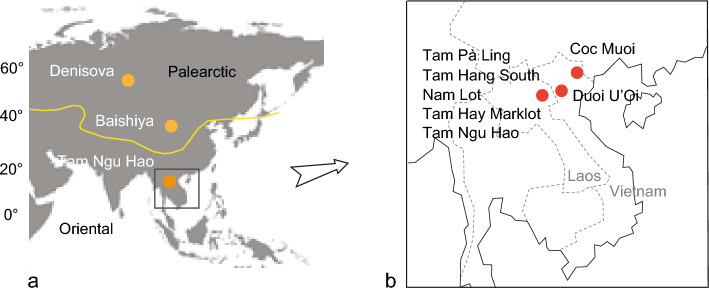
(**a**) Location of key sites of Denisovans in Russia (Denisova Cave), China (Baishiya Karst Cave), and Laos (Tam Ngu Hao (Cobra) Cave). The yellow line represents the biogeographic limit between the Palearctic and Oriental realms. (**b**) Location of the southeast Asian mammalian assemblages used in the study: Tam Pà Ling, Tam Hang South, Nam Lot, Tam Hay Marklot, and Tam Ngu Hao (Cobra) in northeastern Laos; Coc Muoi and Duoi U’Oi in Vietnam. Only three sites produced hominin remains: Tam Ngu Hao (Cobra) Cave (Denisovan, 164–131 ka), Tam Pà Ling (*H. sapiens*, 86–43 ka), and Duoi U’Oi (*Homo* sp., 70–60 ka) [Source: (**a**) Base map from https://capcarto.fr, (**b**) Authors].

The Denisovans were initially identified through their genome, which was extracted from a handful of finger bones, teeth, and sedimentary DNA from Denisova Cave in southern Siberia, Russia^1–7^. Additional evidence has since emerged from the analysis of ancient proteins and the morphology of a partial mandible^8^, as well as sedimentary DNA from the Baishiya Karst Cave in Xiahe, China^9^ (Fig. 1). Other populations, such as the Xujiayao hominins from northern China (identified through a set of teeth^10^), the Penghu 1 individual from Taiwan (identified through a mandible^11^) and the cranium from Harbin, China, known as *Homo longi* (proposed as a putative new species^12^) have been suggested as potential Denisovans. Recently, a molar of a young female Denisovan was discovered in the Tam Ngu Hao 2 Cave (Cobra Cave), Laos, with an age range of 164–131 ka^13^, with morphological similarity to that of molars of the Baishiya Karst Cave mandible (Supplementary Fig. S1).

Palaeogenetic evidence suggests that the Denisovans have the physiological capacity to live in high-altitude hypoxic environments^14^. This feature likely resulted from their adaptation to the extreme conditions of the Tibetan plateau from around 160,000 years ago (160 ka)^8^ up to approximately 60 ka^9^. Baishiya Karst Cave, where Denisovan remains were discovered, is located at an altitude of 3280 m above sea level (asl), which is much higher than Denisova Cave in the foothills of the Altai Mountains (700 m asl) or that of Cobra Cave in the karstic mountains of northeastern Laos (1116 m asl). Furthermore, the high rate of introgression of Denisovan DNA in the genome of modern populations from New Guinea, east Indonesia, the Philippines (Mananwa population), and Australia strongly suggests that Denisovans were present in southern and/or southeast Asia^15^. The locations and age estimates of the sites where Denisovans were unearthed therefore indicate that from around 200 ka to 50 ka, they adapted to a variety of environments ranging from temperate habitats in the Altai^5^ to tropical habitats in southeast Asia^13^.

Ancient DNA analyses indicate that Neandertals, Denisovans, and *Homo sapiens* interbred several times in the Middle to Late Pleistocene, throughout their evolution in Eurasia^6,16–22^. A major gene flow event between Neandertals and early *H. sapiens*^23^ likely occurred in the Levant > 170 ka^24^, whereas the Neandertal contribution to modern-day humans is constrained by the timing of the dispersal of our species outside Africa after 60–50 ka^25^. Similarly, Denisovans contributed up to ~ 4–6% to the genomes of ancestors of present-day Melanesian and Australasian populations^15^ and ~ 0.2% to the genomes of ancestors of mainland Asians and Native Americans^17^. The timing of interbreeding events between Denisovans and *H. sapiens* in Asia remains unclear. Palaeogenomic evidence indicates that interbreeding occurred over 50,000 years ago in the northern areas of the Denisovan distribution^7^. However, another study suggests that interbreeding may have occurred much more recently in the southern regions^22^.

In tropical latitudes, the scarcity of hominin fossils, as well as hot and humid conditions implying difficulties in retrieving DNA sequences from both fossils and sediments, poses a challenge in addressing the population history of Denisovans and *H. sapiens*^26^. Our discovery at Cobra Cave^13^ provides a new opportunity to explore the interaction between Denisovans and low-latitude tropical environments. While the dearth of archaeological material limits direct assessment into potential adaptations to tropical rainforests, geochemical proxies such as stable isotopes can offer a valuable source of data. Based on the principle that animal tissues metabolise and incorporate or reflect the isotopic composition of their diet, carbon isotope analysis from tooth enamel can provide crucial information on palaeodiets and, therefore, on palaeoenvironments^27,28^. Furthermore, because of the broad ecological range of ruminant ungulate taxa (i.e., browser, mixed-feeder, or grazer) and their unpredictable responses to climate changes in southeast Asia^29^, only such proxy records can help reveal the structure of past ecosystems and, therefore, their level of heterogeneity. This holds particular significance because, during the Pleistocene, the environments of southeast Asia consisted of a diverse range of biomes that underwent continuous fluctuations ranging from closed-canopy forests to grasslands^30^.

Here, we present the first analysis of the carbon (*δ*^13^C_apatite_) and oxygen (*δ*^18^O) isotope composition of a broad spectrum of mammalian taxa (Artiodactyla, Perissodactyla, Proboscidea, Carnivora, Primates, and Rodentia), as well as the Denisovan individual from Cobra Cave (164–131 ka^13^), to describe its diet and habitat. The Denisovan tooth (TNH2-1) is a developing first or, more likely, second lower molar of a juvenile female individual who died between 3.5 and 8.5 years^13^ (Supplementary Fig. S2). The age at which Denisovans were weaned is not known, but evidence from their closest relatives, the Neandertals, suggests an early weaning process similar to that of extant humans^31,32^. Therefore, considering the fact that the isotope values of TNH2-1 were obtained from a sample at the bottom of the crown, the young girl from Cobra Cave likely consumed the same food as that of adults of the group.

The *δ*^13^C values of bioapatite are used to investigate palaeodiets based on values associated with C_3_-plants (trees, bushes, shrubs, and grasses) versus C_4_-plants (grasses, sedges), and their respective environments. The *δ*^13^C_carbon source_ values in the diet of animals were then calculated from *δ*^13^C_apatite_ (“Material and methods”^33^) to more accurately explore the proportions of these isotopically-distinct carbon sources over the period studied, including sub-partitioning biomes such as closed-canopy forests^34^. The *δ*^18^O values are used to contribute palaeoecological information related to variation in abiotic conditions (latitude, climate, temperature, moisture content, amount, and isotopic composition of precipitation^35^, and references therein). Thus, these directly complement *δ*^13^C values and provide additional insights into past conditions.

To investigate the Denisovans’ environments in temperate *versus* tropical regions in Marine Isotopic Stage [MIS] 6 (191–130 ka^36^), we compared habitats inferred from fauna and isotopic records from Cobra Cave with those inferred from fauna and pollen evidence from Denisova Cave over the same period (Main Chamber, layers 19–17, 151 ± 17–128 ± 13 ka^5^). We evaluated the habitats for the other Asian hominin *Homo erectus* on Java until ~ 120 ka^37^ and questioned to what extent the ecological niches of Denisovans and *H. erectus* were comparable. Some works over the last two decades refined the contours of the ecological niche of Indonesian *H. erectus*, which is clearly that of open habitats in lowland areas^38–43^. Furthermore, in an attempt to compare habitats and diets between the Denisovans from Cobra Cave and the earliest *H. sapiens* in the area, we used available data from Tam Pà Ling (TPL) Cave, the two sites being located ~ 300 m apart (Fig. 1). Isotopic data from TPL include the *H. sapiens* individual TPL-1 (the upper left molar of the partial skull of a young mature adult, dated to 46–43 ka^44,45^) (Supplementary Fig. S2) and a handful of herbivores’ teeth (Artiodactyla and Perissodactyla) recovered in the sedimentary section^46^ prior to 33 ka, i.e., before the settlement of the Last Glacial Maximum conditions^47^. Previous research has documented that it foraged in a highly forested habitat^46,47^.

In a second step, using carbon and oxygen faunal records from Cobra Cave along with a series of five Middle to Late Pleistocene faunas of comparable composition (Artiodactyla, Perissodactyla, Proboscidea, Carnivora, Primates, and Rodentia) from northern Vietnam and Laos^29,48,49^ (Fig. 1), we identified large-scale climatic shifts that have transformed the palaeoenvironments locally. Thus, despite a discontinuous and patchy record (Supplementary Table S1), the mammalian faunas from Cobra Cave (164–131 ka), Coc Muoi (148–117 ka), Tam Hang South (94–60 ka), Nam Lot I (86–72 ka), Duoi U’Oi (70–60 ka) and Tam Hay Marklot (38.4–13.5 ka) may nevertheless provide key insights into major changes of ecosystems—both functional (species diversity and abundance) and structural (distribution of ecological niches)—over the period and, therefore, into the adaptive capacity of hominins to novel environments. Overall, at the scale of continental and insular southeast Asia, the Middle Pleistocene is seen as a period of open habitats that favoured the settlement and expansion of archaic hominins^30^, whereas the Late Pleistocene is marked by the expansion of rainforests at the time of *H. sapiens’* dispersal events, thus revealing potentially two different adaptive strategies. But one may question what environment prevailed in northern Indochina’s latitudes. Given this background, the present study aims to describe the environmental contexts into which Denisovans and *H. sapiens* inhabited successively in the studied area, considering the new extended TPL chronology with evidence of earliest *H. sapiens* at least 68 ka ago^50^.

## Results

**Figure 2:**
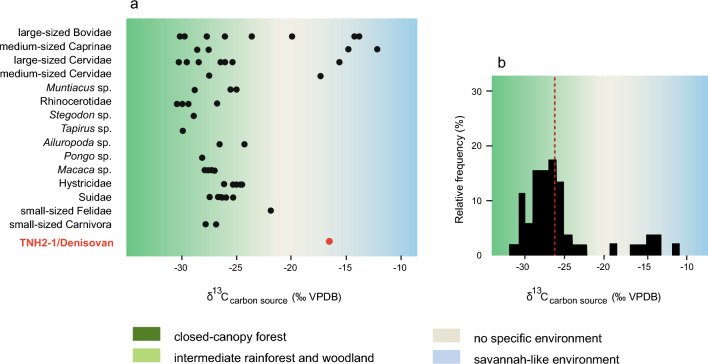
(**a**) Distribution of *δ*^13^C_carbon source_ values of animal and Denisovan specimens from Cobra Cave by taxon (see “Material and methods” for range of values associated with biomes). (**b**) Histogram distribution of the relative frequency (%) in *δ*^13^C_carbon source_ values for all taxa. Each bin represents a spacing of 1%. The dashed red line refers to the mean value (Source: Authors).

*The Cobra Cave Denisovan and associated fauna*: The *δ*^13^C_carbon source_ and *δ*^18^O_apatite_ values of every specimen are compiled in Supplementary Annex S1. As illustrated in Fig. 2, the *δ*^13^C_carbon source_ values for Cobra Cave range from − 31.3 to − 11.9‰ (average *δ*^13^C_carbon source_ =  − 25.18 ± 4.6‰ (1 σ), *n* = 54). The *δ*^18^O_apatite_ values for the site range from − 10.5 to − 2.6‰ (average *δ*^18^O_apatite_ =  − 6.7 ± 2.0‰ (1 σ), *n* = 54). The *δ*^13^C_carbon source_ and *δ*^18^O_apatite_ values of the Denisovan individual TNH2-1 are − 16.3‰ and − 7.0‰, respectively.

Post-hoc Dunn’s test pair-wise comparisons between the *δ*^13^C_carbon source_ values of Cobra Cave (164–131 ka) and those of the other sites (Coc Muoi, Tam Hang South, Nam Lot, Duoi U’Oi and Tam Hay Marklot) demonstrate significant differences only with Coc Muoi (148–117 ka) and Duoi U’Oi (70–60 ka) (Supplementary Tables S6, S7 and Annexes S3, S4). The *δ*^18^O_apatite_ values of Cobra Cave show no significant differences from those of the other faunas.

## Discussion

High latitude ecosystems, like those of Denisova Cave (151–128 ka; Main Chamber, Layers 19–17^5^, and Baishiya Karst Cave (~ 160 ka^8^; ~ 100 ka^9^), and the medium latitude ecosystem of Cobra Cave (164–131 ka^13^) harboured diverse herbivore communities. They encompass megafaunas adapted to very different environmental conditions, Palearctic *versus* Oriental^51^. This biogeographic division is reflected in the little commonality in taxonomic composition at the genus level between the cold-adapted *Mammuthus*-*Coelodonta* and the warm-adapted *Stegodon*-*Ailuropoda* faunal units (Fig. 1a and Supplementary Tables S8, S9).

What do we know about the biodiversity of Denisovans’ ecosystems? Figure 2 shows that at the latitude of Cobra Cave, the majority of the mammalian specimens (62%) exhibited *δ*^13^C_carbon source_ values not associated with closed-canopy forest (i.e., > − 27.2‰), thus rather reflecting intermediate and open woodland to savannah environments (“Material and methods”, Supplementary Table S5). Large ruminants (i.e., large bovines (*Bos*) and sambar deer (*Rusa*)) are those that predominantly foraged in this open landscape. We also note a gain in biodiversity among medium-sized ruminants due to the increased number of ecological niches^29^. Caprines such as gorals (*Naemorhedus*) and other medium-sized deer grazed on grasses in these open areas. In this ecosystem, the C_3_ canopy forests contained most of the other ground-dwelling herbivores (specimens having *δ*^13^C_carbon source_ values < − 27.2‰), including megaherbivores > 1000 kg^52^, tapirs (*Tapirus*), rhinoceroses (*Rhinoceros* and *Dicerorhinus*), and stegodon (*Stegodon*), while more open forests supported primates, macaques (*Macaca*) and orangutans (*Pongo*), wild boars (*Sus*), panda (*Ailuropoda*) and porcupines (*Hystrix*). At northern Indochina's latitudes, the Leizhou Peninsula pollen record^53^ reveals two major phases during MIS 6, with the latest half characterized by a relatively high percentage of Poaceae, which matches with the presence of savannah at Cobra Cave at the same period.

At the latitude of the Altai Mountains, environmental indicators also show a mosaic of biomes. The palynological evidence from the lower part of Layer 19 at Denisova Cave (starting ~ 168 ka^5^) suggests an association between meadows and steppe environments with forests composed of temperate elements (birch, pine, with a mixture of alder, linden, and elm) under relatively warm climatic conditions in the context of the Palearctic zone^5^. In this environment type, open landscapes such as tundra and steppe contained most of the megaherbivore biomass^54^ (*contra* tropical environment). Sedimentary DNA at Denisova indicates that the ‘mammoth’ steppe was occupied by non-ruminant grazers preferentially eating grasses and sedges (e.g., woolly rhinoceros (*Coelodonta*), woolly mammoths (*Mammuthus*)) with steppe bisons (*Bison*), and a large spectrum of gazelles (*Procapra, Saiga*), ibex (*Capra*) and argali (*Ovis*) adapted to grassy steppe and particularly abundant at that time (Layer 19^24^) (Supplementary Tables S9, S10). The occurrence of red deer (*Cervus elaphus*) and horse (*Equus* sp.) at the site also supports the presence of shrubs and trees, as indicated by the isotopic investigation conducted on these species from the Palearctic zone^55–57^.

The faunas from both the Tibetan Plateau and Altai Mountain share a common Palearctic origin (Fig. 1a). Today, the community of large herbivores adapted to live in high altitudes in excess of 3500 m consist primarily of medium-sized cervids, red deer (*Cervus*) and Siberian roe (*Capreolus*), medium-sized bovids (e.g., gazelle (*Procapra*), argali (*Ovis*), goral (*Naemorhedus*) and serow (*Capricornis*), and only one large bovid, the yak (*Poephagus*/*Bos*) (Supplementary Tables S8, S9). At the precise location of the Baishiya Karst Cave, the foothills of the mountain are dominated by alpine meadows composed of a variety of grasses, sedges, and herbs, whereas some wooded areas are present in riparian environments and along the mountain slopes^9^. The analysis of mtDNA from Late Pleistocene sediments of the Baishiya Karst Cave revealed that ~ 100 ka Denisovans lived within a richer herbivore community than today, dominated by rhinocerotids and equids that are now absent at high altitudes and with large bovids and cervids^9^ (Supplementary Tables S9, S10, S11).

**Figure 3:**
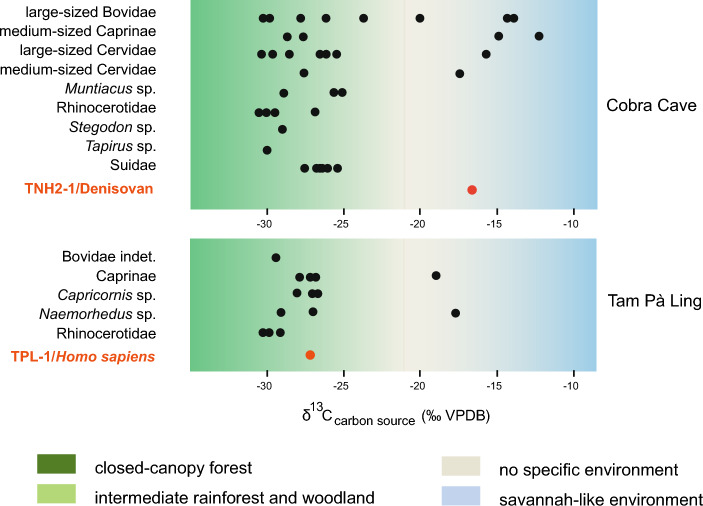
Distribution of *δ*^13^C_carbon source_ values of ungulate and hominin specimens from two sites (see “Material and methods” for range of values associated with biomes): Tam Ngu Hao (Cobra) Cave (Denisovan TNH2-1, 164–131 ka) (Supplementary Annex S1) and Tam Pà Ling (*Homo sapiens* TPL-1, 46–43 ka)^46^ (Supplementary Annex S2) (Source: Authors).

The known Denisovan populations occupying either temperate or tropical environments could therefore predate on a wide choice of herbivores. At the Denisova and Baishiya sites, herbivore remains have been found associated with abundant Palaeolithic stone artefacts^8,9^, and with direct evidence of human activities suggested by animal bones with cut-marks^8^. In the absence of comparable archaeological evidence, the *δ*^13^C_carbon source_ value of the Denisovan individual from Cobra Cave can be used to assess its diet. It reflects the consumption of plants and/or animals from open landscapes (*δ*^13^C_carbon source_ =  − 16.3‰; Fig. 3). Around Cobra Cave, open landscapes favoured the range expansion of mixed-feeders and grazers (bovines, caprines, deer), which exposed a diversified large game to hominin predation. This might have resulted in Denisovans foraging preferentially in open areas at the fringes of nearby forests, although a dense canopy forest was present in the environment.

When comparing the carbon isotope value of the Denisovan individual from Cobra Cave (164–131 ka) with that already published of the *H. sapiens* individual (46–43 ka^46^) from nearby TPL site^44^, the results display notable differences. The TPL individual data (*δ*^13^C_carbon source_ = − 26.4‰; Fig. 3) reflect a food procurement strategy that preferentially selects a C_3_ forest biome, possibly from a dense canopy forest. Various caprines (goral and serow), rhinoceroses, and large bovids^46^ are associated to this biome (Fig. 3, “Material and methods”, Supplementary Annex S2). We also infer patches of open vegetation with C_4_ plants based on two caprine teeth, further supported by the isotopic composition of *Camaena massiei* shells, a terrestrial gastropod, over the period 70–33 ka^47^ (Supplementary Text and Fig. S3). Therefore, conversely to the Denisovan individual from Cobra Cave, the TPL *H*. *sapiens* consumed food from a more forested area.

Despite the scarcity of archaeological material and poor organic matter preservation in tropical latitudes, we now have evidence of rainforests occupation by *H. sapiens* in Asia by ~ 70 ka^45,50,58,59^ and increasing evidence underlying the reliance on diverse settings ~ 45 ka^46,60–62^. *δ*^13^C values of *H. sapiens* from all these sites highlight the capacity of our species to adopt various behaviours in similar environments where both C_3_ forest and C_4_ open biomes are present in the vicinity. The data suggest specialization, such as hunting of arboreal species^63^, use of coastal resources^64^, opportunistic use of resources from mosaic and/or open forest edge environments^65^. In relation to *Homo sapiens* from TPL ~ 46 ka, reliance on deep forest resources would suggest the exploitation and processing of plants^60,66–68^ and the use of diverse hunting strategies such as traps, microlithics, and other tools made of organic material^60,61,63,69,70^. In contrast, the Denisovan from Cobra Cave, as well as the other archaic hominin *Homo erectus* from Java^41,43^, exhibit *δ*^13^C values that are solely indicative of a dietary reliance on open environments. Furthermore, while Denisovans adapted to diverse climates and habitats (i.e., from high latitudes at Denisova Cave and Baishiya Cave to medium latitudes at Cobra Cave)^6,8,9,13,22,71^, their reliance on grassland and woodland resources seemingly persisted.

The evolutionary path of *H. sapiens* since ~ 300 ka is marked by both a structural and genomic reorganization of the brain and a moderate increase in its size, in comparison with other contemporaneous large-brained hominins such as Neandertals and Denisovans^72^. Meyer et al.^16^ identified derived genomic features in *H. sapiens* that are not present in Denisovans and showed that some substitutions on human genes resulted in critical changes in brain function or nervous system development, notably greater synaptic plasticity in our species. That seems in accordance with southeast Asian palaeoenvironmental data, that suggests that *H. sapiens* expansions involved reliance on biome-specific specializations (*versus* Denisovans or *H. erectus*), thanks to a unique ecological plasticity within the hominin clade^73^.

**Figure 4:**
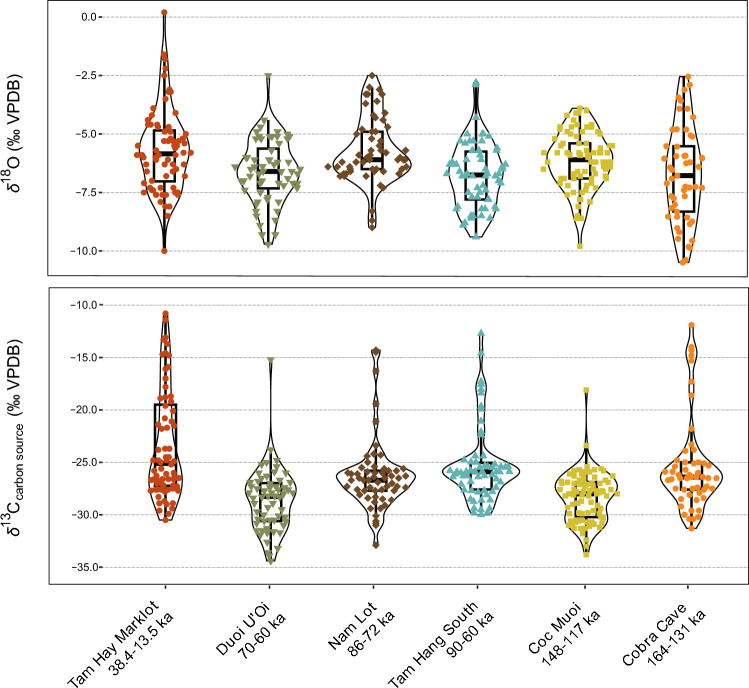
Distribution of *δ*^13^C_carbon source_ and *δ*^18^O values for all taxa in the southeast Asian faunas (Supplementary Annexes S1, S3, S4) from Tam Hay Marklot^48^, Duoi U'Oi^29^, Nam Lot^49^, Tam Hang South^29^, Coc Muoi^29^, and Tam Ngu Hao (Cobra) Cave (this paper). All sites were found within a relatively narrow latitudinal belt between 23° and 20° running through the northern regions of Laos and Vietnam (Fig. 1). The sites follow a chronological order from left to right highlighting environmental changes through vegetation cover variation (*δ*^13^C_carbon source_) and likely rainfall regimes (*δ*^18^O). The outline of the violin plots represents kernel probability density, where the width shows the proportion of the data found there. The boxes from the box and whisker plots inside the violin represent the 25th–75th percentiles, with the median as a bold horizontal line (Source: Authors).

In a previous analysis^29^, we revealed that late Middle to Late Pleistocene ecosystems were locally dynamic and diverse, based on notable changes in the distribution of *δ*^13^C_carbon source_ values (vegetation cover) in a series of faunas geographically close (Fig. 1b). This is confirmed here with the new data of Cobra Cave (164–131 ka) as illustrated in Fig. 4 by using violin plots, and further supported by statistical data with significant differences with Coc Muoi (148–117 ka) and Duoi U’Oi (70–60 ka) (Supplementary Table S6). Overall, *δ*^18^O_apatite_ values (rainfall regime) show a general trend towards higher values from Cobra Cave (164–131 ka) to Nam Lot (86–72 ka), before a change likely related to increased aridity over the Last Glacial period from ~ 70 ka, as suggested by the Duoi U’Oi (70–60 ka) and Tam Hay Marklot records (38.4–13.5 ka) (but not statistically significant) (Supplementary Table S7).

**Figure 5:**
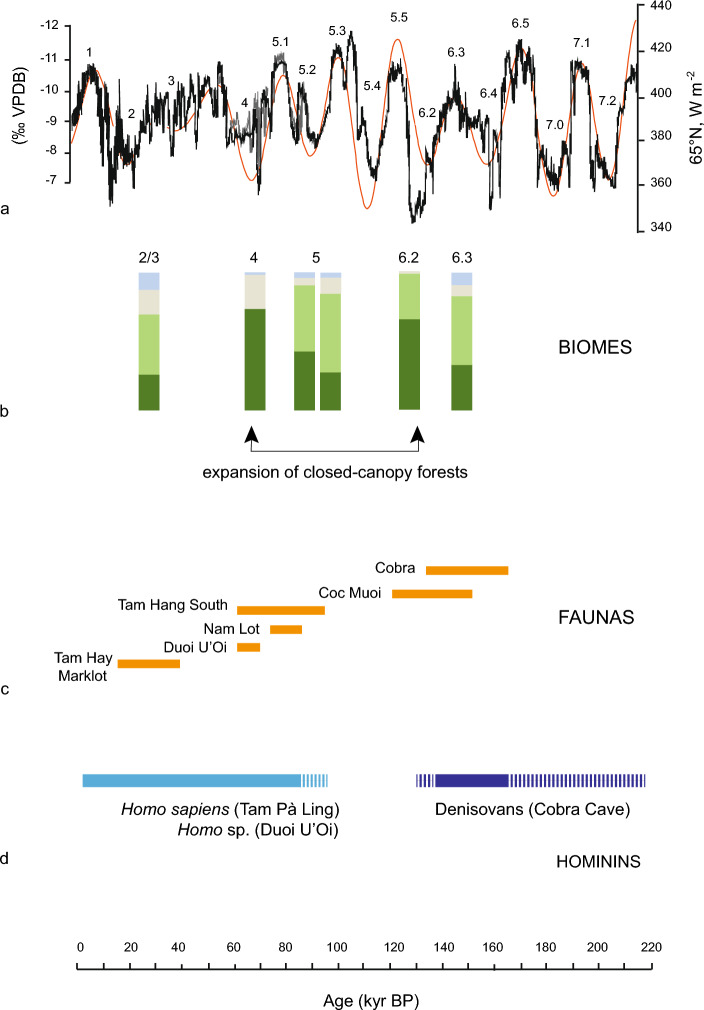
(**a**) Chinese caves *δ*^18^O records (left ordinate axis, ‰ VPDB International Standard Vienna Pee Dee Belemnite) showing millennial-scale climate shifts related to changes in East Asian summer monsoon intensity for the last 224 ka (black line) published in 2008 by Wang et al.^75^. Numbers refer to the marine isotope stages and substages. The right ordinate axis corresponds to the Northern Hemisphere summer insolation (red line) (65°N, W m^−2^). (**b**) Histograms of faunas showing the distribution of *δ*^13^C_carbon source_ values of specimens associated with each biome (Supplementary Table S5). (**c**) Age ranges of faunas: Tam Ngu Hao (Cobra) Cave (MIS 6.3, 164–131 ka) analyzed herein, Coc Muoi (MIS 6.2, 148–117 ka), Tam Hang South (MIS 5, 94–60 ka), Nam Lot (MIS 5, 86–72 ka), Duoi U’Oi (MIS 4, 70–60 ka) and Tam Hay Marklot (MIS 3–2, 38.4–13.5 ka). (**d**) Occurrence of hominins in the region, Denisovan (Cobra Cave, 164–131 ka), *Homo* sp. (Duoi U’Oi, 70–60 ka) and *H. sapiens* (Tam Pà ling, 86–43 ka) [Source: (**a**) Modified from Wang et al.^75^; (**b**–**d**) Authors].

By enabling the reconstruction of past environments, faunal isotopic data can, therefore, be useful tools to identify external drivers of hominin evolution, even if correlating chronologies between faunas constrained by luminescence dating and better chronologically constrained palaeoclimatic signals from the speleothem records reveals challenging^74^ (“Material and methods”). With regards to the region studied, we used the *δ*^18^O curves from speleothems of the nearest Chinese reference sites as indicators of the intensity of East Asian summer monsoon^75^ (Fig. 5a) and histograms of the distribution of *δ*^13^C_carbon source_ values associated with the different biomes inferred from each fauna (Supplementary Table S5, Fig. 5b).

Our results highlight two repeated episodes of rainforest expansion as climates fluctuated (Fig. 5b). Each episode was a singular event that led to novel plant communities and structures (i.e., the density of canopy, shrub, and floor strata). The first one, which occurred at the time of the dispersal of *H. sapiens* has been documented previously by Bacon et al.^29^, based on changes in the distribution of biomes between Nam Lot (MIS 5, 86–72 ka) and the *Homo* sp.-bearing site of Duoi U’Oi (MIS 4, 70–60 ka) (Fig. 5b,c). Overall, MIS 5 was a period of relatively strong monsoons and high precipitation^69^, associated with a mosaic of biomes. It was followed by a rapid decrease in monsoon strength at the onset of MIS 4 (Fig. 5a). At that time, a rapid forest transformation resulted in an increase of temperate forest elements, most notably conifers, in a relatively cooler climate^53^. These changes were also accompanied by a novel type of shrub, fern, and herb strata^53^ that likely rendered the forests easier for hunter-gatherers to navigate and forage^29^. The presence of *H. sapiens* in the region has been confirmed recently by the extended chronology of Tam Pà Ling at least 68 ka^50^. Further evidence for the ability of *H. sapiens* to occupy a broad spectrum of rainforests comes from Lida Ajer, Sumatra (71–68 ka)^59^. At this latitude, humans exploited a landscape dominated by closed-canopy forests (based on the isotopic faunal record, Supplementary Fig. S4 and Annex S7) that were not so different from the equatorial forests of Sumatra today.

**Figure 6:**
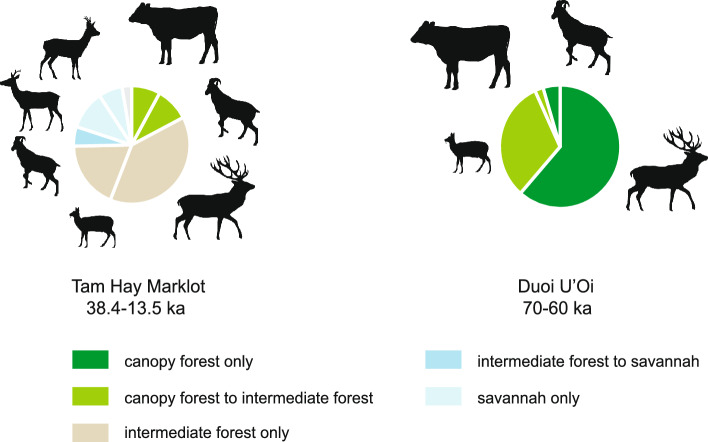
Compared biodiversity between Duoi U’Oi (MIS 4, 70–60 ka) and Tam Hay Marklot (MIS 3–2, 38.4–13.5 ka) faunas. We combined in a same scheme the 3 descriptors of biodiversity, species diversity among identified ruminant taxa (i.e., cervids and bovids being able to browse and/or graze, Supplementary Tables S2, S3), species abundance (by using percentages of the minimum number of individuals (MNI), Supplementary Tables S12), and distribution of ecological niches (biome associated with each taxon) (Source: Silhouette image from public domain https://www.phylopic.org; charts, Authors).

Furthermore, the comparison in the distribution of biomes between Duoi U’Oi (MIS 4, 70–60 ka) and Tam Hay Marklot (MIS 3–2, 38.4–13.5 ka) (Fig. 6) shows how dramatic were the environmental changes over MIS 4–2^47^, a period that accompanied the dispersal of hunter-gatherers through the savannah corridor^65^. Duoi U’Oi witnesses low biodiversity and this ecosystem dominated by canopy forests favored the abundance of the sambar deer (61.3%) and muntjacs (31.8%) (Supplementary Table S12). Tam Hay Marklot shows that the diversity of herbivores increased as landscapes opened and biomes diversified, owing to the increased number of ecological niches. This gain of biodiversity, most likely through dispersal events, concerns various deer (17.2%) known to live in large herds in open areas and caprines (5.3%). Their relative abundance also points to the extent of grassland and its important carrying capacity, as also observed at the same latitude in Tham Lod Rockshelter (34–12 ka)^65^, a unique condition not observed in our MIS 6–5 faunal records (Supplementary Fig. S5).

Another episode is highlighted here for the first time by the changes in the distribution of biomes between Cobra (164–131 ka) and Coc Muoi (148–117 ka) (Fig. 5b), a period that likely impacted archaic hominins locally (Fig. 5d). Cobra Cave clearly provides evidence for the presence of savannah and woodland savannah biomes along with fragmented rainforests, and its age range coincides with the MIS 6.3 period of relatively strong monsoons (Fig. 5a). This environment was established ~ 160 ka when grassy areas, notably composed of Poaceae and Cyperaceae, replaced *Artemisia* steppe^76^. The shift in vegetation cover documented by the expansion of the canopy forest biome at Coc Muoi (148–117 ka), seems to coincide with the weaker monsoon interval 135.5–129 ka (MIS 6.2 in the Hulu/Sanbao records^75^). This shift can be associated with the abrupt reappearance of forest montane elements in lowland zones with cool and relatively wet conditions^53^.

Considering the subsistence strategy of the Denisovan individual from Cobra Cave, could this cold event have been a driving factor in archaic hominin evolution? If data from a single individual cannot fully reflect the diversity of the subsistence of the entire group locally, the fact that it relied on mixed to open landscapes for food resources supports the idea that these biomes might have played a significant role in the mobility and settlement of this archaic hominin in a tropical ecosystem^77^. This raises the question of how they adapted to climate changes ~ 130 ka that resulted in the expansion of rainforests. Foragers faced different challenges in forest biomes based on their behavioural flexibility, and archaic hominins may have experienced a population contraction in response to the emergence of dense rainforests^78^.

It is tempting to draw a parallel with the history of *Homo erectus*. On Java, the pollen record from Sangiran around 800 ka documents the settlement of a landscape dominated by grasslands while rainforests underwent a severe fragmentation in high altitude areas, river streams, and swamps^39^. Although *H. erectus* occupied a mosaic of habitats with grassland on Java from ~ 1.2 Ma^79^, this vegetational change ~ 800 ka is associated with a greater abundance of hominin remains along with the spread of Acheulean-like industries^40^, a settlement of populations likely favoured by this landscape. For subsequent periods, the carbon isotope analysis of faunas from *H. erectus*-bearing sites Trinil H.K. (540–430 ka^80^) and Ngandong (117–108 ka^37^) suggests a mixed woodland-savannah environment^41,42^ (Supplementary Fig. S4 and Annexes S5, S6). At these lower latitudes, a major biogeographical event led to the dispersal of a rainforest-adapted fauna resulting from a lowstand sea level of up to – 120 m^81^. This is documented by the replacement of the Ngandong archaic fauna (117–108 ka) by the fully modern Punung fauna (128–118 ka^82,83^), but the synchronicity with the climatic episode that led to rainforest expansion ~ 130 ka farther north remains to be demonstrated. Ngandong also witnessed the last occurrence of *H. erectus*^37^, raising the question of a population range contraction in more suitable areas on Sundaland shortly before its extinction*.*

## Conclusion

The present study highlights that ecosystems occupied by Denisovans, whether temperate or tropical, shared mixed vegetation covers with a significant part of open landscapes. At Cobra Cave, the presence of open woodlands and savannahs promoted a high diversity of herbivores, with a notable expansion in the range of cervids and bovids through increased niche partitioning between taxa. Despite the presence of closed forested areas, Denisovans likely preferentially targeted large game visible in open areas or at forest edges. In contrast, our results suggest that early *Homo sapiens* of the same region had a different ecological niche that relied on rainforests at least ~ 70 ka, most likely due to the development of new behavioral skills.

Hence, our findings are relevant to the debate regarding the potential role of rainforests as a primary driver of hominin evolution in southeast Asia and raise the question of whether the expansion of rainforests acted as a regional barrier to Denisovans. Recent genomic analyses reveal multiple Denisovan groups that were geographically isolated from each other during the Pleistocene in southeast Asia. The repeated episodes of rainforest expansion might have played a key role in this population contraction range that shaped the hominin evolution.

## Material and methods

### Geographical and chronological context of Tam Ngu Hao 2 (Cobra) Cave

The karstic cave is located in northeastern Laos (Supplementary Fig. S1). The dates for breccia deposits in which the human tooth and faunal remains were found range between 164 and 131 ka^13^ based on Bayesian modelling of luminescence dating of sediments, uranium-series dating of flowstones, and coupled U-series and electron spin resonance dating of three bovid teeth. This time interval corresponds to the second half of Marine Isotopic Stage [MIS] 6 (191–130 ka^36^).

### Composition of the Cobra Cave assemblage

It is dominated by isolated teeth of large mammals (N = 186), including the Denisovan tooth (Supplementary Tables S2, S3). Due to the deposition in a context of high energy^13^, the selective conservation and poor preservation of most specimens constrained their identification at the genus or family level. Artiodactyla: *Sus scrofa* (*n* = 38), *Bos* sp. (*n* = 35), *Naemorhedus* sp. (*n* = 10), *Muntiacus* sp. (*n* = 24), medium-sized Cervidae (*n* = 6), large-sized Cervidae (*n* = 16); Perissodactyla: Rhinocerotina indet. (*n* = 12), *Rhinoceros* sp. (*n* = 3), *Rhinoceros sondaicus* (*n* = 2), *Dicerorhinus* sp. (*n* = 1), *Tapirus* sp. (*n* = 1); Proboscidea: *Stegodon* sp. (fragments of enamel); Carnivora: small-sized carnivora (*n* = 2), small-sized Felidae (*n* = 1), *Paradoxurus* sp. (*n* = 1), *Ursus thibetanus* (*n* = 3), *Ailuropoda* sp. (*n* = 2); Primates: *Macaca* sp. (*M*. cf. *nemestrina*) (*n* = 10), *Pongo* sp. (*n* = 1), Homininae (Denisovan) (*n* = 1); Rodentia: *Hystrix* sp. (*n* = 18).

### New sub-sample for isotopic analyses

A sub-sample of 54 specimens was selected within the Cobra Cave assemblage: *Sus scrofa* (*n* = 8), Large-sized Bovidae (*n* = 8), medium-sized Caprinae (*Naemorhedus* sp.) (*n* = 10), *Muntiacus* sp. (*n* = 3), medium-sized Cervidae (*n* = 2), large-sized Cervidae (*n* = 7), Rhinocerotidae (*n* = 3), *Rhinoceros* sp. (*n* = 1), *Tapirus* sp. (*n* = 1), *Stegodon* sp. (*n* = 1), small-sized carnivora (*n* = 2), small-sized Felidae (*n* = 1), *Ailuropoda* sp. (*n* = 2), *Macaca* sp. (*n* = 4), *Pongo* sp. (*n* = 1), Homininae (Denisovan) (*n* = 1), *Hystrix* sp. (*n* = 5) (Supplementary Tables S4, S5).

### Stable carbon and oxygen isotope data

In terrestrial food webs, analyses of stable carbon isotopes of bioapatite (*δ*^13^C_apatite_) are an effective way to assess the relative proportion in a consumer's diet of ingested carbon derived from food webs’ primary sources (plants) using either C_3_ or C_4_ photosynthetic pathways^84^. In tropical and subtropical regions specifically, C_4_ plants (grasses, sedges) are found in open environments and exhibit high *δ*^13^C values, whereas forests and woodland habitats are associated with C_3_ plants (trees, bushes, shrubs, and grasses) and low *δ*^13^C values^85^. Densely forested conditions induce even lower *δ*^13^C values in plants due to a "canopy effect"^86^, allowing the identification of additional ecological partitioning in C_3_ forested environments. Using *δ*^13^C_apatite_ values and diet-enamel spacing, the average initial *δ*^13^C values of the carbon source in the animal's diet (herein labeled as "*δ*^13^C_carbon source_") were estimated to allow more accurate environmental reconstructions. To account for the atmospheric CO_2_ shift due to fossil fuel burning, the *δ*^13^C ranges of values for C_3_ and C_4_ plants were adjusted accordingly (~ 1.3‰^87^). In Figs. 2, 3 and 5b**,** the *δ*^13^C_carbon source_ values associated with closed-canopy forests are <  − 27.2‰^34^, intermediate rainforests and woodland biomes >  − 27.2‰ and < − 21.3‰^88^, and savannah-like environments > − 15.3‰^84^. Values between > − 21.3‰ and < − 15.3‰ are associated with the consumption of both C_3_ and C_4_ resources and do not correspond to any specific ecological environment.

Stable oxygen isotopes of bioapatite (*δ*^18^O values) vary according to the oxygen isotopic composition of drinking water and chemically-bound water in diet (i.e., water found in plants) and are controlled by various environmental and geographic conditions such as latitude, climate, temperature, moisture content, amount and isotopic composition of precipitation^35^. At low latitudes, such as in the studied area, variations of *δ*^18^O rainfalls are primarily indicative of the amount of precipitation. Just as with *δ*^13^C values, a "canopy effect" can also be characterized by low *δ*^18^O values on the forest floor^86^.

Fossil teeth of the TNH2-1 individual and sympatric mammal specimens (*n* = 54) from Cobra Cave were sampled and analyzed for the present study (Supplementary Annexes S1–S3). Using a handheld dental drill equipped with a diamond-tipped burr, the enamel surface of each specimen was cleaned mechanically, and powder samples were subsequently taken along the full height of the crown. Powdered enamel teeth samples were subsequently pretreated to remove exogenous carbonate. Samples were thus soaked in 1 ml of CH_3_COOH (0.1 M) for 4 h at room temperature, rinsed several times in distilled water, and then dried overnight at 65 °C. Measurements of stable carbon and oxygen isotopic ratios of the carbonate phase of enamel were performed at the *Service de Spectrométrie de Masse Isotopique du Muséum* (SSMIM) in Paris using a Thermo Scientific Delta V Advantage isotopic mass spectrometer along with a Thermo Scientific Kiel IV Carbonate Device chemical preparer. Isotopic abundances are presented in delta (δ) notation and expressed as deviation per mil (‰), as follow: *δ*^13^C = (^13^C/^12^C_sample_/^13^C/^12^C_standard_ − 1) × 1000 and* δ*^*18*^*O* = (^18^O/^16^O_sample_/^18^O/^16^O_standard_ − 1) × 1000.

During every mass spectrometer run, we analyzed an internal laboratory standard (Marble LM, accepted *δ*^13^C =  + 2.13‰ and *δ*^18^O =  − 1.83‰) normalized to the International Atomic Energy Agency reference material NBS 18 and NBS 19. LM was used for tooth sample correction (one point-correction) and for controlling the precision (1σ) of the mass spectrometer (*δ*^13^C = 0.035‰ and *δ*^18^O = 0.051‰; n = 24). We usually analyzed each tooth sample one or two times. Four samples were analyzed three times to test for intra-individual heterogeneity and analytical reproducibility of enamel analysis. Maximum standard deviations were 0.527‰ and 0.342‰ for *δ*^13^C_enamel_ and *δ*^18^O analysis, respectively.

### Palaeoenvironmental reconstruction

For the purpose of this study—the comparison between the landscapes inhabited by Denisovans and *H. sapiens* locally—we used published isotopic data from Tam Pà Ling, including the TPL-1 individual (46–43 ka) and a handful of herbivore teeth (Artiodactyla and Perissodactyla) recovered in the sedimentary section between 70 and 33 ka^46^ (Supplementary Annex S2). This range has been selected to document the environment of early *H. sapiens* locally before the major changes that began ~ 33 ka and led to the settlement of the Last Glacial Maximum conditions^47^ (Supplementary Text and Fig. S3).

In this study, original carbon (*δ*^13^C_carbon source_) and oxygen (*δ*^18^O) isotope values from tooth enamel of mammals from Cobra Cave (164–131 ka) are compared to our published data (Bacon et al., 2021, *Sci. Rep*.)^29^ of faunas of comparable composition (Artiodactyla, Perissodactyla, Proboscidea, Carnivora, Primates, and Rodentia) from the following sites: Coc Muoi (148–117 ka), Tam Hang South (94–60 ka), Nam Lot I (86–72 ka), Duoi U’Oi (70–60 ka), and Tam Hay Marklot (38.4–13.5)^29,48^ (Supplementary Tables S1–S4 and Annexes S3, S4).

Results of *δ*^13^C_carbon source_ values (‰ VPDB) from these six subsamples are used herein to build the histograms presented in Fig. 5b. They show the distribution (expressed as a percentage) of *δ*^13^C_carbon source_ values of specimens within each biome: closed-canopy forests (< − 27.2‰); intermediate rainforests and woodland biomes (> − 27.2‰ and < − 21.3‰); no specific ecological environment *(*> − 21.3‰ and < − 15.3‰); and savannah-like environments (> − 15.3‰) (Supplementary Table S5). Our goal here is to correlate the faunal isotopic data with available proxy indicators of climates. The palaeoclimatic data used from the last ~ 200,000 years are speleothem *δ*^18^O values recorded in the Sanbao/Hulu Chinese caves (Fig. 2^75^), which allow for tracking the fluctuation of the summer monsoon intensity for the period as shown in Fig. 5a. In this record, low speleothem *δ*^18^O values (‰, VPDB, left ordinate axis) correspond to low rainfall *δ*^18^O values and therefore to increases in precipitation, i.e., the amount effect^89^.

Correlating the chronologies between faunas constrained by luminescence dating and better chronologically constrained palaeoclimatic signals is challenging, particularly for assemblages with a wide age range like that of Tam Hang South (94–60 ka). However, the *δ*^13^C values of teeth selected from this assemblage could not result from a mixture with the Duoi U’Oi period (70–60 ka) as both subsamples show different distributions (Fig. 4), furthermore supported by statistics^29^. From our previous published analysis^29^, overall, results of the MIS 5 Tam Hang South and Nam Lot faunas are consistent and indicate a high level of environmental heterogeneity, showing a mosaic of habitats from closed-canopy forests to more open biomes during relatively strong monsoonal periods and high precipitation^75^. Supplementary Table S5 shows the percentages of teeth associated to the different biomes with 27.4% (C_3_), 69.2% (C_3_–C_4_), 3.2% (C_4_) for Tam Hang South and 42.1% (C_3_), 54.3% (C_3_–C_4_), 3.5% (C_4_) for Nam Lot I. During this period, C_3_–C_4_ forested to open landscapes were occupied by ruminant taxa thanks to their great dietary flexibility and capacity to shift from browsing to grazing (essentially *Rusa unicolor*, medium-sized cervids, large bovines *Bos* and *Bubalus*, and caprines *Capricornis* and *Naemorhedus*). The range of *δ*^13^C values of these taxa, the highest among herbivores, has been associated with either the variability of diet within a species or dietary flexibility according to seasons^65,90,91^. In these types of environments, the C_3_ canopy forests contained most of the megaherbivore biomass (*Tapirus*, *Megatapirus*, *Rhinoceros*, *Dicerorhinus*, *Elephas*, and *Stegodon*).

Our previous results also highlighted the potential of canopy forests for contraction during some periods. In our sample, two faunas illustrate these changes, Duoi U’Oi (70–60 ka) and Coc Muoi (148–117 ka), which match with significant drops of monsoon intensity at the onset of MIS 4 and the end of MIS 6, respectively. Data of the Supplementary Table S5 show a predominant proportion of C_3_ plants (up to ~ 73% at Duoi U’Oi), which corresponds to phases of expansion of the canopy rainforests: 73.3% (C_3_), 25% (C_3_–C_4_), 1.6% (C_4_) for Duoi U’Oi and 65.4% (C_3_), 34.4% (C_3_–C_4_), 0% (C_4_) for Coc Muoi. In these ecosystems, closed rainforests contained all of the mammalian biomass. Isotopic results^29^ and palynological proxies^53,76^ indicate major abiotic (rates of insolation, seasonality, amount of rainfall, etc.) and environmental changes with different types of forests (plant communities, structure of the vegetation, etc.) during these events of weak monsoons. Furthermore, an estimate of the abundance of herbivore taxa (Supplementary Fig. S6) also supports functional changes of ecosystems: that of Coc Muoi favoured rhinoceroses and large bovids, whereas that of Duoi U’Oi favoured large cervids.

### Statistical analyses

Kruskal–Wallis one-way analysis of variance and post-hoc Games-Howell pairwise comparisons (Supplementary Tables S6, S7) were performed across the dataset to identify statistical differences in *δ*^13^C_carbon source_ and *δ*^18^O_apatite_ values between sites (and thus also periods). Preliminary tests and visual inspection were carried out to check for normally distributed data and equal variance, which revealed that non-parametric testing (i.e., Kruskal–Wallis) was preferred over parametric methods (i.e., ANOVA). A total of 6 different sites and 390 samples were used for the analyses: Cobra Cave (*n* = 55), Coc Muoi (*n* = 84)^29^, Tam Hang South (*n* = 62)^29^, Duoi U’Oi (*n* = 60)^29^, Nam Lot (*n* = 57)^49^ and Tam Hay Marklot (*n* = 72)^48^. All statistical analyses were conducted using the free program R software (version 4.2.2^92^) and packages “car” (version 3.1^93^), stats (version 3.6.2; R Core Team, 2022), tidyverse (version 1.3.2^94^), and ggplot2 (version 3.4^95^).

### Supplementary Information

Supplementary Information.

## Data Availability

All data generated and analysed during this study are included in this article and its supplementary file.
