# Supplementary material for: Palaeoenvironments and hominin evolutionary dynamics in southeast Asia
Source: Sci Rep. 2023 Sep 27;13:16165. doi: 10.1038/s41598-023-43011-2 (PMC10533506; doi:10.1038/s41598-023-43011-2)
Supplement: Supplementary file 1 — Supplementary Information. [file 41598_2023_43011_MOESM1_ESM.pdf]

Supplementary Information

Palaeoenvironments and hominin evolutionary dynamics in Southeast Asia

Anne-Marie Bacon[1*], Nicolas Bourgon[2,3], Elise Dufour[4], Fabrice Demeter[5,6], Clément Zanolli[7], Kira E. Westaway[8], Renaud Joannes-Boyau[9], Philippe Duringer[10], Jean-Luc Ponche[11], Mike W. Morley[12], Eric Suzzoni[13], Sébastien Frangeul[13], Quentin Boesch[10], Pierre-Olivier Antoine[14], Souliphane Boualaphane[15], Phonephanh Sichanthongtip[15], Daovee Sihanam[15], Nguyen Thi Mai Huong[16], Nguyen Anh Tuan[16], Denis Fiorillo[4], Olivier Tombret[4], Elise Patole-Edoumba[17], Alexandra Zachwieja[18], Thonglith Luangkhoth[15], Viengkeo Souksavatdy[15], Tyler E. Dunn[19], Laura Shackelford[20,21], Jean-Jacques Hublin[3,22]

[1]Université Paris Cité, CNRS, BABEL UMR 8045, 75012 Paris, France ; [2]IsoTROPIC research group, Max Planck Institute for Geoanthropology, 07745 Jena, Germany; [3]Max Planck Institute for Evolutionary Anthropology, Department of Human Evolution, 04103 Leipzig, Germany; [4]UMR 7209 Archéozoologie, Archéobotanique, Sociétés, Pratiques, Environnements, MNHN, CNRS, Paris, France; [5]Lundbeck Foundation GeoGenetics Centre, Globe Institute, University of Copenhagen, Copenhagen, Denmark; [6]Eco-anthropologie (EA), MNHN, CNRS, Université Paris Cité, Musée de l'Homme, 75016 Paris, France; [7]Univ. Bordeaux, CNRS, MCC, PACEA, UMR 5199, 33600 Pessac, France ; [8]'Traps' Luminescence Dating Facility, School of Natural Sciences, Macquarie University, Sydney, Australia; [9]Geoarchaeology and Archaeometry Research Group (GARG), Southern Cross University, NSW, Australia; [10]Ecole et Observatoire des Sciences de la Terre, Institut de Physique du Globe de Strasbourg, UMR 7516 CNRS, Université de Strasbourg, France; [11]Université de Strasbourg, Laboratoire Image, Ville Environnement, UMR 7362 UdS CNRS, France ; [12]Flinders Microarchaeology Laboratory, Archaeology, College of Humanities and Social Sciences, Flinders University, Sturt Road, Bedford Park, Adelaide, SA 5042, Australia; [13]Spitteurs Pan, technical cave supervision and exploration, La Chapelle en Vercors, France; [14]Institut des Sciences de l'Évolution de Montpellier, Univ Montpellier, CNRS, IRD, Montpellier, France; [15]Ministry of Information, Culture and Tourism, Laos PDR; [16]Institute of Archaeology, Hanoi, Vietnam; [17]Muséum d'histoire naturelle de La Rochelle, UMRU 24140 Dynamiques, interactions, interculturalité asiatiques (UBM, LRUniv), La Rochelle, France; [18]Department of Biomedical Sciences, University of Minnesota Medical School Duluth, USA; [19]Anatomical Sciences Education Center, Oregon Health & Sciences University, Portland, OR, USA; [20]Department of Anthropology, University of Illinois at Urbana-Champaign, Urbana, IL, USA; [21]Carle Illinois College of Medicine, University of Illinois at Urbana-Champaign, Urbana, IL, USA; [22]Chaire de Paléoanthropologie, CIRB (UMR 7241-U1050), Collège de France, Paris, France.

Location and geological setting of sites. The sites used in this article are located in northern Vietnam (Duoi U'Oi and Coc Muoi) and Laos (Nam Lot I, Tam Hang South, Tam Ngu Hao 2 (Cobra) Cave, Tam Pà Ling, Tam Hay Marklot), at the latitudes 23°-20° (Bacon et al., 2008, 2015, 2018; Duringer et al., 2012; Demeter et al., 2012, 2022; Shackelford et al., 2018; Bourgon et al., 2020) (Fig. S1). The landscape of the region contains typical tower karsts that emerge from the alluvial plain covered by cultivated fields and houses. All karstic sites formed in massive limestone beds, Carboniferous to Triassic in age. The deposits consist of either well-cemented breccias plastered on the walls and/or roofs of caves and/or silty to sandy clays located on the cave floor. The analysis of the sites (except Tam Pà Ling) suggests that the breccias were created principally by reworked carbonate clasts from the limestone massif, speleothems and sandy to silty clays, along with fossils. These fossil remains and sediments were carried and deposited by water inside the cave network (fossiliferous breccias mainly result from endokarstic processes mixed with variable exokarstic material) (Duringer et al., 2012). Fossiliferous deposits are most likely a result of a long transportation process of remains through the subterraneous cave networks over several thousands of years or more. Diverse dating techniques have been used to constrain the ages of faunas and associated hominins (Table SI1). The detailed description of sites can be found in original publications.

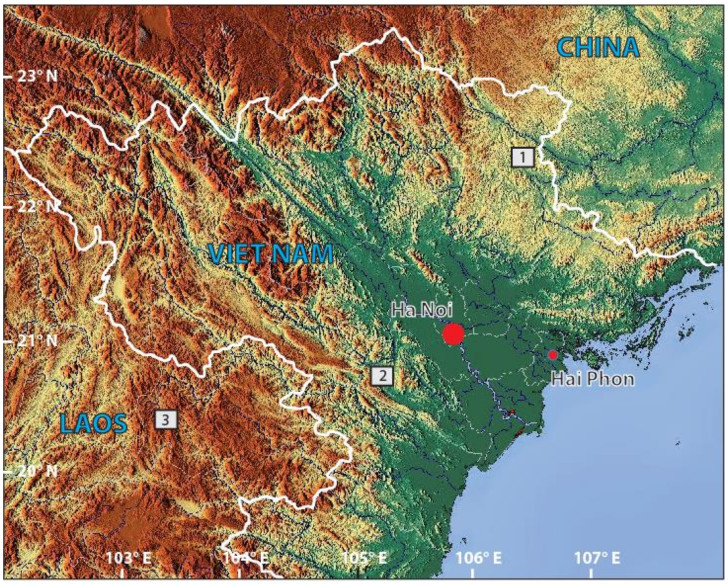

1. Coc Muoi
2. Duoi U'Oi
3. Nam Lot I - Tam Hang South - Tam Ngu Hao 2 - Tam Pà Ling - Tam Hai Marklot

Figure S1: Satellite image of the studied area with the location of sites in southeast Asia. The satellite image is from the website (http://www.maps-for-free.com/) and has been reworked by P. Duringer using the software Illustrator CS5 (version 15.0.0).

Conservation of assemblages and taphonomic biases: The faunal assemblages from Vietnam and Laos (Coc Muoi, Duoi U'Oi, Nam Lot, Tam Hang South, Tam Hay Marklot) contain mainly isolated teeth of a large array of mammals, including Artiodactyla, Perissodactyla, Proboscidea, Carnivora, Primates and Rodentia (>5kg). Small-bodied microvertebrates are lacking. Their analysis revealed comparable taphonomic pathways, due to a complex action of biotic (rodents and carnivores) and abiotic (water flows) agents, through comparable processes of deposition (Duringer et al., 2012).

The high percentages of teeth (> 70%) showing gnawing marks indicate the significant role of porcupines (*Hystrix* sp.), as the last accumulator agent of remains of carcasses at the sites, before being transported into the cave network and buried in the sediments. The differential preservation of lower *versus* upper teeth has been associated with the capacity of porcupines to collect all detachable and transportable remains of carcasses of animals of various body-sizes (~5 kg up to 5,000 kg), either mandibles for large-sized ungulates or cranial remains with maxillae for smaller ones (muntjacs), or complete jaws in some cases (wild pigs) (Bacon et al., 2015). Factors involved in the selection of remains by porcupines might be the weight, size and density of elements. This capacity of porcupines to collect a wide range of animals means that assemblages are representative of the species diversity of large mammals. Furthermore, as previously noticed by Brain (1981) for African sites, "*It is evident that the minimum numbers of individual animal represented by the porcupine collected remains do indeed mirror the actual abundance of the antelope species*", it can be reasonably proposed that assemblages are also representative of the abundance of species at a local scale.

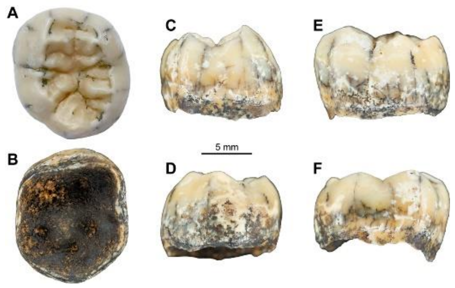

A. Different view of the first or second molar of a juvenile Denisovan female individual (TNH2-1) from Cobra Cave (164-131 ka) (Demeter et al., 2022). Source: Authors.

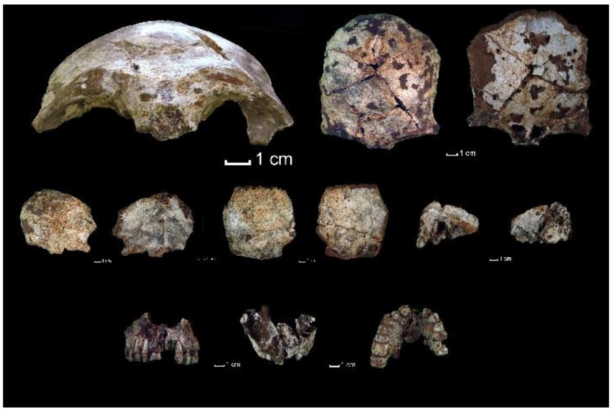

B. Elements of the partial skull of the TPL-1 *Homo sapiens* individual from Tam Pà Ling Cave (46-43 ka) (Demeter et al., 2012). We used the published isotopic data of the upper first molar (Bourgon et al., 2021). Source: Authors.

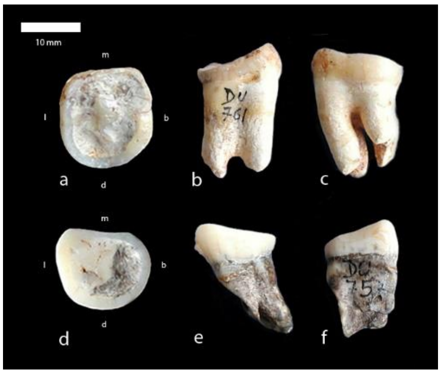

C. Isolated hominin teeth (*Homo* sp.) from Duoi U'Oi site (70-60 ka) (Bacon et al., 2018). Right lower m1 (DU761) in occlusal (A), buccal (B), and lingual (C) views; Right upper M3 (DU757) in occlusal (D), buccal (E), and lingual views (F). Source: Authors.

Figure S2. Fossil hominin remains used in the present analysis. Source: Authors.

Conservation of the Tam Pà Ling assemblage and environmental consideration: Unlike the faunal assemblages cited above, the Tam Pà Ling faunal assemblage accumulated under a different sedimentary process. As described in Demeter et al. (2012) "*Sediments in the stratigraphic sequence of the excavation form a series of intercalated, clay-rich slopewash deposits that originated outside the south-facing entrance and were carried into the cave".* As a result, the site yielded abundant microvertebrates (rodents, insectivores, reptiles, and amphibians), but only 28 specimens of large mammals (Caprinae, Cervidae, *Hystrix* sp., *Muntiacus* sp., *Macaca* sp., *Rhinoceros* cf. *unicornis*, *Dicerorhinus sumatrensis*) over the ~7 meters high stratigraphy.

For the purpose of our study, we selected the carbon values of Artiodactyla and Perissodactyla specimens previously published, and recovered in the sedimentary section between 70 and 33 ka (Bourgon et al., 2021) (Annex S2). The selection of this time range is based on the isotopic composition of *Camaena massiei* shells recorded over the TPL stratigraphy which documents major changes ~33 ka in the proportions of C_3 and C_4 plants, due to the settlement of the Last Glacial Maximum conditions (Milano et al., 2018) (Fig. S3).

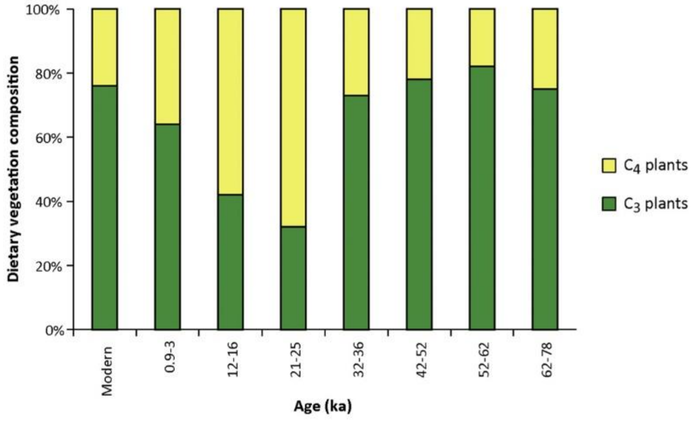

Figure S3. Vegetation composition as C_3 and C_4 plants proportions derived from the isotopic *Camaena massiei* shells recorded at Tam Pà Ling (Milano et al., 2018). Source: Authors.

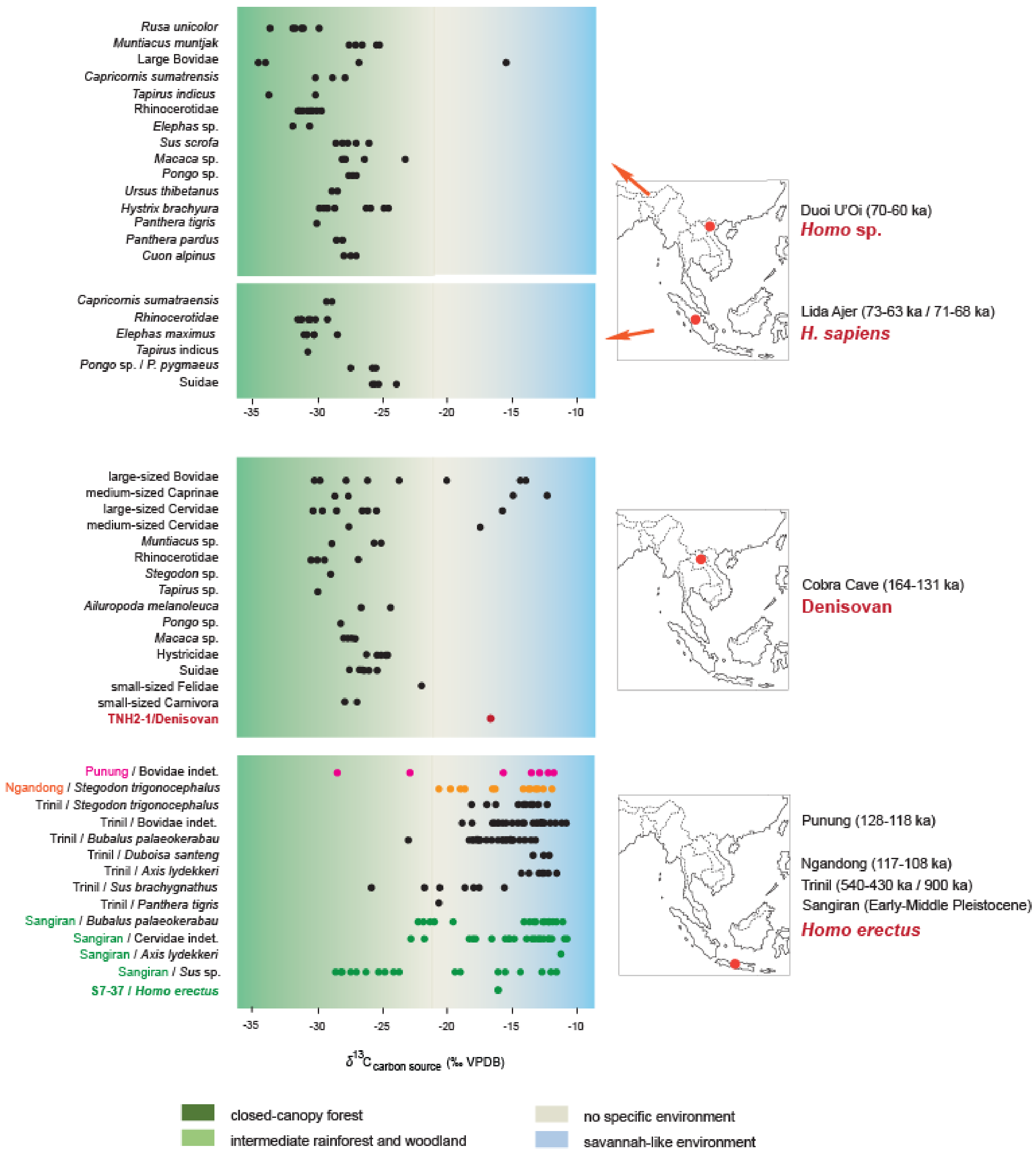

Figure S4. Distribution of available published $\delta^{13}C_{carbon\ source}$ values of mammals from different hominin sites: Duoi U'Oi (*Homo* sp.), northern Vietnam (Bacon et al., 2021) dated to 70–60 ka (Bacon et al., 2008, 2015); Lida Ajer (*Homo sapiens*), Sumatra (Louys et al., 2022) dated to 73–63 ka (Westaway et al., 2017) or 71-68 ka (Louys et al., 2022); Trinil H.K. and Sangiran (*Homo erectus*), Java (Janssen et al., 2016) dated to 540–430 ka (Joorden et al., 2015) or to ~900 ka (Basal Layer of the Bapang Formation from Matsu'ura et al., 2020, Fig. 1C); *Homo erectus* specimen from Sangiran (S7–37) (Kubat et al., 2023); Ngandong (*Homo erectus*), Java (Janssen et al., 2016) dated to 117–108 ka (Rizal et al., 2020); Tam Ngu Hao 2 (Cobra) Cave (Denisovan), northeastern Laos (this paper) dated to 164–131 ka (Demeter et al., 2022). The presence of hominins (*Homo sapiens*) in the Punung site, Java (Storm et al., 2005) dated to 128–118 ka ((Westaway et al., 2017) is debated (Kaifu et al., 2022). All isotopic data of specimens can be found in the Annexes. Source: Authors.

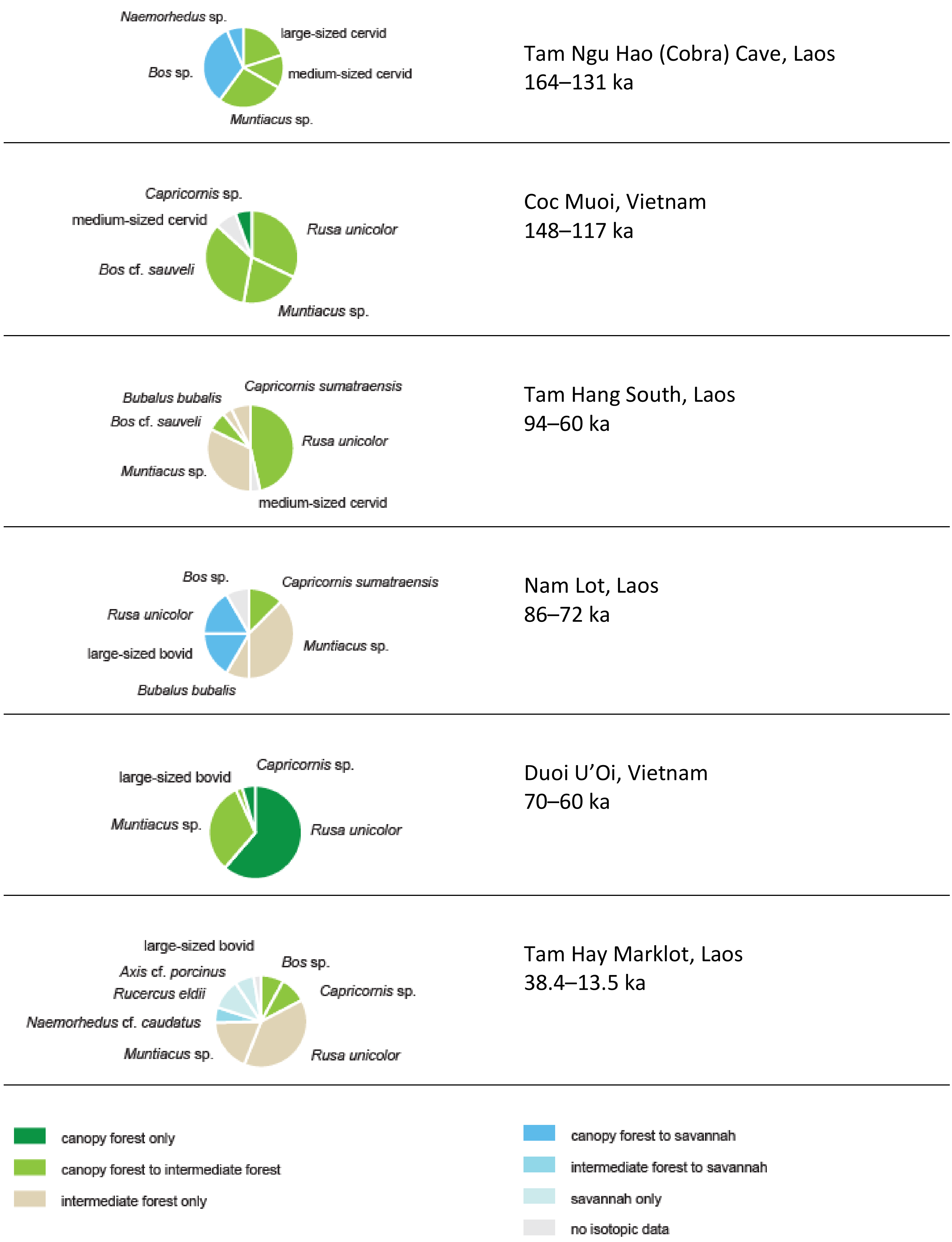

Figure S5. Species abundance calculated using MNI within Bovidae and Cervidae expressed as percentages of taxa in each faunal assemblage (see Table S12 for the data). Source: Silhouette image from public domain https://www.phylopic.org; charts, Authors.

Country	Site	Age interval	Dating technique (sediments)	Dating technique (remains)
Laos	Tam Hang South	94 – 60 ka	U-series, SG-OSL, TL[1]	-
Laos	Nam Lot I Cave	86 – 72 ka	U-series, SG-OSL, TL[1]	-
Laos	Tam Ngu Hao 2 (Cobra Cave)	164 – 131 ka	U-series, pIR-IRSL[2]	U-series/ESR (teeth)
Laos	Tam Hay Marklot	34.5 – 13.5 ka	-	U-series/ESR (teeth)[3]
Laos	Tam Pà Ling	46 – 43 ka	SG-OSL, pIR-IRSL[4, 5]	-
Vietnam	Coc Muoi Cave	148 – 117 ka	U-series, SG-OSL, pIR-IRSL[6]	-
Vietnam	Duoi U'Oi Cave	70 – 60 ka	U-series, SG-OSL, TL[1]	AMSC^{14} (teeth), >32 cal kBP[7, 8]
Russia	Denisova Cave (MC, 17-19)	151 ± 17-128 ± 13 ka	pIR-IRSL[9]	-

Table S1: Dating techniques and materials used to constrain the ages of faunas and associated hominins. [U-series] Uranium-series; [TL] red thermoluminescence; [SG-OSL] single-grain optically stimulated luminescence, [pIR-IRSL] post-infrared infrared-stimulated luminescence; [ESR] electron spin resonance; [AMSC^{14}] radiocarbon dating by accelerator mass spectrometry. [(1)] Bacon et al., 2015; [(2)] Demeter et al., 2022; [(3)] Bourgon et al., 2020; [(4)] Demeter et al., 2012; [(5)] Shackelford et al., 2018; [(6)] Bacon et al., 2018; [(7)] Wood et al., 2016; [(8)] Wood et al., 2021. [(9)] The occupation of the cave by Denisovans is estimated using the shorter chronology between 203 ± 14 ka and 55 ± 6 ka (Jacobs et al., 2019). For the purpose of our comparative analysis between Cobra Cave (164–131 ka) and Denisova Cave, we used data (fauna and flora) of the Main Chamber (MC), layers 19–17, dated between 151 ± 17–128 ± 13 ka.

Taxon 1	Common name	Taxon 2	Cobra Cave	Coc Muoi	Tam Hang South	Nam Lot I	Duoi U'Oi	Tam Hai Marklot
Large-sized Cervidae	Large deer	large-sized cervid	X					
Large-sized Cervidae	Sambar	*Rusa unicolor*		X	X	X	X	X
Medium-sized Cervidae	-	medium-sized cervid	X	X	X			
Medium-sized Cervidae	Thamin	*Rucervus eldii*						X
Medium-sized Cervidae	Hog deer	*Axis porcinus*						cf.
Small-sized Cervidae	Indian muntjac	*Muntiacus muntjak*			X	X	X	
Small-sized Cervidae	-	*Muntiacus* sp.	X	X				X
Large-sized Bovidae	Kouprey	*Bos sauveli*		cf.	cf.			
Large-sized Bovidae	Gayal	*Bos frontalis*						cf.
Large-sized Bovidae	-	*Bos* sp.	X			X		
Large-sized Bovidae	Large-sized Bovidae	Bovidae indet.		X	X	X	X	X
Large-sized Bovidae	Water buffalo	*Bubalus bubalis*			X	X		X
Large-sized Caprinae	Southern serow	*Capricornis sumatraensis*			X	X	X	cf.
Large-sized Caprinae	-	*Capricornis* sp.		X				
Medium-sized Caprinae	Chinese goral	*Naemorhedus caudatus*						cf.
Medium-sized Caprinae	-	*Naemorhedus* sp.	X					
Suidae	Wild boar	*Sus scrofa*	X	X	X	X	X	X
Suidae	Bearded pig	*Sus barbatus*			cf.		X	cf.
Megatapirus augustus	Giant tapir	*Megatapirus augustus*		X	X			
Tapirus sp.	-	*Tapirus indicus intermedius*			cf.			
Tapirus sp.	Malayan tapir	*Tapirus indicus*		X			X	
Tapirus sp.	-	*Tapirus* sp.	X			X		X

		Tapiridae indet.		X				X
Rhinocerotidae	Indian rhinoceros	*Rhinoceros unicornis*		cf.	X	X	X	
Rhinocerotidae	Javan rhinoceros	*Rhinoceros sondaicus*	X	X	X	X	X	X
Rhinocerotidae		*Rhinoceros* sp.	X			X	X	
Rhinocerotidae	Sumatran rhinoceros	*Dicerorhinus sumatrensis*		X			X	X
Rhinocerotidae	-	*Dicerorhinus* sp.	X		X			
Rhinocerotidae		Rhinocerotina indet.	X				X	X
Rhinocerotidae		Rhinocerotidae indet.		X		X		
Elephas sp.	Asian elephant	*Elephas maximus*		X				
Elephas sp.	-	*Elephas* sp.			X	X	X	
Stegodon sp.	Stegodon	*Stegodon orientalis*			X	cf.		
Stegodon sp.	-	*Stegodon* sp.	X	X				

Table S2: Faunal lists of studied sites (Artiodactyla, Perissodactyla, and Proboscidea). Taxon 1 refers to the category used in the presentation of isotopes results, and Taxon 2 refers to the precise assignment of specimens.

**Taxon 1**	**Common name**	**Taxon 2**	**Cobra Cave**	**Coc Muoi**	**Tam Hang South**	**Nam Lot I**	**Duoi U'Oi**	**Tam Hai Marklot**
	Carnivoran	Small-sized carnivora	X					?
Canidae	Dhole	*Cuon alpinus*		X		X	X	
Canidae	Dhole	*Cuon alpinus antiquus*			cf.			
Canidae	Wild dogs	Canidae						X
	Hog-badger	*Arctonyx collaris*					X	
	Hog-badger	*Arctonyx collaris rostratus*			cf.			
	Eurasian badger	*Meles meles*			X	X	X	
	Large tooth ferret-badger	*Melogale personata*			X			
	Yellow-throated marten	*Martes flavigula*			cf.	X		
	-	*Martes* sp.		X				
	Large Indian civet	*Viverra zibetha*			X	X	X	
	Large-spotted civet	*Viverra megaspila*					cf.	
		Viverridae indet.					X	
	Common palm civet	*Paradoxurus hermaphroditus*			X			
	-	*Paradoxurus* sp.	X	X				
	-	large-sized meline		X				
	-	small-sized meline		X				X
Panthera tigris	Tiger	*Panthera tigris*		X	X		X	X
Panthera pardus	Leopard	*Panthera pardus*					X	X
Small-sized Felidae	Leopard cat	*Prionailurus bengalensis*			cf.			
Small-sized Felidae	Golden cat	*Felis temmincki*				cf.		
Small-sized Felidae	Clouded leopard	*Neofelis nebulosa*				X	X	
Small-sized Felidae	-	small-sized felid	X	X				X
	Spotted hyena	*Crocuta crocuta*				X		
Ursidae	Asiatic black bear	*Ursus thibetanus*	X	X			X	X
Ursidae	Asiatic black bear	*Ursus thibetanus kokeni*			cf.	cf.		
Ursidae	Sun bear	*Helarctos malayanus*		cf.	X		X	X
Ursidae		*Ursus* sp.		X				
	Giant Panda	*Ailuropoda melanoleuca*		X		X		X
	Giant Panda	*Ailuropoda* sp.	X					
Pongo sp.	Orangutan	*Pongo pygmaeus*			X	X	X	

Pongo sp.	Orangutan	*Pongo devosi*		X				
Pongo sp.	-	*Pongo* sp.	X					X
Macaca sp.	Macaque	*Macaca* sp.		X		X	X	X
Macaca sp.		*Macaca nemestrina*	cf.					
Colobinae	-	Colobinae indet.		X	X	X	X	
	Gibbon	*Hylobates* sp.		X	X		X	
Homininae		Hominine	X				X	
Hystricidae	Porcupine	*Hystrix brachyura*			X	X	X	
Hystricidae	-	*Hystrix* sp.	X	X				X
Hystricidae	Brush-tailed porcupine	*Atherurus macrourus*						cf.
Hystricidae	-	*Atherurus* sp.		X				

Table S3: Faunal lists of the five sites (Carnivora, Primates, and large Rodentia). Taxon 1 refers to the category used in the presentation of isotopes results, and Taxon 2 refers to the precise assignment of specimens.

	**Artiodactyla**		**Perissodactyla**		**Proboscidea**		**Carnivora**		**Primates**		**Rodentia**	
**Cobra cave**	59.25 %	32/54	9.25 %	5/54	1.85 %	1/54	9.25 %	5/54	11.11 %	6/54	9.25 %	5/54
**Coc Muoi**	35.71 %	30/84	20.23 %	17/84	4.76 %	4/84	16.66 %	14/84	10.71 %	9/84	11.90 %	10/84
**Tam Hang South**	56.45 %	35/62	9.67 %	6/62	-	-	14.51 %	9/62	9.67 %	6/62	9.67 %	6/62
**Nam Lot I**	35.08 %	20/57	15.78 %	9/57	5.26 %	3/57	26.31 %	15/57*	10.52 %	6/57	7.01 %	4/57
**Duoi U'Oi**	38.33 %	23/60	18.33 %	11/60	3.33 %	2/60	13.33 %	8/60	13.33 %	8/60	13.33 %	8/60
**Tam Hay Marklot**	54.16 %	39/72	9.72 %	7/72	-	-	20.83 %	15/72	9.72 %	7/72	5.55 %	4/72

Table S4: Percentage (%) and number of specimens (n/N) by taxonomic group used for the carbon and oxygen isotope analyses. The data of Cobra Cave (Primates) includes the Denisovan tooth. (*) The sample includes 5 teeth of Nam Lot II, another locality in the cave, which produced an assemblage of comparable composition to that Nam Lot I.

	**$\delta^{13}C_{carbon\ source}$ < –27.2 ‰**		**$\delta^{13}C_{carbon\ source}$ >–27.2 ‰ and < –21.3 ‰**		**$\delta^{13}C_{carbon\ source}$ > –21.3 ‰ and < –15.3 ‰**		**$\delta^{13}C_{carbon\ source}$ > –15.3‰**	
**Cobra Cave**	37 %	20/54	48 %	26/54	7 %	5/54	7 %	4/54
**Coc Muoi**	65.48 %	55/84	33.33 %	28/84	1.19 %	1/84	-	0/84
**Tam Hang South**	27.42 %	17/62	58.06 %	36/62	11.29 %	7/62	3.23 %	2/62
**Nam Lot I**	42.11 %	21/57	49.12 %	28/57	5.76 %	3/57	3.84 %	2/57
**Duoi U'Oi**	73.33 %	44/60	25 %	15/60	-	0/60	1.67 %	1/60
**Tam Hay Marklot**	26.39 %	19/72	43.06 %	31/72	18.05 %	13/72	12.50 %	9/72

Table S5: Percentage (%) and number of specimens (n/N) in the five faunas according to the distribution of $\delta^{13}C_{carbon\ source}$ values (‰ VPDB). The data of Cobra Cave include the Denisovan tooth.

Group1	Group2	N (group1)	N (group2)	statistic	p	p.adj
Cobra	Coc Muoi	53	84	-4.53	0.000	0.000
Cobra	Tam Hang South	53	62	0.82	0.410	0.44
Cobra	Nam Lot I	53	57	-0.815	0.414	0.44
Cobra	Duoi U'Oi	53	60	-4.656	0.000	0.000
Cobra	Tam Hay Marklot	53	72	1.913	0.055	0.08
Coc Muoi	Tam Hang	84	62	5.676	0.000	0.000
Coc Muoi	Nam Lot I	84	57	3.733	0.000	0.00
Coc Muoi	Duoi U'Oi	84	60	-0.481	0.630	0.63
Coc Muoi	Tam Hay Marklot	84	72	7.114	0.000	0.000
Tam Hang South	Nam Lot I	62	57	-1.687	0.091	0.12
Tam Hang South	Duoi U'Oi	62	60	-5.697	0.000	0.000
Tam Hang South	Tam Hay Marklot	62	72	1.109	0.267	0.33
Nam Lot I	Duoi U'Oi	57	60	-3.904	0.000	0.000
Nam Lot I	Tam Hay Marklot	57	72	2.830	0.004	0.01
Duoi U'Oi	Tam Hay Marklot	60	72	7.002	0.000	0.000

Table S6: Results of the Post-hoc Dunn's test pair-wise comparisons on $\delta^{13}C_{carbon\ source}$ values between sites.

Group1	Group2	N (group1)	N (group2)	statistic	p	p.adj
Cobra	Coc Muoi	53	84	2.158	0.030	0.06
Cobra	Tam Hang South	53	62	-0.445	0.656	0.76
Cobra	Nam Lot I	53	57	3.208	0.001	0.01
Cobra	Duoi U'Oi	53	60	0.265	0.790	0.80
Cobra	Tam Hay Marklot	53	72	3.133	0.001	0.01
Coc Muoi	Tam Hang South	84	62	-2.758	0.005	0.01
Coc Muoi	Nam Lot I	84	57	1.361	0.173	0.26
Coc Muoi	Duoi U'Oi	84	60	-1.943	0.051	0.09
Coc Muoi	Tam Hay Marklot	84	72	1.173	0.240	0.33
Tam Hang South	Nam Lot I	62	57	3.790	0.000	0.00
Tam Hang South	Duoi U'Oi	62	60	0.736	0.461	0.58
Tam Hang South	Tam Hay Marklot	62	72	3.753	0.000	0.00
Nam Lot I	Duoi U'Oi	57	60	-3.039	0.002	0.01
Nam Lot I	Tam Hay Marklot	57	72	-0.255	0.798	0.80
Duoi U'Oi	Tam Hay Marklot	60	72	2.957	0.003	0.01

Table S7: Results of the Post-hoc Dunn's test pair-wise comparisons on $\delta^{18}O$ values between sites.

Habitat	Altitude range	Body weigth	Common name	Family	Taxon	Cobra Cave (164-131 ka)	Coc Muoi (148-117 ka)	Indochina	Baishiya Cave (~100 ka)	Tibetan Plateu	Denisova Cave (Layer 17)	Denisova Cave (Layer 19)	Siberia
Open habitat to dense forest	Up to 2,100m (Eurasia)<br>Up to 3,000m (Indochina)	10-20 kg	Dhole[1]	Canidae	*Cuon alpinus*		X	X		X		X	
Artic and alpine tundras	Low to high mountains	1.4-9 kg	Artic fox[3]	Canidae	*Alopex lagopus*							X	X
Open steppe and semidesert	Lowland	1.8-2.8 kg	Corsac fox[1]	Canidae	*Vulpes corsac*					X		X	X
Semidesert, forest, high mountain toundra	High mountain	3.6-7 kg	Red fox[1]	Canidae	*Vulpes vulpes*					X	X	X	X
Tundra, forest, plain, desert, alpine zone	High mountain	28-40 kg	Wolf[1]	Canidae	*Canis lupus*					X	X	X	X
Forest, subalpine mountain, tundra	High mountain	125-225 kg	Brown or grizzly bear[1]	Ursidae	*Ursus arctos*					X		X	X
Mixed forest, oak forest, tropical forest	Up to 3,600 m	54-240 kg	Asiatic black bear[1]	Ursidae	*Ursus thibetanus*	X	X	X		X			
Tropical forest	Lowland	27-63 kg	Sun bear[2]	Ursidae	*Helarctos mayalanus*		cf.	X					
Mixed coniferous and broadleaf forest	1,200-3,900 m	85-125 kg	Giant Panda[1]	Ursidae	*Ailuropoda* sp./*A. melanoleuca*	X	X			X			
Coniferous forest, open rocky zone	1,300-3,000 m	1-3 kg	Marte[2]	Mustelidae	*Martes* sp.		X	X					
Coniferous and deciduous forests	Low to high mountains	0.4-1.1 kg	Sable[1]	Mustelidae	*Martes zibellina*							X	X
Open steppe	High mountain	460-1,198 gr	Steppe polecat[1]	Mustelidae	*Mustela eversmannii*					X		X	X
Mixed forest, tundra, woodland	2,000-3,000 m	60-110 gr	Ermine[1]	Mustelidae	*Mustela erminea*							X	X
Forest, steppe, meadow, mountain	Up to 4,000 m	28-70 gr	Least weasel[1]	Mustelidae	*Mustela nivalis*					X		X	X
			Cave hyena	Hyaenidae	*Crocuta spelea*				?		X	X	
Dense forest, steppe, alpine region	High mountain	18-38 kg	Lynx[1]	Felidae	*Lynx lynx*					X		X	X
Scrub forest<br>Tropical rainforest	Up to 3,900 m (China)	100-306 kg[1]<br>100-258 kg[1]	Tiger (Siberia)[1]<br>Tiger (India)[1]	Felidae	*Panthera tigris*		X	X		X			X
-	-	-	-	Felidae	small-sized felid	X	X	X		X			X
Mountain, subtropical, tropical forests	Lowland	2.4-4 kg[1]	Palm civet[1,2]	Viverridae	*Paradoxurus* sp.	X	X	X					
Tropical/subtropical forest, woodland	Up to 3,700 m	185-260 kg[1]	Sambar deer[1]	Cervidae	*Rusa unicolor*	X	X	X	?	X			
-	-	-	-	Cervidae	medium-sized cervid	X	X	X	?				
Tropical forest, mountain forest	Lowland to high mountain	~15-up to 40kg	Muntjac[1,2]	Cervidae	*Muntiacus* sp.	X	X	X	?	X			
Woodland, open meadow	Lowland to high mountain	20-40 kg[1]	Siberian roe[1]	Cervidae	*Capreolus pygargus*				?	X	X	X	X
Conifer forest, open alpine meadow	Up to 5,000 m	75-240 kg[1]	Red deer[1]	Cervidae	*Cervus elaphus*				?	X	X	X	X
Dense to open tropical/subtropical forest	Lowland/medium mountain	650-1,500 kg[1]<br>650-900 kg[2]	Gaur[1]	Bovidae	*Bos* sp. (*frontalis*)	X		X	?				
Mixed savannah, open grassland	Lowland	700-900 kg[2]	kouprey	Bovidae	*Bos* sp. (*sauveli*)		cf.	X	?				
Desert, grassland	4,000-6,100m	306-821 kg[1]	Yak[1]	Bovidae	*Poephagus* (*Bos grunniens*?)				?	X		?	
Steppe	Lowland to High mountain	1,100 kg	Steppe bison	Bovidae	*Bison priscus*				?		X	X	
Grassy steppe, semidesert, grassland	Up to 5,750 m	25-45 kg[1]	Mongolian gazelle[1]	Bovidae	*Procapra gutturosa*				?	X	X	X	X
Grassy plain, aride zone	Lowland	26-69 kg[1]	Steppe saiga[1]	Bovidae	*Saiga tatarica*				?			X	X

Mountain, rocky terrain, open meadow	3,000-6,000 m	80-100 kg[(1)]	Siberian ibex[(1)]	Bovidae	*Capra sibrica*				?		X	X	X
Alpine grassland	3,000-5,000 m	95-140 kg[(1)]	Argali[(1)]	Bovidae	*Ovis ammon*				?	X	X	X	X
Forest, meadow, rocky terrain	Lowland up to 4,500 m	~20-42 kg[(1, 2)]	Goral[(1, 2)]	Bovidae	*Naemorhedus* sp. (*N. griseus, N. caudatus*)	X		X	?	X			X
Forest, rocky zone<br>Dense subtropical/tropical forest	Lowland up to 4,500 m<br>Lowland to medium moutain	85-140 kg[(1)]<br>85-140 kg[(2)]	Chinese serow[(1)]<br>Southern serow[(2)]	Bovidae	*Capricornis* sp. (*C. milneedwardsii*)<br>*Capricornis* sp. (*C. sumatraensis*)		X	X<br>X	?	X			
Forest, scrub, grassland, swamp	Lowland to high mountain	50-200 kg[(1, 2)]	Wild boar[(1, 2)]	Suidae	*Sus scrofa*	X	X	X		X			X
Tropical rainforest	Lowland to high mountain	>800 kg[(4)]	Giant tapir	Tapiridae	*Megatapirus augustus*		X						
Tropical rainforest	Lowland up to 2,000 m	250-350 kg[(2)]	Malayan tapir[(2)]	Tapiridae	*Tapirus* sp./*T. indicus*	X	X	X					
Tropical rainforest	Lowland	1,500-2,000 kg	Javan rhinoceros[(2)]	Rhinocerotidae	*Rhinoceros sondaicus*	X	X	X	?				
Savannah, grassland, scrub forest [(7)]	Lowland	1,800-2,700 kg	Indian rhinoceros	Rhinocerotidae	*Rhinoceros unicornis*		cf.		?				
Tropical rainforest	Lowland	900-1,000 kg[(2)]	Sumatran rhinoceros[(2)]	Rhinocerotidae	*Dicerorhinus* sp./*D. sumatrensis*	X	X	X	?				
Steppe, harsh conditions	Lowland to high mountain?	3,000 kg	Woolly rhinoceros	Rhinocerotidae	*Coelodonta antiquitatis*				?		X	X	
Steppe, grassland	Lowland to high mountain?	140-230 kg	European wild ass	Equidae	*Equus hydruntinus*				?			X	
Steppe, grassland, semi-arid area	Lowland to high mountain?	200-300 kg	Wild horse	Equidae	*Equus ferus*				?			X	
Tropical forest	Lowland	3,000-5,000 kg[(5)]	Stegodon	Stegodontidae	*Stegodon* sp.	X	X						
Steppe	Lowland	5,500-7,300 kg	Woolly mammoth	Elephantidae	*Mammuthus primigenius*							X	
Tropical rainforest	Lowland	3,000-5,000 kg[(2)]	Asian elephant[(2)]	Elephantidae	*Elephas maximus*		X	X					

Table S8: Taxa recorded at the genus or species level in the Cobra Cave (164-131 ka; Demeter et al., 2022), Coc Muoi Cave (148-117 ka; Bacon et al., 2018), Denisova Cave (layers 17-19, Main Chamber, 151 ± 17 – 128 ± 13 ka; Jacobs et al., 2019), and Baishiya Cave (layers 10-6, ~100 ka; Figs. S27; Zhang et al., 2020), and in current faunas from Indochinese, Siberian and Tibetan Plateau zones. Also noted are habitat type, altitudinal range, and body weight for each taxon. The presence of brown bear (*Ursus arctos*) and that of east Asian cave hyena (*Crocuta spelea*, Haplogroup D) in the layer 19 of the Main Chamber of the Denisova Cave has also been reported by Zavala et al., (2021) based on sedimentary DNA. [(1)] Smith A.T., & Xie Y. (2008), [(2)] Francis C.M. (2008), [(3)] Nowak R.M. (1999), [(4)] Janis (1984), [(5)] Schepartz and Miller-Antonio (2010).

Habitat	Altitude range	Diet		BW	Common name	Taxon	Cobra Cave (164-131 ka)	Coc Muoi (148-117 ka)	Indochina	Bayshia Cave (~100 ka)	Tibetan Plateu	Denisova Cave (Layer 17)	Denisova Cave (Layer 19)	Siberia
Tropical/subtropical forest, woodland	Up to 3,700 m	Grass, browse, ferns, leaves[1,2]	Browser/mixed feeder[6]	B	Sambar deer [1]	*Rusa unicolor*	X	X	X	?	X			
-	-	-	Mixed feeder/grazer[6]	A/B	-	medium-sized cervid	X	X	X	?				
Tropical forest, mountain forest	Lowland to high mountain	Grass, leaves, herbs, fruits[1,2]	Browser	A	Muntjac [1 2]	*Muntiacus* sp.	X	X	X	?	X			
Woodland, open meadow	Lowland to high mountain	Grass, browse, bark[1]	Browser/mixed feeder	A	Siberian roe [1]	*Capreolus pygargus*				?	X	X	X	X
Conifer forest, open alpine meadow	Up to 5,000 m	Lichens>grass, herbs, mosses, bark[1]	Browser/mixed feeder	B	Red deer [1]	*Cervus elaphus*				?	X	X	X	X
Dense to open tropical/subtropical forest	Lowland to medium mountain	Grass, browse, bamboo shoots[1,2]	Browser/mixed feeder/grazer[6]	C/D	Gaur [1]	*Bos* sp. (*frontalis*)	X		X					
Mixed savannah, grassland, forest	Lowland	Leaves, roots, tubers[7]	Browser/mixed feeder/grazer[6]	C	kouprey	*Bos* sp. (*sauveli*)		cf.	X					
Desert, grassland	4,000-6,100 m	Grass[1]	Grazer	C	Yak [1]	*Poephagus* (*Bos grunniens*?)				?	X		?	
Steppe	Lowland to High mountain	Grass, sedge, herb[8,9,10]	Grazer	D	Steppe bison	*Bison priscus*						X	X	
Grassy steppe, semidesert, grassland	Up to 5,750 m	Grass, some browse[1]	Grazer	A	Mongolian gazelle [1]	*Procapra gutturosa*				?	X	X	X	X
Grassy plain, aride zone	Lowland	Grass, some browse[1]	Grazer	A	Steppe saiga [1]	*Saiga tatarica*				?			X	X
Mountain, rocky terrain, open meadow	3,000-6,000 m	Grass, herb[1]	Grazer	B	Siberian ibex [1]	*Capra sibrica*				?		X	X	X
Alpine grassland	3,000-5,000 m	Grass, herb[1]	Grazer	B	Argali [1]	*Ovis ammon*				?	X	X	X	X
Forest, meadow, rocky terrain	Lowland up to 4,500 m	Grass, herb, twigs[1,2]	Browser/mixed feeder	A	Goral[1,2]	*Naemorhedus* sp. (*N. griseus, N. caudatus*)	X		X	?	X			X
Forest, rocky zone<br>Dense subtropical/tropical forest	Up to 4,500 m<br>Lowland/medium moutain	Leaves, shoots[1,2]	Browser/mixed feeder	B<br>B	Chinse Serow[1]<br>Southern serow[2]	*Capricornis* sp. (*C. milneedwardsii*)<br>*Capricornis* sp. (*C. sumatraensis*)		X	X<br>X	?	X			
Forest, scrub, grassland, swamp	Lowland to high mountain	Roots, seeds, eggs, animals[1,2]	Browser/mixed feeder	B	Wild boar[1,2]	*Sus scrofa*	X	X	X		X			X
Tropical rainforest	Lowland to high mountain	Grass, leaves, twigs, bark, herb, fruits	Browser[6]	C	Giant tapir	*Megatapirus augustus*		X	X					
Tropical rainforest	Lowland up to 2,000 m	Grass, leaves, twigs, bark, herb, fruits, aquatic plants[7]	Browser[6]	B	Malayan tapir[2]	*Tapirus* sp./*T. indicus*	X	X	X					

Tropical rainforest	Lowland [2]	Leaves, wood, bark, fruits[7]	Browser[6]	D	Javan rhinoceros[2]	*Rhinoceros sondaicus*	X	X	X	?				
Savannah, grassland, scrub forest [7]	Lowland [7]	Leaves, wood, bark, fruit, aquatic plants[7]	Browser[6]	D	Indian rhinoceros	*Rhinoceros unicornis*		cf.		?				
Tropical rainforest	Lowland	Leaves, twigs, woody plants[2]	Browser[6]	C	Sumatran rhinoceros[2]	*Dicerorhinus* sp./*D. sumatrensis*	X	X	X	?				
Steppe, harsh conditions	Lowland	Grass, sedge[8]	Grazer	D	Woolly rhinoceros	*Coelodonta antiquitatis*				?		X	X	
Steppe, grassland	Lowland	Grass, sedge = leaves of shrubs and trees[8,9, 10]	Grazer/mixed feeder	B	European wild ass	*Equus hydruntinus*				?			X	
Steppe, grassland, semi-arid area	Lowland	Grass, sedge = leaves of shrubs and trees[8,9, 10]	Grazer/mixed feeder	B	Wild horse	*Equus ferus*				?			X	
Tropical forest	Lowland	Plants, bark, fruits	Browser[6]	D	Stegodon	*Stegodon* sp.	X	X						
Steppe	Lowland	Mature dry grass [9], sedge[8, 9]	Grazer	D	Woolly mammoth	*Mammuthus primigenius*							X	
Tropical rainforest	Lowland	Grass, plants, bark, fruits[2]	Browser[6]	D	Asian elephant[2]	*Elephas maximus*		X	X					

Table S9: Taxa recorded at the genus or species level in the Cobra Cave (164-131 ka; Demeter et al., 2022), Coc Muoi Cave (148-117 ka; Bacon et al., 2108), Denisova Cave (layers 17-19, Main Chamber, 151 ± 17 – 128 ± 13 ka; Jacobs et al., 2019), and and Baishiya Cave (layers 10-6, ~100 ka; Figs. S27; Zhang et al., 2020), and in current faunas from Indochinese, Siberian and Tibetan Plateau zones. Also noted are habitat type, altitudinal range, diet and dietary strategy, and body weight category for each taxon. [1] Smith A.T., & Xie Y. (2008), [2] Francis C.M. (2008), [3] Nowak R.M. (1999), [4] Janis (1984), [5] Schepartz and Miller-Antonio (2010), [6] data based on isotopic measurements (C13) from Late Pleistocene Indochinese sites (Bacon et al., 2015), [7] https://animaldiversity.org, [8] Ma et al., (2021), [9] Drucker (2022), [10] Kelly et al., (2021). Body size categories from Faith et al., (2019); A. 18-80 kg, B. 80-350 kg, C. 350-1000 kg, D. >1000 kg.

		**Cobra Cave**	**Denisova Cave Main Chamber (layer 17)**	**Denisova Cave East Chamber (layer 19)**
		**% (NISP)**	**% (NISP)**	**% (NISP)**
**Artiodactyla**	Cervidae	29.11	16.66	1.77
	Bovidae	28.48	29.16	22.49
	Suidae	24.05	-	-
		**81.64**	**45.82**	**24.26**
**Perissodactyla**	Rhinocerotidae	11.39	8.33	3.55
	Equidae	-	12.50	10.07
	Tapiridae	0.63	-	-
		**12.02**	**20.83**	**13.62**
**Proboscidea**	Stegodontidae	0.63	-	-
	Mammuthus	-	-	2.37
		**0.63**	**-**	**2.37**
**Carnivora**	Felidae	0.63	-	1.18
	Hyenidae	-	8.33	16.57
	Ursidae	3.16	16.67	9.46
	Canidae	-	8.34	29.54
	Others	1.89	-	2.95
		**5.68**	**33.34**	**59.70**

Table S10: Percentages of fossil specimens recovered in the Cobra Cave (isolated teeth) (Demeter et al., 2022) and layers 17 and 19 of the Main Chamber in the Denisova Cave (bones) (Jacobs et al., 2019) by Family. Number of identified specimens (NISP).

		A (18 – 80 kg)	B (80 – 350 kg)	C (350 – 1000 kg)	D (>1000 kg)	Distribution of taxa
**Tam Ngu Hao 2 (Cobra) Cave (164 – 131 ka)**<br>**Denisovans**	**Ruminant**	small-sized cervid<br>*Naemorhedus* sp.	large-sized cervid<br>medium-sized cervid	*Bos* sp.		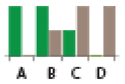
	**Non-ruminant**		*Sus scrofa*	*Tapirus* sp.<br>*Dicerorhinus* sp.	*Rhinoceros sondaicus*<br>*Stegodon* sp.	
**Coc Muoi**<br>**(148 – 117 ka)**	**Ruminant**	*Muntiacus* sp.	*Rusa unicolor*<br>medium-sized cervid<br>*Capricornis* sp.	*Bos* cf. *sauveli*		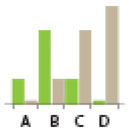
	**Non-ruminant**		*Sus scrofa*	*Megatapirus augustus*<br>*Tapirus indicus*<br>*Dicerorhinus sumatrensis*	*Rhinoceros sondaicus*<br>*Rhinoceros* cf. *unicornis*<br>*Elephas maximus*<br>*Stegodon* sp.	
**Denisova Cave**<br>**(128 ± 13 – 151 ± 17 ka)**<br>**Denisovans**	**Ruminant**	*Capreolus pygargus*<br>*Procapra gutturosa*<br>*Saiga tatarica*	*Cervus elaphus*<br>*Capra sibrica*<br>*Ovis ammon*	*Poephagus/Bison?*	*Bison priscus*	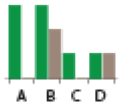
	**Non-ruminant**		*Equus hydruntinus*<br>*Equus ferus*		*Coelodonta antiquitatis*	
**Tam Hang South**<br>**(92 – 60 ka)**	**Ruminant**	*Muntiacus muntjak*	*Rusa unicolor*<br>medium-sized cervid<br>*Capricornis sumatraensis*	*Bos* cf. *sauveli*<br>*Bubalus bubalis*		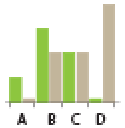
	**Non-ruminant**		*Sus scrofa*<br>*Sus* cf. *barbatus*	*Megatapirus augustus*<br>*Tapirus indicus* cf. *intermedius*	*Rhinoceros sondaicus*<br>*Rhinoceros unicornis*<br>*Elephas* sp.<br>*Stegodon orientalis*	
**Nam Lot**<br>**(86 – 72 ka)**	**Ruminant**	*Muntiacus muntjak*	*Rusa unicolor*<br>*Capricornis sumatraensis*	*Bos* sp.<br>*Bubalus bubalis*		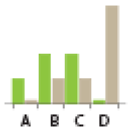
	**Non-ruminant**		*Sus scrofa*	*Tapirus* sp.	*Rhinoceros sondaicus*<br>*Rhinoceros unicornis*<br>*Elephas* sp.<br>*Stegodon* cf. *orientalis*	
**Duoi U'Oi**<br>**(70 – 60 ka)**<br>***Homo* sp.**	**Ruminant**	*Muntiacus muntjak*	*Rusa unicolor*<br>*Capricornis sumatraensis*	*Bubalus* cf. *bubalis*		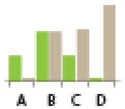
	**Non-ruminant**		*Sus scrofa*<br>*Sus barbatus*	*Tapirus indicus*<br>*Dicerorhinus sumatrensis*	*Rhinoceros sondaicus*<br>*Rhinoceros unicornis*<br>*Elephas* sp.	
**Tam Hay Marklot**<br>**(38.4 – 13.5 ka)**	**Ruminant**	*Muntiacus* sp.<br>*Axis* cf. *porcinus*<br>*Naemorhedus caudatus*	*Rusa unicolor*<br>*Rucervus eldii*<br>*Capricornis* cf. *sumatraensis*	*Bos* sp.<br>*Bubalus bubalis*		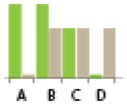
	**Non-ruminant**		*Sus* sp.<br>*Sus* cf. *barbatus*	*Tapirus* sp.<br>*Dicerorhinus sumatrensis*	*Rhinoceros sondaicus*<br>*Elephas* sp.	

Table S11: Lists and distributions of taxa by body size categories (A. 18-80 kg; B. 80-350 kg; C. 350-1000 kg; D. >1000 kg) (Faith et al. 2019), dietary strategy (ruminant (green) *versus* non-ruminant (brown)). No herbivore species smallest than ~20 kg is recorded in the southeast Asian fossil assemblages.

Tam Ngu Hao (Cobra)	MNI	%	Coc Muoi	MNI	%
large-sized cervid	3	20	*Rusa unicolor*	17	32,07
medium-sized cervid	2	13,33	medium-sized cervid	4	7,54
Muntiacus sp.	4	26,66	*Muntiacus* sp.	11	20,75
Bos sp.	5	33,33	*Bos* cf. *sauveli*	18	33,96
Naemorhedus sp.	1	6,66	*Capricornis* sp.	3	5,66
	**15**			**53**	
**Tam Hang South**	**MNI**	**%**	**Nam Lot**	**MNI**	**%**
Rusa unicolor	13	46,42	*Rusa unicolor*	4	16,66
medium-sized cervid	1	3,57	*Muntiacus* sp.	9	37,5
Muntiacus sp.	9	32,14	*Bos* sp.	2	8,33
Bos cf. *sauveli*	2	7,14	*Bubalus bubalis*	2	8,33
Bubalus bubalis	1	3,57	large-sized bovid	4	16,66
Capricornis sumatraensis	2	7,14	*Capricornis sumatraensis*	3	12,5
	**28**			**24**	
**Duoi U'Oi**	**MNI**	**%**	**Tam Hay Marklot**	**MNI**	**%**
Rusa unicolor	27	61,36	*Rusa unicolor*	29	38,66
Muntiacus sp.	14	31,81	*Rucervus eldii*	8	10,66
large-sized bovid	1	2,27	*Axis* cf. *porcinus*	5	6,66
Capricornis sp.	2	4,54	*Muntiacus* sp.	14	18,66
	**44**		*Bos* sp.	6	8
			large-sized bovid	2	2,66
			Capricornis sp.	7	9,33
			Naemorhedus cf. *caudatus*	4	5,33
				**75**	

Table S12. Minimum number of individuals (MNI) calculated by using the most frequent tooth type by taxon, and percentages of taxa belonging to Cervidae and Bovidae for all studied faunas. MNI data of Tam Hang South, Nam Lot and Duoi U'Oi (Bacon et al., 2015); MNI data of Coc Muoi (Bacon et al., 2018); MNI data of Tam Ngu Hao (Cobra) Cave and Tam Hay Marklot (the present paper).

References

Bacon A.-M. *et al.* The Late Pleistocene Duoi U'Oi cave in northern Vietnam: palaeontology, sedimentology, taphonomy, palaeoenvironments. *Quaternary Science Reviews* 27, 1627-1654 (2008).

Bacon A.-M. *et al.* Late Pleistocene mammalian assemblages of Southeast Asia: new dating, mortality profiles and evolution of the predator-prey relationships in an environmental context. *Palaeogeography, Palaeoclimatology, Palaeoecology* 422*,* 101-127 (2015).

Bacon A.-M. *et al.* A rhinocerotid-dominated megafauna at the MIS6-5 transition: The late Middle Pleistocene Coc Muoi assemblage, Lang Son province, Vietnam. *Quaternary Science Reviews* 186, 123-141 (2018).

Bacon A-M. *et al.* A multi-proxy approach to exploring *Homo sapiens'* arrival, environments and adaptations in Southeast Asia. *Scientific Reports* 11, 21080 (2021).

Bourgon N. *et al.* Zinc isotopes in Late Pleistocene fossil teeth from a Southeast Asian cave setting preserve paleodietary information. *Proceedings of National Academy of Sciences* 117*,* 4675-4681 (2020).

Bourgon N. *et al.* Trophic ecology of a Late Pleistocene early modern human from tropical Southeast Asia inferred from zinc isotopes. *Journal of Human Evolution* 161, 103075 (2021).

Bouteaux A., 2005. Paléontologie, paléoécologie et taphonomie des mammifères du Pléistocène moyen ancien du site à hominidés de Sangiran (Java central, Indonésie). Thèse du Muséum d'Histoire naturelle de Paris, 258 pages (2005).

Brain C. K. The hunters and the hunted? An introduction to African cave taphonomy. The University of Chicago press, Chicago and London, 1981.

Corbet G.B., Hill, J.E. The mammals of the Indomalayan region. Natural History Museum publications. Oxford University Press (1992).

Demeter F. *et al.* Anatomically modern human in Southeast Asia (Laos) by 46 ka. *Proceedings of the national academy of sciences* 109, 14375-14380 (2012).

Demeter F. *et al.* A Middle Pleistocene Denisovan from the Annamite Chain of northern Laos. *Nature communications*, 13: 2557 (2022).

Drucker D.G. The Isotopic Ecology of the Mammoth Steppe. *Annual Review of Earth and Planetary Sciences* 50, 395-418 (2022).

Duringer P. *et al.* Karst development, breccias history, and mammalian assemblages in Southeast Asia: A brief review. *Comptes Rendus Palevol* 11, 133-157 (2012).

Faith, J.T., Rowan, J., Du, A. Early hominins evolved within non-analog ecosystems. *Proceedings of the National Academy of Sciences* 116, 21478-21483 (2019).

Francis C.M. A field guide to the mammals of south-east Asia. New Holland Publishers (UK), 2008.

Jacobs Z. *et al.* Timing of archaic hominin occupation of Denisova Cave in southern Siberia. *Nature* 565, 594-599 (2019).

Janis C., 1984. Tapirs as living fossils. In Living fossils. Springer New York, pp. 80-86.

Janssen, R. *et al.* Tooth enamel stable isotopes of Holocene and Pleistocene fossil fauna reveal glacial and interglacial paleoenvironments of hominins in Indonesia. *Quat. Sci. Rev.* 144, 145–154 (2016).

Joordens, J.C.A. *et al.Homo erectus* at Trinil on Java used shells for tool production and engraving. *Nature* 518, 228–231 (2015).

Kaifu, Y. et al. Modern human teeth unearthed from below the ~128,000-year-old level at Punung, Java: A case highlighting the problem of recent intrusion in cave sediments. *Journal of Human Evolution* 163, 103122 (2022).

Kelly A. *et al.* Dietray paleoecology of bison and horses on the mammoth steppe of eastern Beringia based on dental microwear and mesowear analyses. *Palaeogeography, Palaeoclimatology, Palaeoecology* 572, 110394 (2021).

Kubat, J. et al. Dietary strategies of Pleistocene *Pongo* sp. and *Homo erectus* on Java (Indonesia). *Nature, Ecology* & *Evolution* 7, 279–289 (2023).

Louys, J. *et al.* Speleological and environmental history of Lida Ajer cave, western Sumatra. *Philosophical Transactions of the Royal Society B*. 377, 20200494 (2022).

Ma J. *et al.* The *Mammuthus-Coelodonta* Faunal Complex at its southeastern limit: A biogeochemical paleoecology investigation in Northeast Asia. *Quaternary International* 591, 93-106 (2021).

Matsu'ura et al., 2020, Age control of the first appearance datum for Javanese *Homo erectus* in the Sangiran area. Science 367, 210–2014 (2020).

Milano S. *et al.* Environmental conditions framing the first evidence of modern humans at Tam Pà Ling, Laos: A stable isotope record from terrestrial gastropod carbonates. *Palaeogeography, Palaeoclimatology, Palaeoecology* 511, 352-363 (2018).

Nowak R.M. Walker's Mammals of the World.Sixth Edition, Volume 1, The Johns Hopkins University Press (1999).

Puspaningrum, M.R. et al. Isotopic reconstruction of Proboscidean habitats and diets on Java since the Early Pleistocene: Implications for adaptations and extinction. *Quat. Sci. Rev.* 228, 106007 (2020).

Rizal, Y. et al. Last appearance of *Homo erectus* at Ngandong, Java, 117,000–108,000 years ago. *Nature* 577:381–385 (2020).

Rozzi, R. Space-time patterns of body size variation in island bovids: The key role of predatory release. *Journal of Biogeography* 45, 1196-1207 (2018).

Schepartz, L.A., Miller-Antonio, S. Large mamal exploitation in Late Middle Pleistocene China: A comparison of rhinoceros and stegodonts at Panxian Dadong. *Before farming* 4, 1-14 (2010).

Shackelford L. *et al.* Additional evidence for early modern human morphological diversity in Southeast Asia at Tam Pà Ling, Laos. *Quaternary International* 466, 93-106 (2018).

Smith A.T., Xie Y. A guide to the mammals of China. Princeton University Press, Princeton and Oxford, 2008.

Storm, P. et al. Late Pleistocene *Homo sapiens* in a tropical rainforest fauna in East Java. *Journal of Human Evolution* 49: 536–545 (2005).

Volmer R., Hertler, C., van der Geer, A. Niche overlap and competition potential among tigers (*Panthera tigris*), sabertoothed cats (*Homotherium ultimum*, *Hemimachairodus zwierzyckii*) and Merriam's Dog (*Megacyuon merriami*) in the Pleictocene of Java. *Palaeogreography, Palaeocimatology, Palaeoecology* 441, 901-911 (2016).

Westaway, K.E. et al. An early modern human presence in Sumatra 73,000–63,000 years ago. *Nature* 548, 322–325 (2017).

Westaway, K.E. et al. Age and biostratigraphic significance of the Punung Rainforest Fauna, East Java, Indonesia, and implications for *Pongo* and *Homo*. *Journal of Human Evolution* 53, 709–717 (2007).

Wood R. *et al.* The effect of grain size on carbonate contaminant removal from tooth enamel: Towards an improved pretreatment for radiocarbon dating. *Quaternary Geochronology* 36, 174-187 (2016).

Wood R. *et al*. Do weak or strong acids remove carbonate contamination from ancient tooth enamel more effectively? The effect of acid pretreatment on radiocarbon and 13C analyses. *Radiocarbon* 63, 935-952 (2021).

Zhang, D. *et al.* Denisovan DNA in Late Pleistocene sediments from Baishiya Karst Cave on the Tibetan Plateau. *Science* 370, 584–587 (2020).

Zavala E.I. *et al.* Pleistocene sediment DNA reveals hominin and faunal turnovers at Denisova Cave. *Nature* 595, 399-403 (2021).

Annex S1: Faunal list from the Tam Ngu Hao (Cobra) Cave with associated original data $\delta^{13}C_{apatite}$, $\delta^{13}C_{carbon\ source}$ and $\delta^{18}O_{apatite}$values (‰ VPDB), as well as body mass and $\delta^{13}C$ (‰ VPDB) Enrichment Factor used to obtain $\delta^{13}C_{carbon\ source}$.

Number	Country	Site	Taxon	Body Mass (kg)	$\delta^{13}C$ (‰) Enrichment Factor	$\delta^{13}C_{carbon\ source}$ (‰ VPDB)	$\delta^{13}C_{apatite}$ (‰ VPDB)	$\delta^{18}O_{apatite}$ (‰ VPDB)
TNH154	Laos	Cobra Cave	*Ailuropoda melanoleuca*	92	9.7	-26.2	-16.54	-6.02
TNH152	Laos	Cobra Cave	*Ailuropoda melanoleuca*	92	9.7	-23.8	-14.1	-6.43
TNH69	Laos	Cobra Cave	*Bos* sp.	800	14.5	-30.4	-15.9	-3.44
TNH65	Laos	Cobra Cave	*Bos* sp.	800	14.5	-27.5	-12.99	-4.81
TNH62	Laos	Cobra Cave	*Bos* sp.	800	14.5	-25.9	-11.44	-9.19
TNH80	Laos	Cobra Cave	*Bos* sp.	800	14.5	-23.3	-8.83	-5.94
TNH68	Laos	Cobra Cave	*Bos* sp.	800	14.5	-18.6	-4.05	-5.52
TNH86	Laos	Cobra Cave	*Bos* sp.	800	14.5	-14.3	+0.18	-4.81
TNH66	Laos	Cobra Cave	*Bos* sp.	800	14.5	-14.0	+0.53	-8.54
TNH87	Laos	Cobra Cave	Cervidae	220	13.59	-26.5	-12.95	-8.85
TNH119	Laos	Cobra Cave	*Hystrix* sp.	12	12.18	-26.5	-14.33	-9.48
TNH144	Laos	Cobra Cave	*Hystrix* sp.	12	12.18	-25.3	-13.13	-10.49
TNH149	Laos	Cobra Cave	*Hystrix* sp.	12	12.18	-25.0	-12.8	-9.86
TNH151	Laos	Cobra Cave	*Hystrix* sp.	12	12.18	-24.8	-12.62	-9.82
TNH137	Laos	Cobra Cave	*Hystrix* sp.	12	12.18	-23.9	-11.74	-5.925
TNH20	Laos	Cobra Cave	Large-sized Bovidae	800	14.5	-29.2	-14.68	-7.26
TNH41	Laos	Cobra Cave	Large-sized Cervidae	220	13.59	-30.2	-16.57	-8.17
TNH83	Laos	Cobra Cave	Large-sized Cervidae	220	13.59	-29.6	-15.97	-6.4
TNH40	Laos	Cobra Cave	Large-sized Cervidae	220	13.59	-27.9	-14.35	-8.74
TNH39	Laos	Cobra Cave	Large-sized Cervidae	220	13.59	-26.4	-12.8	-6.78
TNH45	Laos	Cobra Cave	Large-sized Cervidae	220	13.59	-25.4	-11.83	-3.11
TNH44	Laos	Cobra Cave	Large-sized Cervidae	220	13.59	-15.3	-1.7	-5.11
TNH6	Laos	Cobra Cave	*Macaca* sp.	6	11.91	-27.6	-15.64	-6.53
TNH5	Laos	Cobra Cave	*Macaca* sp.	6	11.91	-27.5	-15.55	-6.25
TNH160	Laos	Cobra Cave	*Macaca* sp.	6	11.91	-26.4	-14.53	-4.8

TNH161	Laos	Cobra Cave	*Macaca* sp.	6	11.91	-26.0	-14.12	-3.58
TNH33	Laos	Cobra Cave	Medium-sized Cervidae	83	12.9	-27.4	-14.42	-9.56
TNH29	Laos	Cobra Cave	Medium-sized Cervidae	83	12.9	-17.3	-4.34	-5.06
TNH14	Laos	Cobra Cave	*Muntiacus* sp.	220	13.59	-28.5	-14.92	-7.75
TNH57	Laos	Cobra Cave	*Muntiacus* sp.	24	12.17	-25.1	-12.96	-9.02
TNH55	Laos	Cobra Cave	*Muntiacus* sp.	24	12.17	-25.0	-12.78	-6.04
TNH181	Laos	Cobra Cave	*Pongo* sp.	55	12.78	-26.7	-13.96	-4.28
TNH179	Laos	Cobra Cave	*Rhinoceros* sp.	1650	14.25	-28.1	-13.8	-8.53
TNH172	Laos	Cobra Cave	Rhinocerotidae indet.	1650	14.25	-31.3	-17.09	-7.11
TNH171	Laos	Cobra Cave	Rhinocerotidae indet.	1650	14.25	-30.4	-16.1	-6.05
TNH180	Laos	Cobra Cave	Rhinocerotidae indet.	1650	14.25	-29.6	-15.33	-7.29
TNH76	Laos	Cobra Cave	Small-sized Caprinae	27	12.24	-28.4	-16.13	-6.21
TNH77	Laos	Cobra Cave	Small-sized Caprinae	27	12.24	-27.5	-15.24	-2.9
TNH73	Laos	Cobra Cave	Small-sized Caprinae	27	12.24	-14.8	-2.55	-2.55
TNH75	Laos	Cobra Cave	Small-sized Caprinae	27	12.24	-11.9	+0.37	-8.02
TNH22	Laos	Cobra Cave	Small-sized carnivora	17	13.44	-27.6	-14.14	-3.92
TNH9	Laos	Cobra Cave	Small-sized carnivora	17	13.44	-26.6	-13.16	-10.39
TNH166	Laos	Cobra Cave	Small-sized Felidae	18	13.46	-21.8	-8.38	-3.92
TNH8	Laos	Cobra Cave	*Stegodon* sp.	4000	14.66	-28.6	-13.95	-7.66
TNH130	Laos	Cobra Cave	*Sus* sp.	137	13.19	-27.3	-14.09	-7.3
TNH125	Laos	Cobra Cave	*Sus* sp.	137	13.19	-26.5	-13.34	-7.25
TNH129	Laos	Cobra Cave	*Sus* sp.	137	13.19	-26.5	-13.35	-5.95
TNH127	Laos	Cobra Cave	*Sus* sp.	137	13.19	-26.4	-13.23	-8.57
TNH119	Laos	Cobra Cave	*Sus* sp.	137	13.19	-26.2	-13.0	-6.91
TNH120	Laos	Cobra Cave	*Sus* sp.	137	13.19	-25.9	-12.7	-8.32
TNH89	Laos	Cobra Cave	*Sus* sp.	137	13.19	-25.7	-12.49	-7.66
TNH111	Laos	Cobra Cave	*Sus* sp.	137	13.19	-25.1	-11.94	-7.4
TNH178	Laos	Cobra Cave	*Tapirus indicus*	300	13.5	-30.0	-16.46	-5.54
TNH2-1	Laos	Cobra Cave	TNH2-1	62	12.7	-16.28	-3.58	-7.03

Annex S2. Ungulate specimens from already-published Tam Pà Ling site (Bourgon et al., 2021) with associated $\delta^{13}C_{apatite}$, $\delta^{13}C_{carbon\ source}$ and $\delta^{18}O_{apatite}$values, as well as body mass and $\delta^{13}C$ (‰), and enrichment factor used to obtain $\delta^{13}C_{carbon\ source}$. S-EVA, Stable Isotope-Evolutionary Anthropology (Max Planck Institute for Evolutionary Anthropology). The age range of teeth are from Shackelford et al., 2018.

S-EVA	Country	Site	Depth (metre)	Optical ages (OSL, pIR-IRSL)	Taxon	Body mass (kg)	$\delta^{13}C_{apatite}$ (‰)	$\delta^{18}O_{apatite}$ (‰)	$\delta^{13}C$ (‰) Enrichment factor	$\delta^{13}C_{carbon\ source}$ (‰)
35419	Laos	Tam Pà Ling	2.05	33 ± 3 ka	*Naemorhedus* sp.	27	–16.3	–2.1	12.2	–28.5
35417	Laos	Tam Pà Ling	2.22	43–33 ka	*Capricornis* sp.	112	–13.6	–6.2	13.1	–26.7
35946	Laos	Tam Pà Ling	2.32	43–33 ka	*Capricornis* sp.	112	–14.0	–6.5	13.1	–27.1
35492	Laos	Tam Pà Ling	2.35	46–43 ka	*Homo sapiens*	62	–13.7	–6.4	12.7	–26.4
35427	Laos	Tam Pà Ling	2.44	46–43 ka	*Rhinoceros* cf. *unicornis*	2250	–15.8	–9.1	14.4	–30.2
35416	Laos	Tam Pà Ling	2.50	46–43 ka	*Capricornis* sp.	112	–12.9	–5.8	13.1	–26.0
35428	Laos	Tam Pà Ling	2.51	46–43 ka	*Dicerorhinus sumatrensis*	950	–15.8	–8.9	14.0	–29.8
65952	Laos	Tam Pà Ling	2.59	46 ± 4 ka	Caprinae	70	–13.9	–4.2	12.8	–26.7
35947	Laos	Tam Pà Ling	2.78	46 ± 4 ka	Caprinae	70	–14.7	–6.3	12.8	–27.5
35418	Laos	Tam Pà Ling	3.00	46 ± 4 ka	*Naemorhedus* sp.	27	–5.0	–3.1	12.2	–17.2
35423	Laos	Tam Pà Ling	3.29	56–46 ka	Caprinae	70	–5.5	–0.6	12.8	–18.3
35426	Laos	Tam Pà Ling	3.50	56–46 ka	*Naemorhedus* sp.	27	–14.6	–3.3	12.2	–26.8
35422	Laos	Tam Pà Ling	3.80	56–46 ka	Bovidae indet.	875	–15.0	–2.7	14.6	–29.6
35429	Laos	Tam Pà Ling	4.20	56 ± 6 ka	Caprinae	70	–14.0	–8.0	12.8	–26.9
35958	Laos	Tam Pà Ling	6.60	>70 ± 8 ka	*Rhinoceros sondaicus*	1750	–15.7	–3.6	14.3	–30.0

Annex S3. Faunal lists from other sites (Duoi U'Oi, Coc Muoi, Tam Hang South) with already-published data (Bacon et al., 2021), with associated $\delta^{13}C_{apatite}$, $\delta^{13}C_{carbon\ source}$ and $\delta^{18}O_{apatite}$values (‰ VPDB), as well as body mass and $\delta^{13}C$ (‰ VPDB) Enrichment Factor used to obtain $\delta^{13}C_{carbon\ source}$.

Number	Country	Site	Taxon	Body Mass (kg)	$\delta^{13}C_{apatite}$ (‰ VPDB)	$\delta^{13}C$ (‰) Enrichment Factor	$\delta^{13}C_{carbon\ source}$ (‰ VPDB)	$\delta^{18}O_{apatite}$ (‰ VPDB)
DU876	Vietnam	Duoi U'Oi	*Sus scrofa*	137	-14.2	13.19	-27.4	-5.1
DU890	Vietnam	Duoi U'Oi	*Sus scrofa*	137	-12.8	13.19	-26.0	-8.3
DU905	Vietnam	Duoi U'Oi	*Sus scrofa*	137	-13.7	13.19	-26.9	-7.1
DU906	Vietnam	Duoi U'Oi	*Sus scrofa*	137	-15.0	13.19	-28.2	-6.0
DU913	Vietnam	Duoi U'Oi	*Sus scrofa*	137	-14.7	13.19	-27.9	-6.2
DU546	Vietnam	Duoi U'Oi	*Rusa unicolor*	220	-16.6	13.59	-30.2	-7.1
DU557	Vietnam	Duoi U'Oi	*Rusa unicolor*	220	-19.6	13.59	-33.2	-6.1
DU567	Vietnam	Duoi U'Oi	*Rusa unicolor*	220	-18.1	13.59	-31.7	-6.1
DU608	Vietnam	Duoi U'Oi	*Rusa unicolor*	220	-18.1	13.59	-31.7	-7.0
DU990	Vietnam	Duoi U'Oi	*Rusa unicolor*	220	-18.3	13.59	-31.9	-5.7
DU1087	Vietnam	Duoi U'Oi	*Rusa unicolor*	220	-17.7	13.59	-31.3	-8.5
DU437	Vietnam	Duoi U'Oi	*Muntiacus muntjak*	24	-13.2	12.17	-25.4	-9.3
DU461	Vietnam	Duoi U'Oi	*Muntiacus muntjak*	24	-14.8	12.17	-27.0	-7.7
DU511	Vietnam	Duoi U'Oi	*Muntiacus muntjak*	24	-14.6	12.17	-26.8	-5.1
DU433	Vietnam	Duoi U'Oi	*Muntiacus muntjak*	24	-13.8	12.17	-26.0	-7.8
DU392	Vietnam	Duoi U'Oi	*Muntiacus muntjak*	24	-13.0	12.17	-25.2	-6.5
DU574	Vietnam	Duoi U'Oi	*Capricornis sumatraensis*	112	-15.8	13.14	-28.9	-7.1
DU613	Vietnam	Duoi U'Oi	*Capricornis sumatraensis*	112	-17.4	13.14	-30.5	-7.5
DU538	Vietnam	Duoi U'Oi	*Capricornis sumatraensis*	112	-14.5	13.14	-27.6	-6.1
DU992	Vietnam	Duoi U'Oi	Bovidae indet.	875	-19.8	14.57	-34.4	-7.1
DU983	Vietnam	Duoi U'Oi	Bovidae indet.	875	-12.1	14.57	-26.7	-5.2
DU984	Vietnam	Duoi U'Oi	Bovidae indet.	875	-0.6	14.57	-15.2	-4.7
DU560	Vietnam	Duoi U'Oi	Bovidae indet.	875	-19.3	14.57	-33.9	-2.5
	Vietnam	Duoi U'Oi	*Hystrix brachyura*	12	-16.7	12.18	-28.9	-8.0
	Vietnam	Duoi U'Oi	*Hystrix brachyura*	12	-12.6	12.18	-24.8	-6.7
	Vietnam	Duoi U'Oi	*Hystrix brachyura*	12	-16.26	12.18	-28.4	-9.71
	Vietnam	Duoi U'Oi	*Hystrix brachyura*	12	-12.8	12.18	-25.0	-6.39
	Vietnam	Duoi U'Oi	*Hystrix brachyura*	12	-17.08	12.18	-29.3	-8.68
	Vietnam	Duoi U'Oi	*Hystrix brachyura*	12	-13.7	12.18	-25.9	-6.6

	Vietnam	Duoi U'Oi	*Hystrix brachyura*	12	-17.3	12.18	-29.5	-5.1
DU7	Vietnam	Duoi U'Oi	*Hystrix brachyura*	12	-14.7	12.18	-26.9	-8.9
DU728	Vietnam	Duoi U'Oi	*Ursus* sp.	100	-15.0	13.3	-28.3	-6.5
DU729	Vietnam	Duoi U'Oi	*Ursus* sp.	100	-14.9	13.3	-28.2	-9.3
DU1152	Vietnam	Duoi U'Oi	*Cuon alpinus*	15	-14.2	13.39	-27.6	-7.3
DU1153	Vietnam	Duoi U'Oi	*Cuon alpinus*	15	-14.9	13.39	-28.3	-6.7
DU77	Vietnam	Duoi U'Oi	*Cuon alpinus*	15	-14.5	13.39	-27.9	-7.4
DU68	Vietnam	Duoi U'Oi	*Panthera pardus*	41	-14.2	13.81	-28.0	-5.2
DU86	Vietnam	Duoi U'Oi	*Panthera pardus*	41	-13.9	13.81	-27.7	-5.2
DU707	Vietnam	Duoi U'Oi	*Panthera tigris*	212	-15.5	14.53	-30.0	-5.4
DU326	Vietnam	Duoi U'Oi	*Macaca* sp.	6	-11.9	11.91	-23.8	-6.2
DU331	Vietnam	Duoi U'Oi	*Macaca* sp.	6	-15.6	11.91	-27.5	-5.1
DU339	Vietnam	Duoi U'Oi	*Macaca* sp.	6	-14.4	11.91	-26.3	-4.4
DU343	Vietnam	Duoi U'Oi	*Macaca* sp.	6	-15.4	11.91	-27.3	-5.0
DU322	Vietnam	Duoi U'Oi	*Macaca* sp.	6	-16.0	11.91	-27.9	-6.3
DU32	Vietnam	Duoi U'Oi	*Rhinoceros unicornis*	2250	-17.1	14.4	-31.5	-7.2
DU26	Vietnam	Duoi U'Oi	*Rhinoceros unicornis*	2250	-16.1	14.4	-30.5	-6.4
DU28	Vietnam	Duoi U'Oi	*Rhinoceros unicornis*	2250	-16.2	14.4	-30.6	-6.6
DU30	Vietnam	Duoi U'Oi	*Rhinoceros sondaicus*	2250	-15.7	14.4	-30.1	-6.4
DU31	Vietnam	Duoi U'Oi	*Rhinoceros sondaicus*	2250	-15.3	14.4	-29.7	-4.9
DU38	Vietnam	Duoi U'Oi	*Rhinoceros sondaicus*	2250	-16.6	14.4	-31.0	-7.0
DU27	Vietnam	Duoi U'Oi	*Dicerorhinus sumatrensis*	950	-17.5	14	-31.5	-7.9
DU24	Vietnam	Duoi U'Oi	*Dicerorhinus sumatrensis*	950	-16.4	14	-30.4	-6.6
DU47	Vietnam	Duoi U'Oi	*Tapirus indicus*	300	-17.3	13.5	-30.8	-7.9
DU43	Vietnam	Duoi U'Oi	*Tapirus indicus*	300	-20.1	13.5	-33.6	-6.9
DU53	Vietnam	Duoi U'Oi	*Tapirus indicus*	300	-19.1	13.5	-32.6	-8.3
DU1021	Vietnam	Duoi U'Oi	*Pongo pygmaeus*	55	-14.6	12.78	-27.4	-6.0
DU1022	Vietnam	Duoi U'Oi	*Pongo pygmaeus*	55	-14.5	12.78	-27.3	-5.1
DU1023	Vietnam	Duoi U'Oi	*Pongo pygmaeus*	55	-14.3	12.17	-26.5	-4.9
DU634	Vietnam	Duoi U'Oi	*Elephas* sp.	4250	-16.9	14.69	-31.6	-7.0
DU-	Vietnam	Duoi U'Oi	*Elephas* sp.	4250	-15.9	14.69	-30.6	-6.8
CM169	Vietnam	Coc Muoi	*Muntiacus* sp.	24	-14.2	12.17	-26.4	-7.1
CM307	Vietnam	Coc Muoi	*Muntiacus* sp.	24	-14.5	12.17	-26.7	-6.5
CM357	Vietnam	Coc Muoi	*Muntiacus* sp.	24	-13.5	12.17	-25.7	-5.7
Cm313	Vietnam	Coc Muoi	*Muntiacus* sp.	24	-14.0	12.17	-26.2	-5.5

CM175	Vietnam	Coc Muoi	*Muntiacus* sp.	24	-13.9	12.17	-26.1	-6.9
CM176	Vietnam	Coc Muoi	*Muntiacus* sp.	24	-13.2	12.17	-25.4	-4.4
CM324	Vietnam	Coc Muoi	*Muntiacus* sp.	24	-16.5	12.17	-28.7	-6.2
CM450	Vietnam	Coc Muoi	*Sus scrofa*	137	-14.5	13.19	-27.7	-6.2
CM676	Vietnam	Coc Muoi	*Sus scrofa*	137	-13.9	13.19	-27.1	-6.9
CM677	Vietnam	Coc Muoi	*Sus scrofa*	137	-13.8	13.19	-27.0	-6.5
CM678	Vietnam	Coc Muoi	*Sus scrofa*	137	-13.1	13.19	-26.3	-6.4
CM748	Vietnam	Coc Muoi	*Sus scrofa*	137	-13.7	13.19	-26.9	-7.2
CM746	Vietnam	Coc Muoi	*Sus scrofa*	137	-13.9	13.19	-27.1	-6.2
CM543	Vietnam	Coc Muoi	*Ailuropoda melanoleuca*	92	-17.4	10.51	-27.9	-4.2
CM544	Vietnam	Coc Muoi	*Ailuropoda melanoleuca*	92	-17.7	10.51	-28.2	-5.6
CM564	Vietnam	Coc Muoi	*Ailuropoda melanoleuca*	92	-18.5	10.51	-29.0	-6.7
CM415	Vietnam	Coc Muoi	*Ursus* sp. (? *thibetanus*)	100	-14.5	13.3	-27.8	-5.8
CM518	Vietnam	Coc Muoi	*Ursus thibetanus*	100	-17.0	13.3	-30.3	-6.9
CM519	Vietnam	Coc Muoi	*Ursus thibetanus*	100	-13.4	13.3	-26.7	-6.4
CM521	Vietnam	Coc Muoi	*Ursus* sp. (? *thibetanus*)	100	-14.4	13.3	-27.7	-8.5
CM525	Vietnam	Coc Muoi	*Ursus thibetanus*	100	-14.1	13.3	-27.4	-6.8
CM508	Vietnam	Coc Muoi	*Pongo devosi*	55	-15.3	12.78	-28.1	-4.0
CM579	Vietnam	Coc Muoi	*Pongo devosi*	55	-15.0	12.78	-27.8	-4.7
CM551	Vietnam	Coc Muoi	*Pongo devosi*	55	-14.8	12.78	-27.6	-4.8
CM553	Vietnam	Coc Muoi	*Pongo devosi*	55	-15.0	12.78	-27.8	-5.0
CM509	Vietnam	Coc Muoi	*Macaca* sp.	6	-14.0	11.91	-25.9	-4.7
CM577	Vietnam	Coc Muoi	*Macaca* sp.	6	-13.8	11.91	-25.7	-7.4
CM614	Vietnam	Coc Muoi	*Macaca* sp.	6	-14.2	11.91	-26.1	-5.9
CM618	Vietnam	Coc Muoi	*Macaca* sp.	6	-15.1	11.91	-27.0	-5.2
CM555	Vietnam	Coc Muoi	*Macaca* sp.	6	-15.4	11.91	-27.3	-4.7
CM534	Vietnam	Coc Muoi	*Panthera tigris*	212	-16.2	14.53	-30.7	-7.5
CM566	Vietnam	Coc Muoi	*Panthera tigris*	212	-14.6	14.53	-29.1	-5.4
CM632	Vietnam	Coc Muoi	*Panthera tigris*	212	-16.0	14.53	-30.5	-8.6
CM571	Vietnam	Coc Muoi	*Cuon alpinus*	15	-14.3	13.39	-27.7	-6.7
CM535	Vietnam	Coc Muoi	Small-sized Felidae	18	-14.5	13.46	-28.0	-8.6
CM536	Vietnam	Coc Muoi	Small-sized Felidae	18	-13.2	13.46	-26.7	-6.2
CM421	Vietnam	Coc Muoi	*Hystrix* sp.	12	-14.8	12.18	-27.0	-8.1
CM472	Vietnam	Coc Muoi	*Hystrix* sp.	12	-15.8	12.18	-28.0	-3.9
CM902	Vietnam	Coc Muoi	*Hystrix* sp.	12	-16.0	12.18	-28.2	-7.2

CM903	Vietnam	Coc Muoi	*Hystrix* sp.	12	-14.5	12.18	-26.7	-7.5
CM904	Vietnam	Coc Muoi	*Hystrix* sp.	12	-16.0	12.18	-28.2	-4.1
CM910	Vietnam	Coc Muoi	*Hystrix* sp.	12	-13.6	12.18	-25.8	-8.6
CM911	Vietnam	Coc Muoi	*Hystrix* sp.	12	-13.7	12.18	-25.9	-4.6
CM954	Vietnam	Coc Muoi	*Hystrix* sp.	12	-14.7	12.18	-26.9	-4.9
CM983	Vietnam	Coc Muoi	*Atherurus* sp.	12	-14.6	12.18	-26.8	-5.8
CM984	Vietnam	Coc Muoi	*Atherurus* sp.	12	-14.1	12.18	-26.3	-4.6
CM75	Vietnam	Coc Muoi	*Rusa unicolor*	220	-17.5	13.59	-31.1	-6.8
CM244	Vietnam	Coc Muoi	*Rusa unicolor*	220	-13.4	13.59	-27.0	-6.9
CM245	Vietnam	Coc Muoi	*Rusa unicolor*	220	-15.2	13.59	-28.8	-6.5
CM246	Vietnam	Coc Muoi	*Rusa unicolor*	220	-14.0	13.59	-27.6	-5.8
CM247	Vietnam	Coc Muoi	*Rusa unicolor*	220	-17.0	13.59	-30.6	-7.8
CM249	Vietnam	Coc Muoi	*Rusa unicolor*	220	-16.3	13.59	-29.9	-7.3
CM287	Vietnam	Coc Muoi	*Capricornis* sp.	112	-15.0	13.14	-28.1	-5.9
CM173	Vietnam	Coc Muoi	*Capricornis* sp.	112	-17.7	13.14	-30.8	-5.1
CM286	Vietnam	Coc Muoi	*Capricornis* sp.	112	-17.9	13.14	-31.0	-7.6
CM147	Vietnam	Coc Muoi	*Capricornis* sp.	112	-14.5	13.14	-27.6	-4.9
CM149	Vietnam	Coc Muoi	*Capricornis* sp.	112	-17.1	13.14	-30.2	-5.5
CM370	Vietnam	Coc Muoi	*Bos* cf. *sauveli*	800	-14.8	14.5	-29.3	-5.8
CM225	Vietnam	Coc Muoi	*Bos* cf. *sauveli*	800	-8.9	14.5	-23.4	-6.6
CM226	Vietnam	Coc Muoi	*Bos* cf. *sauveli*	800	-11.1	14.5	-25.6	-5.9
CM227	Vietnam	Coc Muoi	*Bos* cf. *sauveli*	800	-11.3	14.5	-25.8	-6.9
CM46	Vietnam	Coc Muoi	*Bos* cf. *sauveli*	800	-3.6	14.5	-18.1	-4.1
CM152a	Vietnam	Coc Muoi	Bovidae (?*Bos sauveli*)	875	-13.4	14.57	-28.0	-5.9
CM1067	Vietnam	Coc Muoi	*Rhinoceros sondaicus*	1750	-17.0	14.28	-31.3	-5.4
CM1122	Vietnam	Coc Muoi	*Rhinoceros sondaicus*	1750	-16.6	14.28	-30.9	-4.9
CM1120	Vietnam	Coc Muoi	*Rhinoceros sondaicus*	1750	-15.6	14.28	-29.9	-5.8
CM1068	Vietnam	Coc Muoi	*Rhinoceros sondaicus*	1750	-15.5	14.28	-29.8	-5.8
CM1229	Vietnam	Coc Muoi	*Rhinoceros sondaicus*	1750	-16.3	14.28	-30.6	-4.7
CM1137	Vietnam	Coc Muoi	*Rhinoceros sondaicus*	1750	-17.0	14.28	-31.3	-7.8
CM1339	Vietnam	Coc Muoi	*Rhinoceros sondaicus*	1750	-16.5	14.28	-30.8	-5.4
CM998	Vietnam	Coc Muoi	*Rhinoceros sondaicus*	1750	-16.4	14.28	-30.7	-7.1
CM1151	Vietnam	Coc Muoi	*Rhinoceros unicornis*	2250	-16.3	14.4	-30.7	-5.8
CM1278	Vietnam	Coc Muoi	*Rhinoceros unicornis*	2250	-16.8	14.4	-31.2	-6.2
CM1035	Vietnam	Coc Muoi	*Dicerorhinus sumatrensis*	950	-15.8	14	-29.8	-6.4

CM1096	Vietnam	Coc Muoi	*Dicerorhinus sumatrensis*	950	-15.6	14	-29.6	-5.0
CM1349	Vietnam	Coc Muoi	*Megatapirus augustus*	500	-20.1	13.72	-33.8	-5.9
CM514	Vietnam	Coc Muoi	*Megatapirus augustus*	500	-18.7	13.72	-32.4	-5.5
CM515	Vietnam	Coc Muoi	*Tapirus indicus*	300	-17.8	13.5	-31.3	-6.2
CM516	Vietnam	Coc Muoi	*Tapirus indicus*	300	-18.3	13.5	-31.8	-6.8
CM1351	Vietnam	Coc Muoi	*Tapirus indicus*	300	-16.4	13.5	-29.9	-6.6
CM726	Vietnam	Coc Muoi	*Stegodon* sp.	4000	-14.2	14.66	-28.9	-7.9
CM637	Vietnam	Coc Muoi	*Stegodon* sp.	4000	-14.7	14.66	-29.4	-9.8
CM728	Vietnam	Coc Muoi	*Elephas maximus*	4250	-18.3	14.69	-33.0	-5.8
420	Vietnam	Coc Muoi	*Elephas maximus*	4250	-15.8	14.69	-30.5	-6.0
TH-860	Laos	Tam Hang South	*Cuon alpinus* cf. *antiquus*	15	-12.9	13.39	-26.3	-6.8
TH-861	Laos	Tam Hang South	*Cuon alpinus* cf. *antiquus*	15	-14.4	13.39	-27.8	-2.9
TH-870	Laos	Tam Hang South	*Cuon alpinus* cf. *antiquus*	15	-13.0	13.39	-26.4	-6.3
TH-539	Laos	Tam Hang South	*Capricornis sumatraensis*	112	-6.8	13.14	-19.9	-4.3
TH-767	Laos	Tam Hang South	*Capricornis sumatraensis*	112	-4.9	13.14	-18.0	-2.8
TH-768	Laos	Tam Hang South	*Capricornis sumatraensis*	112	-13.7	13.14	-26.8	-5.7
TH-873	Laos	Tam Hang South	*Helarctos malayanus*	50	-12.6	13.3	-25.9	-5.9
TH-877	Laos	Tam Hang South	*Helarctos malayanus*	50	-12.5	13.3	-25.8	-7.2
TH-H1	Laos	Tam Hang South	*Hystrix brachyura*	12	-13.7	12.18	-25.9	-5.3
TH-H2	Laos	Tam Hang South	*Hystrix brachyura*	12	-9.9	12.18	-22.1	-9.4
TH-H3	Laos	Tam Hang South	*Hystrix brachyura*	12	-10.2	12.18	-22.4	-5.4
TH-H4	Laos	Tam Hang South	*Hystrix brachyura*	12	-8.9	12.18	-21.1	-8.9
TH-H5	Laos	Tam Hang South	*Hystrix brachyura*	12	-14.7	12.18	-26.9	-6.7
TH-H6	Laos	Tam Hang South	*Hystrix brachyura*	12	-14	12.18	-26.2	-6.6
TH-789	Laos	Tam Hang South	*Bubalus bubalis*	1000	-2.9	14.66	-17.6	-6.7
TH-791	Laos	Tam Hang South	*Bubalus bubalis*	1000	-5.0	14.66	-19.7	-6.8
TH-455	Laos	Tam Hang South	*Bos* cf. *sauveli*	800	+1.8	14.5	-12.7	-5.7
TH-458	Laos	Tam Hang South	*Bos* cf. *sauveli*	800	-10.9	14.5	-25.4	-8.2
TH-459	Laos	Tam Hang South	*Bos* cf. *sauveli*	800	-11.8	14.5	-26.3	-7.3
TH-546	Laos	Tam Hang South	*Bos* cf. *sauveli*	800	-14.9	14.5	-29.4	-6.4
TH-573	Laos	Tam Hang South	*Bos* cf. *sauveli*	800	-9.9	14.5	-24.4	-7.1
TH-790	Laos	Tam Hang South	*Bos* cf. *sauveli*	800	-12.9	14.5	-27.4	-8.2
TH-799	Laos	Tam Hang South	*Bos* cf. *sauveli*	800	-2.8	14.5	-17.3	-6.3
TH-72	Laos	Tam Hang South	*Macaca* sp.	6	-14.0	11.91	-25.9	-5.7
TH-73	Laos	Tam Hang South	*Macaca* sp.	6	-13.4	11.91	-25.3	-5.0

TH-74	Laos	Tam Hang South	*Macaca* sp.	6	-14.1	11.91	-26.0	-5.0
TH-75	Laos	Tam Hang South	*Macaca* sp.	6	-14.0	11.91	-25.9	-5.3
TH-76	Laos	Tam Hang South	*Macaca* sp.	6	-13.5	11.91	-25.4	-5.1
TH-79	Laos	Tam Hang South	*Macaca* sp.	6	-14.2	11.91	-26.1	-5.3
TH-593	Laos	Tam Hang South	*Megatapirus augustus*	500	-15.8	13.72	-29.5	-7.5
TH-213	Laos	Tam Hang South	*Muntiacus muntjak*	24	-13.2	12.17	-25.4	-6.8
TH-215	Laos	Tam Hang South	*Muntiacus muntjak*	24	-12.6	12.17	-24.8	-7.5
TH-216	Laos	Tam Hang South	*Muntiacus muntjak*	24	-12.8	12.17	-25.0	-7.7
TH-219	Laos	Tam Hang South	*Muntiacus muntjak*	24	-13.3	12.17	-25.5	-8.1
TH-130	Laos	Tam Hang South	*Panthera tigris*	212	-14.1	14.53	-28.6	-7.8
TH-132	Laos	Tam Hang South	*Panthera tigris*	212	-14.0	14.53	-28.5	-7.4
TH-376	Laos	Tam Hang South	*Rhinoceros* spp.	2000	-13.5	14.34	-27.8	-6.8
TH-379	Laos	Tam Hang South	*Rhinoceros* spp.	2000	-13.3	14.34	-27.6	-7.0
TH-576	Laos	Tam Hang South	*Rhinoceros* spp.	2000	-14.4	14.34	-28.7	-7.7
TH-445	Laos	Tam Hang South	*Rusa unicolor*	220	-14.5	13.59	-28.1	-5.0
TH-469	Laos	Tam Hang South	*Rusa unicolor*	220	-12.7	13.59	-26.3	-6.8
TH-499	Laos	Tam Hang South	*Rusa unicolor*	220	-4.8	13.59	-18.4	-8.2
TH-549	Laos	Tam Hang South	*Rusa unicolor*	220	-15.8	13.59	-29.4	-6.1
TH-555	Laos	Tam Hang South	*Rusa unicolor*	220	-16.2	13.59	-29.8	-6.7
TH-717	Laos	Tam Hang South	*Rusa unicolor*	220	-15.0	13.59	-28.6	-8.8
TH-719	Laos	Tam Hang South	*Rusa unicolor*	220	-13.5	13.59	-27.1	-6.5
TH-748	Laos	Tam Hang South	*Rusa unicolor*	220	-13.0	13.59	-26.6	-8.6
TH-390-2	Laos	Tam Hang South	*Sus scrofa*	137	-12.7	13.19	-25.9	-8.6
TH-410	Laos	Tam Hang South	*Sus scrofa*	137	-12.6	13.19	-25.8	-8.2
TH-420	Laos	Tam Hang South	*Sus scrofa*	137	-1.4	13.19	-14.6	-6.3
TH-427	Laos	Tam Hang South	*Sus scrofa*	137	-12.0	13.19	-25.2	-6.2
TH-430	Laos	Tam Hang South	*Sus scrofa*	137	-11.8	13.19	-25.0	-8.5
TH-431	Laos	Tam Hang South	*Sus scrofa*	137	-12.3	13.19	-25.5	-6.7
TH-433	Laos	Tam Hang South	*Sus scrofa*	137	-12.7	13.19	-25.9	-8.9
TH-637	Laos	Tam Hang South	*Sus scrofa*	137	-13.2	13.19	-26.4	-7.8
TH-642	Laos	Tam Hang South	*Sus scrofa*	137	-11.2	13.19	-24.4	-5.3
TH-645-2	Laos	Tam Hang South	*Sus scrofa*	137	-12.3	13.19	-25.5	-8.1
TH-646	Laos	Tam Hang South	*Sus scrofa*	137	-12.7	13.19	-25.9	-8.5
TH-371	Laos	Tam Hang South	*Tapirus indicus* cf. *intermedius*	300	-16.5	13.5	-30.0	-6.5
TH-378	Laos	Tam Hang South	*Tapirus indicus* cf. *intermedius*	300	-16.3	13.5	-29.8	-5.6

TH-129	Laos	Tam Hang South	*Ursus thibetanus* cf. *kokeni*	100	-14.4	13.3	-27.7	-8.5
TH-139	Laos	Tam Hang South	*Ursus thibetanus* cf. *kokeni*	100	-14.4	13.3	-27.7	-6.1

Annex S4. Faunal lists from other sites (Nam Lot I, Tam Hay Marklot) with already-published data (Bacon et al., 2018; Bourgon et al. 2020), with associated $\delta^{13}C_{apatite}$, $\delta^{13}C_{carbon\ source}$ and $\delta^{18}O_{apatite}$values, as well as body mass and $\delta^{13}C$ (‰) Enrichment Factor used to obtain $\delta^{13}C_{carbon\ source}$. (*) The incisor NL 433 has been identified by using palaeoproteomics (Bacon et al., 2021).

Number	Country	Site	Taxon	Body Mass (kg)	$\delta^{13}C_{apatite}$ (‰ VPDB)	$\delta^{13}C$ (‰) Enrichment Factor	$\delta^{13}C_{carbon\ source}$ (‰ VPDB)	$\delta^{18}O_{apatite}$ (‰ VPDB)
NL-8	Laos	Nam Lot	*Capricornis sumatraensis*	112	-14.6	13.14	-27.7	-4.1
NL-9	Laos	Nam Lot	*Capricornis sumatraensis*	112	-13.0	13.14	-26.1	-3.3
NL-17	Laos	Nam Lot	*Rusa unicolor*	220	-12.9	13.59	-26.5	-8.7
NL-19	Laos	Nam Lot	*Rusa unicolor*	220	-7.5	13.59	-21.1	-5.8
NL-29	Laos	Nam Lot	*Rusa unicolor*	220	-14.1	13.59	-27.7	-6.7
NL-22	Laos	Nam Lot	*Rusa unicolor*	220	-0.9	13.59	-14.5	-5.5
NL-24	Laos	Nam Lot	*Rusa unicolor*	220	-2.7	13.59	-16.3	-6.1
NL-63-1	Laos	Nam Lot	*Muntiacus muntjak*	24	-13.4	12.17	-25.6	-6.4
NL-65-1	Laos	Nam Lot	*Muntiacus muntjak*	24	-13.8	12.17	-26.0	-7.3
NL-69	Laos	Nam Lot	*Muntiacus muntjak*	24	-13.9	12.17	-26.1	-3.3
NL-116	Laos	Nam Lot	Bovidae indet.	875	-9.8	14.57	-24.4	-5.9
NL-117	Laos	Nam Lot	Bovidae indet.	875	+0.3	14.57	-14.3	-4.9
NL-125	Laos	Nam Lot	Bovidae indet.	875	-13.5	14.57	-28.1	-4.7
NL-161	Laos	Nam Lot	Rhinocerotidae indet.	1633	-13.3	14.25	-27.6	-5.9
NL-162	Laos	Nam Lot	Rhinocerotidae indet.	1633	-13.1	14.25	-27.4	-2.5
NL-254-1-1	Laos	Nam Lot	Rhinocerotidae indet.	1633	-12.7	14.25	-27.0	-6.7
NL-256-1	Laos	Nam Lot	Rhinocerotidae indet.	1633	-15.1	14.25	-29.4	-6.6
NL-256-2	Laos	Nam Lot	Rhinocerotidae indet.	1633	-16.3	14.25	-30.6	-4.9
NL-256-3	Laos	Nam Lot	Rhinocerotidae indet.	1633	-15.1	14.25	-29.4	-6.7
NL-139	Laos	Nam Lot	*Bubalus bubalis*	1000	-4.7	14.66	-19.4	-6.5
NL-143	Laos	Nam Lot	*Bubalus bubalis*	1000	-9.5	14.66	-24.2	-4.4
NL-186	Laos	Nam Lot	*Ailuropoda melanoleuca*	92	-14.5	10.51	-25.0	-6.2
NL-277	Laos	Nam Lot	*Ailuropoda melanoleuca*	92	-14.9	10.51	-25.4	-4.2
NL-162	Laos	Nam Lot	*Sus* sp.	137	-13.7	13.19	-26.9	-5.7
NL-208	Laos	Nam Lot	*Sus* sp.	137	-12.4	13.19	-25.6	-6.0
NL-216	Laos	Nam Lot	*Sus* sp.	137	-12.8	13.19	-26.0	-5.3
NL-218	Laos	Nam Lot	*Sus* sp.	137	-14.1	13.19	-27.3	-6.8
NL-SS-1	Laos	Nam Lot	*Sus* sp.	137	-13.1	13.19	-26.3	-6.0

NL-258	Laos	Nam Lot	*Tapirus* sp.	300	-17.5	13.5	-31.0	-6.4
NL-259	Laos	Nam Lot	*Tapirus* sp.	300	-14.9	13.5	-28.4	-6.8
NL-260	Laos	Nam Lot	*Tapirus* sp.	300	-15.3	13.5	-28.8	-5.0
NL-286	Laos	Nam Lot	*Cuon alpinus*	15	-13.3	13.39	-26.7	-6.4
NL-368	Laos	Nam Lot	*Cuon alpinus*	15	-13.0	13.39	-26.4	-3.0
NL-269	Laos	Nam Lot	*Ursus thibetanus* cf. *kokeni*	100	-15.3	13.3	-28.6	-9.0
NL-271	Laos	Nam Lot	*Ursus thibetanus* cf. *kokeni*	100	-14.1	13.3	-27.4	-8.3
NL-275	Laos	Nam Lot	*Ursus thibetanus* cf. *kokeni*	100	-12.6	13.3	-25.9	-6.5
NL-310	Laos	Nam Lot	*Ursus thibetanus* cf. *kokeni*	100	-13.4	13.3	-26.7	-3.1
NL-288	Laos	Nam Lot	*Crocuta crocuta*	70	-13.7	14.04	-27.7	-6.4
NL-295	Laos	Nam Lot	*Crocuta crocuta*	70	-13.6	14.04	-27.6	-6.1
NL-433*	Laos	Nam Lot	*Pongo* sp.	55	-14.5	12.78	-27.3	-3.3
NL-302	Laos	Nam Lot	*Pongo* sp.	55	-14.5	12.78	-27.3	-6.1
NL-297	Laos	Nam Lot	*Macaca* sp.	6	-14.1	11.91	-26.0	-4.8
NL-314	Laos	Nam Lot	*Macaca* sp.	6	-11.5	11.91	-23.4	-3.7
NL-323	Laos	Nam Lot	*Macaca* sp.	6	-14.8	11.91	-26.7	-6.1
NL-357	Laos	Nam Lot	*Macaca* sp.	6	-14.6	11.91	-26.5	-3.6
NL-362	Laos	Nam Lot	*Elephas* sp.	4250	-16.1	14.69	-30.8	-6.2
NL-365	Laos	Nam Lot	*Stegodon orientalis*	4000	-18.2	14.66	-32.9	-4.3
NL-367	Laos	Nam Lot	*Stegodon orientalis*	4000	-15.4	14.66	-30.1	-6.5
NL-369	Laos	Nam Lot	*Hystrix* sp.	12	-13.6	12.18	-25.8	-6.5
NL-385	Laos	Nam Lot	*Hystrix* sp.	12	-12.1	12.18	-24.3	-5.7
NL-392	Laos	Nam Lot	*Hystrix* sp.	12	-14.1	12.18	-26.3	-5.2
NL-397	Laos	Nam Lot	*Hystrix* sp.	12	-13.3	12.18	-25.5	-5.7
NLII-1	Laos	Nam Lot	*Crocuta crocuta*	70	-14.3	14.04	-28.3	-6.6
NLII-2	Laos	Nam Lot	*Crocuta crocuta*	70	-12.5	14.04	-26.5	-6.8
NLII-3	Laos	Nam Lot	*Crocuta crocuta*	70	-15.1	14.04	-29.1	-6.2
NLII-4	Laos	Nam Lot	*Crocuta crocuta*	70	-11.8	14.04	-25.8	-7.2
NLII-5	Laos	Nam Lot	Felidae (*Neofelis nebulosa* ?)	18	-15.0	13.46	-28.5	-6.6
MI-20	Laos	Tam Hay Marklot	*Capricornis* cf. *sumatraensis*	112	-13.4	13.14	-26.5	-2.2
MI-21	Laos	Tam Hay Marklot	*Capricornis* cf. *sumatraensis*	112	-14.7	13.14	-27.8	-5.2
MI-22	Laos	Tam Hay Marklot	*Capricornis* cf. *sumatraensis*	112	-15.2	13.14	-28.3	-5.2
MI-23	Laos	Tam Hay Marklot	*Capricornis* cf. *sumatraensis*	112	-13.5	13.14	-26.6	-7.0
MI-24	Laos	Tam Hay Marklot	*Capricornis* cf. *sumatraensis*	112	-14.6	13.14	-27.7	-7.6
MI-25	Laos	Tam Hay Marklot	*Naemorhedus* cf. *caudatus*	27	-2.4	12.24	-14.6	0.2

MI-26	Laos	Tam Hay Marklot	*Naemorhedus* cf. *caudatus*	27	-3.9	12.24	-16.1	-2.5
MI-27	Laos	Tam Hay Marklot	*Naemorhedus* cf. *caudatus*	27	-3.7	12.24	-15.9	-1.8
MI-28	Laos	Tam Hay Marklot	*Naemorhedus* cf. *caudatus*	27	-2.5	12.24	-14.7	-1.6
MI-103	Laos	Tam Hay Marklot	*Helarctos malayanus*	50	-14.7	12.59	-27.3	-3.9
MI-121	Laos	Tam Hay Marklot	*Helarctos malayanus*	50	-14.9	12.59	-27.5	-5.5
MI-117	Laos	Tam Hay Marklot	*Ursus thibetanus*	100	-15.4	13.3	-28.7	-7.4
MI-119	Laos	Tam Hay Marklot	*Ursus thibetanus*	100	-13.3	13.3	-26.6	-6.6
MI-122	Laos	Tam Hay Marklot	*Ursus thibetanus*	100	-14.4	13.3	-27.7	-6.3
MI-134	Laos	Tam Hay Marklot	*Panthera pardus*	41	-7.9	13.81	-21.7	-7.3
MI-135	Laos	Tam Hay Marklot	*Panthera pardus*	41	-4.0	13.81	-17.8	-7.3
MI-136	Laos	Tam Hay Marklot	*Panthera pardus*	41	-13.8	13.81	-27.6	-6.8
MI-166	Laos	Tam Hay Marklot	*Rusa unicolor*	220	-7.5	13.59	-21.1	-5.7
MI-180	Laos	Tam Hay Marklot	*Rusa unicolor*	220	-5.3	13.59	-18.9	-4.4
MI-185	Laos	Tam Hay Marklot	*Rusa unicolor*	220	-6.0	13.59	-19.6	-4.9
MI-187	Laos	Tam Hay Marklot	*Rusa unicolor*	220	-3.4	13.59	-17.0	-4.9
MI-191	Laos	Tam Hay Marklot	*Rusa unicolor*	220	-7.9	13.59	-21.5	-6.1
MI-512	Laos	Tam Hay Marklot	*Rucervus eldii*	123	+2.4	13.19	-10.8	-5.3
MI-595	Laos	Tam Hay Marklot	*Rucervus eldii*	123	+1.8	13.19	-11.4	-3.1
MI-556	Laos	Tam Hay Marklot	*Axis* cf. *porcinus*	43	-0.8	12.53	-13.3	-5.8
MI-557	Laos	Tam Hay Marklot	*Axis* cf. *porcinus*	43	-0.6	12.53	-13.1	-5.3
MI-627	Laos	Tam Hay Marklot	*Muntiacus* sp.	24	-13.8	12.17	-26.0	-7.4
MI-628	Laos	Tam Hay Marklot	*Muntiacus* sp.	24	-14.8	12.17	-27.0	-8.1
MI-629	Laos	Tam Hay Marklot	*Muntiacus* sp.	24	-12.7	12.17	-24.9	-8.1
MI-630	Laos	Tam Hay Marklot	*Muntiacus* sp.	24	-14.4	12.17	-26.6	-5.0
MI-631	Laos	Tam Hay Marklot	*Muntiacus* sp.	24	-14.5	12.17	-26.7	-7.5
MI-650	Laos	Tam Hay Marklot	*Bubalus bubalis*	1000	-10.3	14.66	-25.0	-6.0
MI-651	Laos	Tam Hay Marklot	*Bubalus bubalis*	1000	-4.0	14.66	-18.7	-6.6
MI-652	Laos	Tam Hay Marklot	*Bubalus bubalis*	1000	+1.0	14.66	-13.7	-4.7
MI-653	Laos	Tam Hay Marklot	*Bubalus bubalis*	1000	-10.9	14.66	-25.6	-6.3
MI-654	Laos	Tam Hay Marklot	*Bubalus bubalis*	1000	+0.1	14.66	-14.6	-5.9
MI-655	Laos	Tam Hay Marklot	*Bos* sp.	800	-2.5	14.5	-17.0	-4.2
MI-656	Laos	Tam Hay Marklot	*Bos* sp.	800	-14.4	14.5	-28.9	-6.1
MI-657	Laos	Tam Hay Marklot	*Bos* sp.	800	-0.3	14.5	-14.8	-4.6
MI-658	Laos	Tam Hay Marklot	*Bos* sp.	800	-10.0	14.5	-24.5	-7.6
MI-659	Laos	Tam Hay Marklot	*Bos* sp.	800	-13.1	14.5	-27.6	-5.9

MI-130	Laos	Tam Hay Marklot	*Panthera tigris*	212	-4.3	14.53	-18.8	-3.2
MI-693	Laos	Tam Hay Marklot	*Panthera tigris*	212	-6.9	14.53	-21.4	-3.5
MI-694	Laos	Tam Hay Marklot	*Panthera tigris*	212	-10.0	14.53	-24.5	-6.8
MI-662	Laos	Tam Hay Marklot	*Sus* sp.	137	-7.6	13.19	-20.8	-7.5
MI-663	Laos	Tam Hay Marklot	*Sus* sp.	137	-8.6	13.19	-21.8	-10.0
MI-664	Laos	Tam Hay Marklot	*Sus* sp.	137	-6.0	13.19	-19.2	-5.9
MI-665	Laos	Tam Hay Marklot	*Sus* sp.	137	-13.5	13.19	-26.7	-5.8
MI-666	Laos	Tam Hay Marklot	*Sus* sp.	137	-14.0	13.19	-27.2	-7.6
MI-667	Laos	Tam Hay Marklot	*Sus* sp.	137	-13.2	13.19	-26.4	-5.4
MI-131	Laos	Tam Hay Marklot	*Cuon alpinus*	15	-16.0	13.39	-29.4	-7.3
MI-681	Laos	Tam Hay Marklot	*Cuon alpinus*	15	-11.4	13.39	-24.8	-3.2
MI-683	Laos	Tam Hay Marklot	*Pongo* sp.	55	-14.8	12.78	-27.6	-4.1
MI-685	Laos	Tam Hay Marklot	*Pongo* sp.	55	-13.5	12.78	-26.3	-4.6
MI-682	Laos	Tam Hay Marklot	Canidae	15	-13.2	13.39	-26.6	-6.0
MI-684	Laos	Tam Hay Marklot	*Ailuropoda melanoleuca*	92	-16.7	10.51	-27.2	-6.3
MI-691	Laos	Tam Hay Marklot	Tapiridae	300	-15.5	13.5	-29.0	-5.9
MI-692	Laos	Tam Hay Marklot	*Tapirus* sp.	300	-11.3	13.5	-24.8	-7.9
MI-695	Laos	Tam Hay Marklot	*Macaca* sp.	6	-13.9	11.91	-25.8	-5.3
MI-696	Laos	Tam Hay Marklot	*Macaca* sp.	6	-14.2	11.91	-26.1	-5.3
MI-697	Laos	Tam Hay Marklot	*Macaca* sp.	6	-15.1	11.91	-27.0	-5.6
MI-698	Laos	Tam Hay Marklot	*Macaca* sp.	6	-13.3	11.91	-25.2	-4.7
MI-699	Laos	Tam Hay Marklot	*Macaca* sp.	6	-12.9	11.91	-24.8	-4.6
MI-700	Laos	Tam Hay Marklot	*Hystrix* sp.	12	-11.5	12.18	-23.7	-7.6
MI-701	Laos	Tam Hay Marklot	*Hystrix* sp.	12	-11.5	12.18	-23.7	-5.5
MI-702	Laos	Tam Hay Marklot	*Hystrix* sp.	12	-7.9	12.18	-20.1	-5.2
MI-703	Laos	Tam Hay Marklot	*Hystrix* sp.	12	-11.6	12.18	-23.8	-8.5
MI-686	Laos	Tam Hay Marklot	*Rhinoceros sondaicus*	1750	-16.2	14.28	-30.5	-6.5
MI-687	Laos	Tam Hay Marklot	*Rhinoceros sondaicus*	1750	-13.3	14.28	-27.6	-5.5
MI-688	Laos	Tam Hay Marklot	*Rhinoceros sondaicus*	1750	-15.6	14.28	-29.9	-6.3
MI-689	Laos	Tam Hay Marklot	*Rhinoceros sondaicus*	1750	-15.3	14.28	-29.6	-7.1
MI-690	Laos	Tam Hay Marklot	*Rhinoceros sondaicus*	1750	-14.6	14.28	-28.9	-7.5

Annex S5: *Stegodon trigonocephalus* specimens from Trinil HK (in situ von Koenigswald collection) published by Puspaningrum et al. (2020), with associated $\delta^{13}C_{apatite}$, $\delta^{13}C_{carbon\ source}$ and $\delta^{18}O_{apatite}$values, as well as body mass and $\delta^{13}C$ (‰), and enrichment factor used to obtain $\delta^{13}C_{carbon\ source}$ (S-EVA, Stable Isotope-Evolutionary Anthropology, Max Planck Institute for Evolutionary Anthropology). The body mass of *S. trigonocephalus* has an estimated bodymass based on femur length of between 2,000 and 4,000 kg (Puspaningrum et al., 2020).

	**Country**	**Site**	**Taxon**	**Body mass (kg)**	**$\delta^{13}C_{apatite}$ (‰)**	**$\delta^{18}O_{apatite}$ (‰)**	**$\delta^{13}C$ (‰) Enrichment factor**	**$\delta^{13}C_{carbon\ source}$ (‰)**
K390	Java	Trinil	*Stegodon trigonocephalus*	2000-4000	+1.40	-5.71	14.53	-13.13
K399	Java	Trinil	*Stegodon trigonocephalus*	2000-4000	-1.76	-5.74	14.53	-16.29
DUB-2895	Java	Trinil	*Stegodon trigonocephalus*	2000-4000	+0.73	-6.68	14.53	-13.80
DUB-2225	Java	Trinil	*Stegodon trigonocephalus*	2000-4000	+0.11	-6.13	14.53	-14.42
DUB-379	Java	Trinil	*Stegodon trigonocephalus*	2000-4000	+0.27	-5.52	14.53	-14.26
DUB-389	Java	Trinil	*Stegodon trigonocephalus*	2000-4000	+0.99	-6.29	14.53	-13.54
DUB-3491	Java	Trinil	*Stegodon trigonocephalus*	2000-4000	-0.34	-6.4	14.53	-14.87
DUB-3253	Java	Trinil	*Stegodon trigonocephalus*	2000-4000	-2.67	-5.64	14.53	-17.20
DUB-1803A	Java	Trinil	*Stegodon trigonocephalus*	2000-4000	-2.34	-6.21	14.53	-16.87
DUB-2896	Java	Trinil	*Stegodon trigonocephalus*	2000-4000	-0.31	-6.72	14.53	-14.84

Annex S6: Mammalian taxa from Trinil HK (in situ von Koenigswald collection), Ngandong and Punung published by Janssen et al. (2016), with associated $\delta^{13}C_{apatite}$, $\delta^{13}C_{carbon\ source}$ and $\delta^{18}O_{apatite}$values, as well as body mass and $\delta^{13}C$ (‰), and enrichment factor used to obtain $\delta^{13}C_{carbon\ source}$ (S-EVA, Stable Isotope-Evolutionary Anthropology, Max Planck Institute for Evolutionary Anthropology). *Duboisia santeng* has an average bodymass of 60.3kg (95%CI: 58.9-61.7 kg; Wibono. 2020). *Bubalus palaeokerabau* has an average bodymass of ~1000 kg comparable to that of extant buffaloes (*B. bubalis/B. arnee*) (Bouteaux, 2005; Rozzi, 2018). *Axis lydekkeri* has a bodymass estimate comprised between 45 and 100 kg (Bouteaux, 2005). The mean bodymass estimate of *Panthera tigris* (pre-Ngandong samples) is 114 kg (Volmer et al., 2016). We used a bodymass of 40-150 kg for *Sus brachygnathus* comparable to that of *Sus barbatus*.

The values $\delta^{13}C$ and $\delta^{18}O$ values from enamel of the *Homo erectus* specimen (S7-37, a right upper P4) used here are average values calculated from three samples analysed on the crown (Kubat et al., 2023). The age and the location in the stratigraphy of the tooth are unknow (Early Pleistocene to early Middle Pleistocene from the Sangiran and Bapang Formations from the Sangiran Dome).

	**Country**	**Site**	**Taxon**	**Body mass (kg)**	**$\delta^{13}C_{apatite}$ (‰)**	**$\delta^{18}O_{apatite}$ (‰)**	**$\delta^{13}C$ (‰) Enrichment factor**	**$\delta^{13}C_{carbon\ source}$ (‰)**
Trinilbox-01-A	Java	Trinil	Bovidae indet.	875	+1.89	-2.80	14.57	-12.68
Trinilbox-01-A	Java	Trinil	Bovidae indet.	875	+2.49	-2.68	14.57	-12.08
Trinilbox-01-A	Java	Trinil	Bovidae indet.	875	+1.59	-2.51	14.57	-12.98
Trinilbox-02-A	Java	Trinil	Bovidae indet.	875	+1.20	-5.30	14.57	-13.37
Trinilbox-02-B	Java	Trinil	Bovidae indet.	875	+1.37	-3.70	14.57	-13.20
Trinilbox-02-C	Java	Trinil	Bovidae indet.	875	+2.16	-3.92	14.57	-12.41
Trinilbox-03-A	Java	Trinil	Bovidae indet.	875	+3.62	-2.40	14.57	-10.95
Trinilbox-03-B	Java	Trinil	Bovidae indet.	875	+4.15	-1.90	14.57	-10.42
Trinilbox-03-C	Java	Trinil	Bovidae indet.	875	+3.78	-3.16	14.57	-10.79
Trinilbox-04-A	Java	Trinil	Bovidae indet.	875	+1.91	-5.30	14.57	-12.66
Trinilbox-04-B	Java	Trinil	Bovidae indet.	875	+3.53	-3.75	14.57	-11.04
Trinilbox-04-C	Java	Trinil	Bovidae indet.	875	+2.98	-4.56	14.57	-11.59
Trinilbox-05-A	Java	Trinil	Bovidae indet.	875	+2.78	-3.62	14.57	-11.79
Trinilbox-05-B	Java	Trinil	Bovidae indet.	875	+3.32	-1.96	14.57	-11.25
Trinilbox-05-C	Java	Trinil	Bovidae indet.	875	+3.00	-3.29	14.57	-11.57
Trinilbox-06-A	Java	Trinil	Bovidae indet.	875	-3.32	-5.10	14.57	-17.89

Trinilbox-06-B	Java	Trinil	Bovidae indet.	875	+1.75	-4.38	14.57	-12.82
Trinilbox-06-C	Java	Trinil	Bovidae indet.	875	+0.98	-4.90	14.57	-13.59
Trinilbox-07-B	Java	Trinil	Bovidae indet.	875	+0.18	-5.47	14.57	-14.39
Trinilbox-07-C	Java	Trinil	Bovidae indet.	875	+1.98	-5.25	14.57	-12.59
Trinilbox-08-A	Java	Trinil	Bovidae indet.	875	-1.24	-6.74	14.57	-15.81
Trinilbox-08-B	Java	Trinil	Bovidae indet.	875	-0.30	-6.06	14.57	-14.87
Trinilbox-08-C	Java	Trinil	Bovidae indet.	875	+0.19	-6.29	14.57	-14.38
Trinilbox-09-A	Java	Trinil	Bovidae indet.	875	-0.41	-6.60	14.57	-14.98
Trinilbox-09-B	Java	Trinil	Bovidae indet.	875	+0.38	-6.37	14.57	-14.19
Trinilbox-09-C	Java	Trinil	Bovidae indet.	875	+0.91	-5.81	14.57	-13.66
Trinilbox-10-A	Java	Trinil	Bovidae indet.	875	-1.28	-6.16	14.57	-15.85
Trinilbox-10-B	Java	Trinil	Bovidae indet.	875	-3.49	-5.78	14.57	-18.06
Trinilbox-10-C	Java	Trinil	Bovidae indet.	875	-2.36	-5.40	14.57	-16.93
Trinilbox-11-A	Java	Trinil	Bovidae indet.	875	-0.39	-6.64	14.57	-14.96
Trinilbox-11-B	Java	Trinil	Bovidae indet.	875	-0.37	-6.68	14.57	-14.94
Trinilbox-11-C	Java	Trinil	Bovidae indet.	875	-1.12	-6.15	14.57	-15.69
Trinilbox-12-A	Java	Trinil	Bovidae indet.	875	+1.44	-4.90	14.57	-13.13
Trinilbox-12-B	Java	Trinil	Bovidae indet.	875	+0.90	-4.41	14.57	-13.67
Trinilbox-12-C	Java	Trinil	Bovidae indet.	875	+1.10	-4.00	14.57	-13.47
Trinilbox-13-A	Java	Trinil	Bovidae indet.	875	+2.71	-4.49	14.57	-11.86
Trinilbox-13-B	Java	Trinil	Bovidae indet.	875	+3.15	-2.89	14.57	-11.42
Trinilbox-13-C	Java	Trinil	Bovidae indet.	875	+2.30	-3.74	14.57	-12.27
Trinilbox-14-A	Java	Trinil	Bovidae indet.	875	+1.43	-1.90	14.57	-13.14
Trinilbox-14-B	Java	Trinil	Bovidae indet.	875	+1.22	-3.02	14.57	-13.35
RGM1283214	Java	Trinil	Bovidae indet.	875	+2.80	-6.33	14.57	-11.77
RGM1283215	Java	Trinil	Bovidae indet.	875	+1.16	-4.82	14.57	-13.41
RGM1283216	Java	Trinil	Bovidae indet.	875	-1.27	-5.55	14.57	-15.84
RGM1283217	Java	Trinil	Bovidae indet.	875	-2.42	-4.14	14.57	-16.99
RGM1283218	Java	Trinil	Bovidae indet.	875	-1.39	-6.28	14.57	-15.96
RGM1283219	Java	Trinil	Bovidae indet.	875	-1.83	-4.94	14.57	-16.40
RGM1283220	Java	Trinil	Bovidae indet.	875	-0.76	-5.66	14.57	-15.33
S213-A	Java	Trinil	*Bubalus palaeokerabau*	1000	+0.04	-4.37	14.66	-14.62
S213-B	Java	Trinil	*Bubalus palaeokerabau*	1000	-0.15	-5.65	14.66	-14.81

S213-C	Java	Trinil	*Bubalus palaeokerabau*	1000	-0.42	-5.75	14.66	-15.08
S213-D	Java	Trinil	*Bubalus palaeokerabau*	1000	-0.36	-5.53	14.66	-15.02
S213-E	Java	Trinil	*Bubalus palaeokerabau*	1000	-0.38	-5.14	14.66	-15.04
S213-F	Java	Trinil	*Bubalus palaeokerabau*	1000	-0.55	-4.82	14.66	-15.21
S213-G	Java	Trinil	*Bubalus palaeokerabau*	1000	-0.67	-5.01	14.66	-15.33
S213-H	Java	Trinil	*Bubalus palaeokerabau*	1000	-0.82	-4.89	14.66	-15.48
S213-I	Java	Trinil	*Bubalus palaeokerabau*	1000	-1.09	-4.48	14.66	-15.75
S2136-J	Java	Trinil	*Bubalus palaeokerabau*	1000	-1.05	-3.86	14.66	-15.71
S213-K	Java	Trinil	*Bubalus palaeokerabau*	1000	-1.65	-3.42	14.66	-16.31
S213-L	Java	Trinil	*Bubalus palaeokerabau*	1000	-1.97	-3.32	14.66	-16.63
S213-M	Java	Trinil	*Bubalus palaeokerabau*	1000	-0.34	-5.51	14.66	-15.00
S21-A	Java	Trinil	*Bubalus palaeokerabau*	1000	-0.37	-2.52	14.66	-15.03
S21-B	Java	Trinil	*Bubalus palaeokerabau*	1000	-0.27	-1.41	14.66	-14.93
S21-C	Java	Trinil	*Bubalus palaeokerabau*	1000	-0.21	-0.49	14.66	-14.87
S21-D	Java	Trinil	*Bubalus palaeokerabau*	1000	-0.61	+0.23	14.66	-15.27
S21-E	Java	Trinil	*Bubalus palaeokerabau*	1000	-0.92	+0.29	14.66	-15.58
S21-F	Java	Trinil	*Bubalus palaeokerabau*	1000	-1.15	+0.98	14.66	-15.81
S273-A	Java	Trinil	*Bubalus palaeokerabau*	1000	+0.41	-3.30	14.66	-14.25
S273-B	Java	Trinil	*Bubalus palaeokerabau*	1000	+0.15	-3.68	14.66	-14.51
S273-C	Java	Trinil	*Bubalus palaeokerabau*	1000	+0.15	-3.83	14.66	-14.51
S273-D	Java	Trinil	*Bubalus palaeokerabau*	1000	-0.04	-4.20	14.66	-14.70
S273-E	Java	Trinil	*Bubalus palaeokerabau*	1000	-0.08	-4.01	14.66	-14.74
S334-A	Java	Trinil	*Bubalus palaeokerabau*	1000	-2.66	-7.98	14.66	-17.32
S334-B	Java	Trinil	*Bubalus palaeokerabau*	1000	-1.20	-4.92	14.66	-15.86
S600-A	Java	Trinil	*Bubalus palaeokerabau*	1000	+0.80	-5.37	14.66	-13.86
S600-B	Java	Trinil	*Bubalus palaeokerabau*	1000	+0.09	-6.00	14.66	-14.57
S600-C	Java	Trinil	*Bubalus palaeokerabau*	1000	-0.20	-4.54	14.66	-14.86
S600-D	Java	Trinil	*Bubalus palaeokerabau*	1000	-0.86	-4.86	14.66	-15.52
S600-E	Java	Trinil	*Bubalus palaeokerabau*	1000	-0.10	-3.65	14.66	-14.76
S729-A	Java	Trinil	*Bubalus palaeokerabau*	1000	+0.49	-3.76	14.66	-14.17
S729-B	Java	Trinil	*Bubalus palaeokerabau*	1000	-0.86	-4.51	14.66	-15.52
S729-C	Java	Trinil	*Bubalus palaeokerabau*	1000	+0.53	-5.00	14.66	-14.13
S729-D	Java	Trinil	*Bubalus palaeokerabau*	1000	+0.20	-2.20	14.66	-14.46

S729-E	Java	Trinil	*Bubalus palaeokerabau*	1000	-0.26	-4.79	14.66	-14.92
S734-A	Java	Trinil	*Bubalus palaeokerabau*	1000	+0.27	-4.28	14.66	-14.39
S734-B	Java	Trinil	*Bubalus palaeokerabau*	1000	+0.20	-4.62	14.66	-14.46
S734-C	Java	Trinil	*Bubalus palaeokerabau*	1000	+0.37	-4.98	14.66	-14.29
S734-D	Java	Trinil	*Bubalus palaeokerabau*	1000	-0.14	-4.79	14.66	-14.80
S734-E	Java	Trinil	*Bubalus palaeokerabau*	1000	+0.49	-3.87	14.66	-14.17
S734-F	Java	Trinil	*Bubalus palaeokerabau*	1000	-0.16	-5.18	14.66	-14.82
S734-G	Java	Trinil	*Bubalus palaeokerabau*	1000	+0.08	-5.09	14.66	-14.58
S734-H	Java	Trinil	*Bubalus palaeokerabau*	1000	+0.10	-3.77	14.66	-14.56
S734-I	Java	Trinil	*Bubalus palaeokerabau*	1000	+0.01	-3.58	14.66	-14.65
S781-A	Java	Trinil	*Bubalus palaeokerabau*	1000	+0.03	-5.28	14.66	-14.63
S781-B	Java	Trinil	*Bubalus palaeokerabau*	1000	+0.25	-5.21	14.66	-14.41
S781-C	Java	Trinil	*Bubalus palaeokerabau*	1000	+0.42	-4.66	14.66	-14.24
S781-D	Java	Trinil	*Bubalus palaeokerabau*	1000	+0.22	-4.56	14.66	-14.44
S781-E	Java	Trinil	*Bubalus palaeokerabau*	1000	+0.29	-4.79	14.66	-14.37
S781-F	Java	Trinil	*Bubalus palaeokerabau*	1000	-0.05	-5.24	14.66	-14.71
S781-G	Java	Trinil	*Bubalus palaeokerabau*	1000	-0.23	-4.22	14.66	-14.89
S781-H	Java	Trinil	*Bubalus palaeokerabau*	1000	+0.35	-4.49	14.66	-14.31
S781-I	Java	Trinil	*Bubalus palaeokerabau*	1000	+0.18	-4.53	14.66	-14.48
S781-J	Java	Trinil	*Bubalus palaeokerabau*	1000	-3.33	-9.11	14.66	-17.99
S781-K	Java	Trinil	*Bubalus palaeokerabau*	1000	-1.66	-7.43	14.66	-16.32
S782-A	Java	Trinil	*Bubalus palaeokerabau*	1000	-8.63	-3.60	14.66	-23.29
S782-B	Java	Trinil	*Bubalus palaeokerabau*	1000	-7.61	-4.69	14.66	-22.27
S782-C	Java	Trinil	*Bubalus palaeokerabau*	1000	-8.82	-3.87	14.66	-23.48
T1	Java	Trinil	*Bubalus palaeokerabau*	1000	-1.50	-2.90	14.66	-16.16
T3	Java	Trinil	*Bubalus palaeokerabau*	1000	-1.70	-4.70	14.66	-16.36
T4	Java	Trinil	*Duboisa santeng*	60	-0.30	-5.10	12.74	-13.04
T5	Java	Trinil	*Duboisa santeng*	60	-0.10	-4.50	12.74	-12.84
T6	Java	Trinil	*Duboisa santeng*	60	+0.40	-2.70	12.74	-12.34
13750	Java	Trinil	*Axis lydekkeri*	45-100	+0.60	-4.07	12.86	-12.26
S1641-A	Java	Trinil	*Axis lydekkeri*	45-100	+0.85	-4.97	12.86	-12.01
S1641-B	Java	Trinil	*Axis lydekkeri*	45-100	+0.54	-3.86	12.86	-12.32
S364-A	Java	Trinil	*Axis lydekkeri*	45-100	-2.15	-5.02	12.86	-15.01

S364-B	Java	Trinil	*Axis lydekkeri*	45-100	-1.02	-4.54	12.86	-13.88
S364-C	Java	Trinil	*Axis lydekkeri*	45-100	-0.26	-5.36	12.86	-13.12
MB.Ma.22115	Java	Trinil	*Axis lydekkeri*	45-100	+0.57	-3.03	12.86	-12.29
S604-A	Java	Trinil	*Axis lydekkeri*	45-100	+0.02	-3.97	12.86	-12.84
S604-B	Java	Trinil	*Axis lydekkeri*	45-100	+0.44	-3.76	12.86	-12.42
S738-A	Java	Trinil	*Axis lydekkeri*	45-100	-0.31	-3.58	12.86	-13.17
S738-B	Java	Trinil	*Axis lydekkeri*	45-100	+0.77	-3.08	12.86	-12.09
1578-1-A	Java	Trinil	*Sus brachygnathus*	41-150	-3.94	-6.05	13.19	-17.13
1578-1-B	Java	Trinil	*Sus brachygnathus*	41-150	-4.18	-5.31	13.19	-17.37
1578-1-C	Java	Trinil	*Sus brachygnathus*	41-150	-4.97	-5.31	13.19	-18.16
1578-1-D	Java	Trinil	*Sus brachygnathus*	41-150	-8.94	-7.73	13.19	-22.13
1578-2	Java	Trinil	*Sus brachygnathus*	41-150	-12.77	-5.42	13.19	-25.96
1578-3	Java	Trinil	*Sus brachygnathus*	41-150	-1.96	-7.49	13.19	-15.15
MB.Ma.30002	Java	Trinil	*Panthera tigris*	114	-5.63	-6.57	14.53	-20.16
11309(K363)	Java	Ngandong	*Stegodon trigonocephalus*	2000-4000	+0.61	-6.79	14.53	-13.92
13322(K351)	Java	Ngandong	*Stegodon trigonocephalus*	2000-4000	-1.62	-6.43	14.53	-16.15
K323	Java	Ngandong	*Stegodon trigonocephalus*	2000-4000	+0.02	-6.33	14.53	-14.51
9875(K320)	Java	Ngandong	*Stegodon trigonocephalus*	2000-4000	-2.02	-6.48	14.53	-16.55
5667(K440a)	Java	Ngandong	*Stegodon trigonocephalus*	2000-4000	-5.92	-7.63	14.53	-20.45
383(K318)	Java	Ngandong	*Stegodon trigonocephalus*	2000-4000	-5.32	-6.72	14.53	-19.85
K316	Java	Ngandong	*Stegodon trigonocephalus*	2000-4000	-3.21	-6.60	14.53	-17.74
K329	Java	Ngandong	*Stegodon trigonocephalus*	2000-4000	-3.50	-7.41	14.53	-18.03
1075(K307)	Java	Ngandong	*Stegodon trigonocephalus*	2000-4000	+0.73	-7.22	14.53	-13.80
2205	Java	Ngandong	*Stegodon trigonocephalus*	2000-4000	+2.31	-6.31	14.53	-12.22
631035-1	Java	Sangiran	*Bubalus palaeokerabau*	1000	+1.05	-4.88	14.66	-13.61
631035-2-A	Java	Sangiran	*Bubalus palaeokerabau*	1000	+2.28	-3.03	14.66	-12.38
631035-2-B	Java	Sangiran	*Bubalus palaeokerabau*	1000	+2.04	-3.59	14.66	-12.62
631035-2-C	Java	Sangiran	*Bubalus palaeokerabau*	1000	+2.56	-4.50	14.66	-12.1
631035-2-D	Java	Sangiran	*Bubalus palaeokerabau*	1000	+2.44	-5.92	14.66	-12.22
631035-2-E	Java	Sangiran	*Bubalus palaeokerabau*	1000	+2.49	-5.64	14.66	-12.17
631035-2-F	Java	Sangiran	*Bubalus palaeokerabau*	1000	+2.27	-4.97	14.66	-12.39

631035-2-G	Java	Sangiran	*Bubalus palaeokerabau*	1000	+2.04	-5.11	14.66	-12.62
631035-3	Java	Sangiran	*Bubalus palaeokerabau*	1000	+3.06	-4.79	14.66	-11.6
631035-4	Java	Sangiran	*Bubalus palaeokerabau*	1000	+0.68	-5.37	14.66	-13.98
631035-5-A	Java	Sangiran	*Bubalus palaeokerabau*	1000	-6.29	-6.11	14.66	-20.95
631035-5-B	Java	Sangiran	*Bubalus palaeokerabau*	1000	-6.75	-5.31	14.66	-21.41
631035-5-C	Java	Sangiran	*Bubalus palaeokerabau*	1000	-6.98	-5.35	14.66	-21.64
631035-5-D	Java	Sangiran	*Bubalus palaeokerabau*	1000	-7.80	-5.79	14.66	-22.46
631035-5-E	Java	Sangiran	*Bubalus palaeokerabau*	1000	-7.75	-5.88	14.66	-22.41
631035-5-F	Java	Sangiran	*Bubalus palaeokerabau*	1000	-6.43	-5.55	14.66	-21.09
631035-5-G	Java	Sangiran	*Bubalus palaeokerabau*	1000	-6.26	-4.96	14.66	-20.92
631035-6	Java	Sangiran	*Bubalus palaeokerabau*	1000	+0.87	-6.22	14.66	-13.79
631035-7-A	Java	Sangiran	*Bubalus palaeokerabau*	1000	+0.59	-5.29	14.66	-14.07
631035-7-B	Java	Sangiran	*Bubalus palaeokerabau*	1000	+0.87	-5.56	14.66	-13.79
631035-7-D	Java	Sangiran	*Bubalus palaeokerabau*	1000	+1.01	-5.36	14.66	-13.65
631035-7-F	Java	Sangiran	*Bubalus palaeokerabau*	1000	+0.35	-7.32	14.66	-14.31
631035-8	Java	Sangiran	*Bubalus palaeokerabau*	1000	+1.11	-4.78	14.66	-13.55
630847-1-A	Java	Sangiran	Cervidae Indet.	45-100	+2.06	-6.07	12.86	-10.8
630847-1-B	Java	Sangiran	Cervidae Indet.	45-100	+2.16	-6.49	12.86	-10.7
630847-2-A	Java	Sangiran	Cervidae Indet.	45-100	-6.50	-4.10	12.86	-19.36
630847-2-B	Java	Sangiran	Cervidae Indet.	45-100	-5.60	-5.17	12.86	-18.46
630847-2-C	Java	Sangiran	Cervidae Indet.	41-150	-6.01	-5.46	12.86	-18.87
630847-3	Java	Sangiran	Cervidae Indet.	41-150	+0.78	-2.89	12.86	-12.08
630847-4-A	Java	Sangiran	Cervidae Indet.	41-150	-9.61	-4.87	12.86	-22.47
630847-4-B	Java	Sangiran	Cervidae Indet.	41-150	-10.63	-5.67	12.86	-23.49
630847-4-C	Java	Sangiran	Cervidae Indet.	41-150	-9.79	-5.92	12.86	-22.65
630847-5-A	Java	Sangiran	Cervidae Indet.	45-100	-1.71	-5.71	12.86	-14.57
630847-5-B	Java	Sangiran	Cervidae Indet.	45-100	-2.94	-5.79	12.86	-15.8
630847-5-C	Java	Sangiran	Cervidae Indet.	45-100	-2.97	-6.64	12.86	-15.83
630847-6-A	Java	Sangiran	Cervidae Indet.	41-150	+0.67	-4.32	12.86	-12.19
630847-6-B	Java	Sangiran	Cervidae Indet.	41-150	+0.48	-6.13	12.86	-12.38
630847-6-C	Java	Sangiran	Cervidae Indet.	41-150	+0.45	-4.86	12.86	-12.41
630847-7-A	Java	Sangiran	Cervidae Indet.	41-150	-0.12	-3.51	12.86	-12.98
630847-7-B	Java	Sangiran	Cervidae Indet.	41-150	+0.10	-4.20	12.86	-12.76

630847-7-C	Java	Sangiran	Cervidae Indet.	45-100	-0.22	-5.54	12.86	-13.08
630847-8-A	Java	Sangiran	Cervidae Indet.	45-100	-0.69	-7.10	12.86	-13.55
630847-8-B	Java	Sangiran	Cervidae Indet.	45-100	-0.38	-5.93	12.86	-13.24
630847-8-C	Java	Sangiran	Cervidae Indet.	45-100	-0.38	-5.93	12.86	-13.24
630847-9-A	Java	Sangiran	Cervidae Indet.	45-100	+0.00	-4.94	12.86	-12.86
630847-9-B	Java	Sangiran	Cervidae Indet.	45-100	-2.04	-2.47	12.86	-14.9
630847-9-C	Java	Sangiran	Cervidae Indet.	45-100	-1.32	-3.81	12.86	-14.18
630847-10-A	Java	Sangiran	Cervidae Indet.	45-100	-0.69	-4.81	12.86	-13.55
630847-10-B	Java	Sangiran	Cervidae Indet.	45-100	-1.33	-5.34	12.86	-14.19
RGM630846	Java	Sangiran	*Axis lydekkeri*	45-100	+0.43	-5.62	12.86	-12.43
631045-1-A	Java	Sangiran	*Sus* sp.	41-150	-15.26	-5.69	13.19	-28.45
631045-1-B	Java	Sangiran	*Sus* sp.	41-150	-14.18	-5.04	13.19	-27.37
631045-1-C	Java	Sangiran	*Sus* sp.	41-150	-15.31	-4.91	13.19	-28.5
631045-2	Java	Sangiran	*Sus* sp.	41-150	-0.34	-5.41	13.19	-13.53
631045-3	Java	Sangiran	*Sus* sp.	41-150	-10.55	-5.71	13.19	-23.74
631045-4	Java	Sangiran	*Sus* sp.	41-150	-11.59	-7.44	13.19	-24.78
631045-5	Java	Sangiran	*Sus* sp.	41-150	-12.52	-5.87	13.19	-25.71
631045-6-A	Java	Sangiran	*Sus* sp.	41-150	+0.83	-5.26	13.19	-12.36
631045-6-B	Java	Sangiran	*Sus* sp.	41-150	+0.96	-6.52	13.19	-12.23
631045-7	Java	Sangiran	*Sus* sp.	41-150	-2.14	-6.74	13.19	-15.33
631045-8-A	Java	Sangiran	*Sus* sp.	41-150	-6.75	-5.80	13.19	-19.94
631045-8-B	Java	Sangiran	*Sus* sp.	41-150	-6.63	-5.97	13.19	-19.82
631045-9	Java	Sangiran	*Sus* sp.	41-150	-2.66	-7.65	13.19	-15.85
631045-10	Java	Sangiran	*Sus* sp.	41-150	-11.84	-8.83	13.19	-25.03
S7-37	Java	Sangiran 7	*Homo erectus*-right upper P4	62	-3.96	-6.3	12.7	-16.66
80108	Java	Punung	Bovidae indet.	875	-13.08	-5.42	14.57	-27.65
GD110-A	Java	Punung	Bovidae indet.	875	+1.24	-4.69	14.57	-13.33
GD110-B	Java	Punung	Bovidae indet.	875	+1.18	-4.84	14.57	-13.39
GD110-C	Java	Punung	Bovidae indet.	875	+0.79	-5.43	14.57	-13.78

GD110-D	Java	Punung	Bovidae indet.	875	+0.35	-5.60	14.57	-14.22
GD110-E	Java	Punung	Bovidae indet.	875	-0.80	-4.76	14.57	-15.37
GD39	Java	Punung	*Sus* sp.	137	-10.08	-7.20	13.19	-23.27

Annex S7: Mammalian taxa from Lida Ajer published by Louys et al. (2022) with associated $\delta^{13}C_{apatite}$, $\delta^{13}C_{carbon\ source}$ and $\delta^{18}O_{apatite}$values, as well as body mass and $\delta^{13}C$ (‰), and enrichment factor used to obtain $\delta^{13}C_{carbon\ source}$ (S-EVA, Stable Isotope-Evolutionary Anthropology, Max Planck Institute for Evolutionary Anthropology).

Sample ID	Taxon	Family	Source	$\delta^{13}C$ ‰	Body mass (kg)	$\delta^{13}C$ (‰) Enrichment factor	$\delta^{13}C_{carbon\ source}$ (‰)	$\delta^{13}C_{diet}$ (‰)	$\delta^{18}O$ (‰)
L007-A	*Capricornis sumatraensis*	Bovidae	Naturalis	-15,3	112	13,14	-28,44	-29,3	-7,2
L007-B	*Capricornis sumatraensis*	Bovidae	Naturalis	-15,5	112	13,14	-28,64	-29,5	-6,5
L004-A	*Elephas maximus*	Elephantidae	Naturalis	-15,7	4250	14,69	-30,39	-29,7	-8,7
L004-B	*Elephas maximus*	Elephantidae	Naturalis	-15,7	4250	14,69	-30,39	-29,7	-8,7
L004-C	*Elephas maximus*	Elephantidae	Naturalis	-15,5	4250	14,69	-30,19	-29,5	-9
L004-D	*Elephas maximus*	Elephantidae	Naturalis	-16,2	4250	14,69	-30,89	-30,2	-8,9
L004-E	*Elephas maximus*	Elephantidae	Naturalis	-16,2	4250	14,69	-30,89	-30,2	-8,9
L004-F	*Elephas maximus*	Elephantidae	Naturalis	-15,4	4250	14,69	-30,09	-29,4	-8,7
L005	*Elephas maximus*	Elephantidae	Naturalis	-13,2	4250	14,69	-27,89	-27,2	-8,3
LA15-5	*Pongo* sp.	Hominidae	ITB (sinkhole)	-12,6	55	12,78	-25,38	-23,6	-6,5
LA15-8	*Pongo* sp.	Hominidae	ITB (Area 2)	-12,3	55	12,78	-25,08	-23,3	-7,8
L001-B	*Pongo pygmaeus*	Hominidae	Naturalis	-15	55	12,78	-27,78	-26	-6,8
LA15-1	*?Rhinoceros*	Rhinocerotidae	ITB (sinkhole)	-15,5	950	14	-29,5	-29,5	-7,4
LA15-7	*?Dicerorhinus*	Rhinocerotidae	ITB (area 2)	-17,4	1650	14,25	-31,65	-31,4	-6,5
L008-A	Rhinocerotidae	Rhinocerotidae	Naturalis	-16,8	1650	14,25	-31,05	-30,8	-8,4

L008-B	Rhinocerotidae	Rhinocerotidae	Naturalis	-16,3	1650	14,25	-30,55	-30,3	-8,2
L009	Rhinocerotidae	Rhinocerotidae	Naturalis	-16,7	1650	14,25	-30,95	-30,7	-6,3
L010	Rhinocerotidae	Rhinocerotidae	Naturalis	-16,2	1650	14,25	-30,45	-30,2	-8
L011-A	Rhinocerotidae	Rhinocerotidae	Naturalis	-16,3	1650	14,25	-30,55	-30,3	-7,6
L011-B	Rhinocerotidae	Rhinocerotidae	Naturalis	-16,6	1650	14,25	-30,85	-30,6	-7,5
L012-A	Rhinocerotidae	Rhinocerotidae	Naturalis	-17,4	1650	14,25	-31,65	-31,4	-6,6
L012-B	Rhinocerotidae	Rhinocerotidae	Naturalis	-17,3	1650	14,25	-31,55	-31,3	-6,4
L013	Rhinocerotidae	Rhinocerotidae	Naturalis	-16,9	1650	14,25	-31,15	-30,9	-8,8
LA15-3	*Sus* sp.	Suidae	ITB (sinkhole)	-11,53	137	13,19	-24,72	-22,5	-7,4
LA15-4	*Sus* sp.	Suidae	ITB (sinkhole)	-11,94	137	13,19	-25,13	-22,9	-8,2
LA15-16	*Sus* sp.	Suidae	ITB (Unit 7)	-12,31	137	13,19	-25,5	-23,3	-9,1
LA15-17	*Sus* sp.	Suidae	ITB (unit 7)	-12,02	137	13,19	-25,21	-23	-8,9
L002	*Tapirus indicus*	Tapiridae	Naturalis	-17,2	300	13,5	-30,7	-31,2	-9,3
